# Synergistic Regulation of Deep‐Cycling‐Induced Zn Pulverization and Resting Galvanic Corrosion in Practical Lean Zinc Anodes

**DOI:** 10.1002/advs.77731

**Published:** 2026-09-10

**Authors:** Jing Xu, Haolin Li, Qiyin Jing, Lingfei Zhao, Elad Ballas, Bing Sun, Doron Aurbach, Guoxiu Wang

**Affiliations:** ^1^ Research Center of Grid Energy Storage and Battery Application School of Electrical and Information Engineering Zhengzhou University Zhengzhou China; ^2^ Centre for Clean Energy Technology School of Mathematical and Physical Sciences Faculty of Science University of Technology Sydney New South Wales Australia; ^3^ Department of Chemistry and Institute of Nanotechnology & Advanced Materials (BINA) Bar‐Ilan University Ramat Gan Israel

**Keywords:** aqueous Zn metal anodes, calendar aging, deep charge‐discharge, galvanic corrosion, Zn pulverization

## Abstract

Scaling up aqueous zinc (Zn)‐ion batteries with long cycling stability and calendar life (i.e., long rest periods at open‐circuit potential) remains challenging, particularly with the use of a thick Zn foil anode. A more practical and scalable approach involves adopting a lean‐Zn configuration with a current collector. Due to the limited Zn availability, lean Zn anodes experience different failure mechanisms, manifested as Zn pulverization in cycle life and galvanic corrosion during calendar life. Unfortunately, these issues have long been overlooked. Herein, we introduce benzotriazole (BTA), a well‐known corrosion inhibitor, as an electrolyte additive in aqueous zinc sulfate (ZnSO_4_) electrolytes. BTA modifies the electric double layer (EDL) structure on the Zn anode, alleviating the mismatch between Zn^2^^+^ mass transport and reduction kinetics. This leads to the formation of dense, flat zinc deposits rather than the Zn pulverized structures, significantly reducing contact loss during stripping. On the other hand, the strong chemisorption of BTA on the Cu substrate effectively blocks electron transfer pathways responsible for galvanic corrosion, reducing capacity fade during calendar aging. When paired with a MnO_2_ cathode, the lean‐Zn anode enables the full cell to retain 90.3% of its capacity over 600 cycles at a negative: positive (N:P) ratio of 3:1.

## Graphical abstract

Benzotriazole (BTA) regulates Zn reduction kinetics and suppresses galvanic corrosion, simultaneously mitigating Zn pulverization during cycling and self‐corrosion during storage, thereby enabling lean Zn anodes with enhanced cycling stability and extended calendar life.

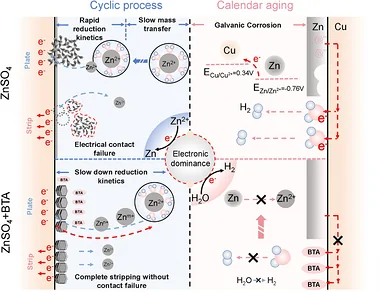

## 1 Introduction

Rechargeable aqueous Zn‐ion batteries (AZIBs) are considered promising candidates for grid‐scale energy storage, owing to the intrinsic merits of Zn metal anodes [1, 2, 3, 4], including a high theoretical specific capacity (820 mA h g^−1^), relatively low redox potential (−0.762 V vs. the standard hydrogen electrode (SHE)), low cost ($1.45 per lb), good stability toward aqueous electrolytes and environmental friendliness [5, 6]. However, they also face critical challenges that hinder their practical applications, including Zn dendrite formation, limited reversibility of Zn metal anodes, and short cycle life. Enhancing the cycling stability and improving the utilization efficiency of Zn metal anodes are crucial for developing high energy density and long service life AZIBs. Currently, thick Zn foils (>200 µm in thickness) with an areal specific capacity of more than 117.0 mA h cm^−2^ are usually employed as anodes [7, 8, 9, 10], inducing a high N/P ratio (negative/positive capacity ratio, based on the entire electrode's theoretical capacity, and do not consider Zn utilization) of more than 39, considering the areal specific capacity of the cathode is 3.0 mA h cm^−2^ [11]. This provides an excessive amount of Zn to compensate for the loss of active Zn metal due to parasitic reactions during long‐term cycling. However, this approach significantly reduces the energy density of AZIBs. Therefore, developing stable **lean Zn anodes** (i.e., Zn‐based anodes with a limited amount of Zn pre‐deposited on the current collectors) is vital to achieve practical applications of high‐energy‐density AZIBs.

**FIGURE 1:**
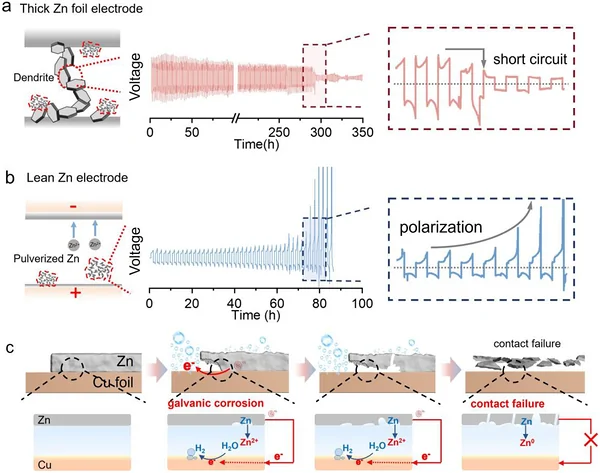
Schematic illustrations of typical failure modes in symmetric Zn cells. (a) Short‐circuit formation when using a conventional Zn foil electrode; (b) Zinc loss mode when using lean Zn electrode; and (c) Galvanic corrosion occurring in the lean Zn electrode immersed in ZnSO_4_ electrolyte.

For AZIBs with thick Zn foils as anodes, battery failure is usually caused by short circuits resulting from Zn dendrite growth after long‐term cycling (Figure 1a) [12, 13, 14]. Zn pulverization is typically not considered as the primary cause of cycling failure and has long been overlooked [15, 16, 17, 18, 19]. Unfortunately, due to the limited Zn availability, the practical application of lean Zn anodes in rechargeable Zn‐ion batteries experiences rapid capacity degradation compared to conventional thick Zn foil anodes, manifested as a swift decline in both cycle life and calendar life (i.e. self‐discharge phenomena upon rest). Specifically, lean Zn anodes experience severe pulverization during cycling even at low charge/discharge capacities (e.g., areal capacity>1 mA h cm^−2^), accompanied by parasitic reactions such as hydrogen evolution and surface passivation. These pulverized Zn [16, 17, 18, 19], with their loose and porous structure, tend to detach from the current collector during the stripping process, forming “dead Zn”, which gradually depletes the limited Zn supply and ultimately results in rapid battery failure. This phenomenon is evident in the cell's voltage curves as a sharp increase in polarization in symmetric cells (Figure 1b). Therefore, mitigating Zn pulverization is essential for extending the lifespan of AZIBs with high energy density and low N/P ratios.

On the other hand, lean Zn anodes experience significant capacity loss during calendar aging (approximately 30% per day), an order of magnitude greater than that observed in lithium‐ion batteries [20]. Although chemical corrosion is typically one cause of capacity loss during static storage for AZIBs using Zn foil anodes, galvanic corrosion becomes another critical factor leading to rapid capacity degradation in lean Zn anode configurations [20, 21], where Zn is pre‐deposited onto different current collectors. For instance, the pre‐deposited Zn and the Cu current collector form a galvanic couple due to the potential difference between these two different metals [22], spontaneously generating a corrosion cell (Figure 1c). Continuous galvanic corrosion ultimately leads to the detachment of Zn from the Cu current collector, resulting in an irreversible loss of active Zn. This significantly hinders the practical implementation of AZIBs in energy storage systems.

Herein, we introduce benzotriazole (BTA) as an electrolyte additive to enhance both the cycle stability and calendar life of lean Zn anodes by restricting reduction kinetics to suppress Zn pulverization during cycling and Zn galvanic corrosion during static conditions. We discovered that electrical isolation of metallic Zn° from the current collectors (Zn, Cu, Ti, and stainless‐steel substrates) was the primary cause of the irreversible capacity loss in lean Zn anodes, both during cycling and calendar aging. During the cycling process, inactive metallic Zn^0^‐dominant zinc loss initially results from a bottom‐preferred Zn stripping mechanism within the pulverized Zn mossy structure. When adding BTA into the electrolyte, BTA effectively prevents Zn pulverization by reducing the Zn^2+^ nucleation, and reduction kinetics during deposition. In addition, BTA effectively alleviates galvanic corrosion by inhibiting electron transfer between the Zn─Cu galvanic couple, thus significantly improving calendar life. Consequently, the shelving‐recovery tests (cycling processes comprising 10 h of repeated cycling and 6 h rest) of symmetric cells with lean Zn electrodes delivered a stable operation exceeding 300 h (25% DOD). This work unrevealed critical and fundamental insights into the origins of inactive Zn loss in lean Zn anodes during both cycling and calendar aging, providing essential guidance for developing practical high‐performance zinc‐ion batteries.

## 2 Results

### 2.1 Severe Zn Pulverization due to Fast Nucleation Kinetics

**FIGURE 2:**
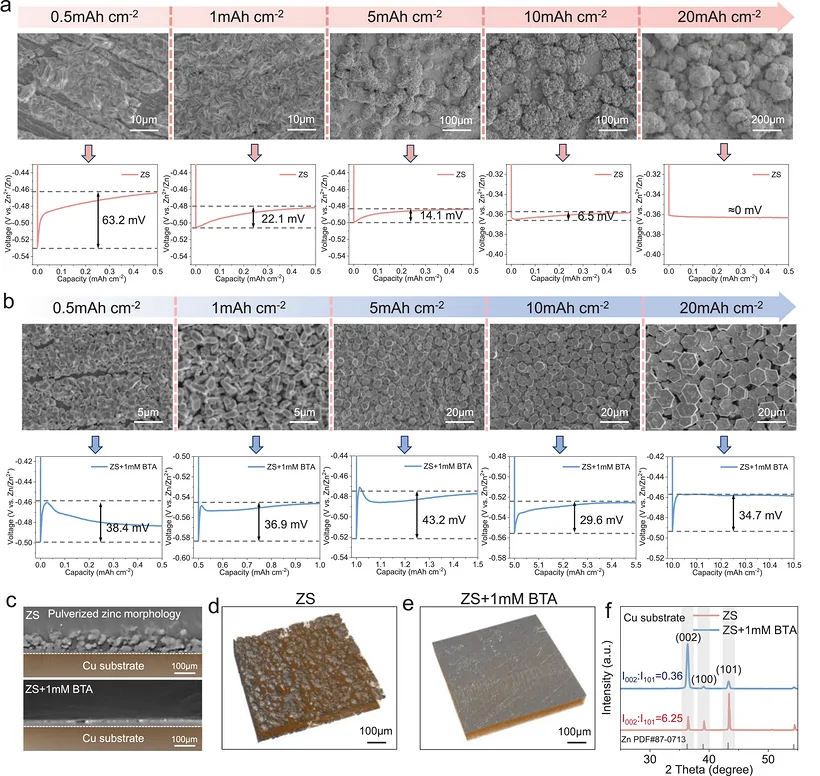
Zn deposition behaviors in the ZnSO_4_ and ZnSO_4_+BTA electrolytes. (a) SEM images of the gradual formation of pulverized Zn deposits with increasing plating capacities on a Cu substrate in 2 m ZnSO_4_ (denoted as ZS in the figures) electrolyte_._ (b) SEM images of the Zn deposits with increasing plating capacities on a Cu substrate in 2 m ZnSO_4_+1 mm BTA (denoted ZS+1 mm BTA in the figures) electrolyte. (c) Reconstructed X‐ray tomography slices of Zn deposits on Cu substrates in ZS and ZS+BTA electrolytes. (d, e) The corresponding 3D representations of the reconstructed data. (f) X‐ray diffraction (XRD) patterns of the Zn deposits on Cu substrates in the two different electrolytes.

In the beginning, we discussed the advantages of lean Zn anodes (i.e., Zn‐based anodes with a limited amount of Zn pre‐deposited on the current collectors) in the Figures S1 and S2, and finally chose lean Zn@Cu anode configuration in this work. Then we deposited Zn metal step‐by‐step with increasing capacities and no external pressure onto a Cu foil substrate in a quartz cuvette cell using a pristine 2 m ZnSO_4_ electrolyte (Figure 2a and Figures S3 and S4). Scanning electron microscope (SEM) observation was conducted to examine the morphological characteristics of the Zn deposits after each deposition step. At the initial nucleation stage (0–0.5 mA h cm^−2^), tightly‐stacked Zn flakes were formed on the Cu substrate, starting to form a seed layer (Figure 2a and Figure S3). As the deposition capacity increased from 0.5 to 1 mA h cm^−2^, the seed layer gradually covered the entire Cu substrate. Further increasing the deposition capacities from 1 to 20 mA h cm^−2^ in subsequent steps led to the formation of pulverized, spherical, and porous Zn deposits on top of the compact Zn seed layer. The original silver‐gray Zn deposits turned black with increasing deposition capacity, as shown in the digital photographs in Figure S1b. Similar phenomena were observed on Zn, Ti, and stainless‐steel (SS) substrates (Figures S5, S7, and S9). The SEM observation reveals that Zn pulverization commonly begins at low areal deposition capacities (>1.0 mA h cm^−2^), regardless of the types of substrates, posing significant challenges for the design of high‐energy‐density Zn metal batteries with lean Zn anodes.

The nucleation overpotentials at the beginning of each deposition step were also recorded and compared (Figure 2a). As shown in Figure 2a and Figure S4, the nucleation overpotentials quickly dropped from ∼ 60 mV during the first deposition step (0–0.5 mA h cm^−2^) to about 3.4 mV (average value) during the fifth deposition step (10.0–20.0 mA h cm^−2^). Similar results were obtained on Zn, Ti, and stainless‐steel (SS) substrates (**Figures** **S6**, S8, and S10). According to classical nucleation theory, the formation of a new solid phase must overcome a free energy barrier, which is associated with the nucleation overpotential [23, 24, 25]. The initial high nucleation overpotentials observed during the first and second deposition steps indicate that substantial energy is required to form Zn nuclei on the surface of the Cu substrate, which restricts Zn nucleation kinetics. At this stage, the moderate nucleation kinetics align well with the slow Zn^2+^ ion transfer rate in the electrolyte, preventing the formation of a severe Zn^2^^+^ concentration gradient and enabling Zn nuclei to grow into microflakes and form a compact seed layer on the Cu substrate. However, during the following deposition steps (1.0–20 mA h cm^−2^), the Zn nucleation overpotentials dropped sharply when pulverized Zn began to deposit over the seed layer. The decreased nucleation overpotential indicates that a weaker energy barrier is required for Zn nucleus formation, demonstrating enhanced Zn nucleation kinetics [26]. This rapid Zn nucleation rate produces numerous competing nuclei, hindering the growth of existing ones, and resulting in pulverized Zn deposits. Step‐by‐step Zn deposition investigations demonstrate that restricting Zn nucleation kinetics is essential to allow Zn nuclei to grow into compact microflakes rather than fragile, pulverized structures.

### 2.2 Restricting Zn Nucleation Kinetics With a Nucleation Inhibitor to Suppress Zn Pulverization

To promote the growth of Zn nuclei and ensure the formation of a compact deposition layer, we introduced a nucleation inhibitor (BTA) into the aqueous electrolyte (i.e., 1 mm BTA in 2 m ZnSO_4_ solution) [27, 28, 29]. This inhibitor regulates Zn nucleation kinetics, effectively preventing quick Zn pulverization. SEM observations were performed to confirm morphological changes of Zn deposits on the Cu substrate during step‐by‐step deposition in the electrolyte containing BTA. During the initial two steps (0–1.0 mA h cm^−2^), Zn deposits were formed on the Cu substrate, resulting in a homogeneous seed layer (Figure 2b and Figure S11). Afterward, hexagonal microplates were formed and stacked layer by layer on the Cu substrates in the following deposition steps (5.0–20.0 mA h cm^−2^). The Zn deposition layers were compact without pulverization. Similar morphologies were also observed on Zn, Ti, and stainless‐steel (SS) substrates (Figures S13, S15, and S17). In addition, we performed synchrotron X‐ray tomography to visually present the 3D morphology of Zn deposits on Cu substrates. (Figure 2c–e). In the ZnSO_4_ electrolyte without the additive, Zn deposits exhibit a flower‐bud‐like morphology across wide areas. In sharp contrast, under the restrictive influence of BTA, flat, and uniform Zn deposition layers were consistently observed on the Cu substrate. Notably, owing to the presence of BTA in the electrolyte, the nucleation overpotential remains stable during each deposition step. There is no significant decrease in nucleation overpotential as plating progresses, irrespective of substrate type (Figure 2b and Figures S12, S14, S16, and S18). The consistently high nucleation overpotentials observed during the step‐by‐step Zn deposition confirm that the addition of a nucleation inhibitor effectively restricts nucleation kinetics, promoting the growth of existing Zn nuclei into hexagonal microplates.

The hexagonal Zn microplates formed in the electrolyte containing BTA exhibited high crystallinity with a preferred (002) crystal orientation, as confirmed by transmission electron microscopy (TEM) (Figure S19) and X‐ray diffraction (XRD) analyses (Figure 2f and Figure S20). In contrast, Zn deposits obtained in the pristine ZnSO_4_ electrolyte exhibited poor crystallinity, characterized by a substantial presence of amorphous phases and a predominant (101) crystal orientation—a typical feature of pulverized Zn deposits. The introduction of a nucleation inhibitor significantly promotes the formation of highly crystallized, compact, and uniform Zn deposition without pulverization.

### 2.3 Impact of BTA Additive on Reaction Kinetics in the Zn Electroplating Process

**FIGURE 3:**
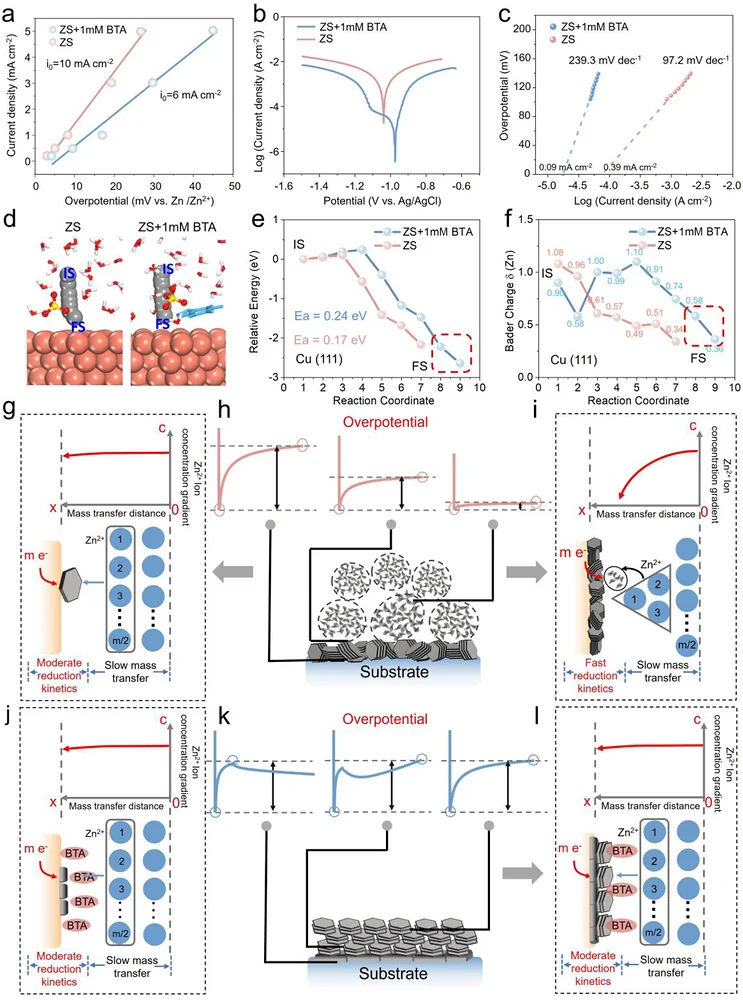
The characteristic Zn deposits behavior in the ZnSO_4_ and ZnSO_4_+BTA electrolytes. (a) Calculated exchange current density of Zn@Cu||Zn@Cu symmetric cell in ZnSO_4_ electrolytes with/without BTA. (b) Tafel polarization plots, (c) the corresponding fitted linear portions measured using RDE at a speed of 3600 rpm. (d) Zn^2+^ diffusion from the ZnSO_4_ electrolyte to Cu (111) substrate with/without BTA. (e) Relative energy and (f) Bader charge for Zn^2+^ during the diffusion in ZnSO_4_ electrolytes with/without BTA. Water is shown as stick model, SO_4_^2−^ as ball‐stick with O (rose red) and S (yellow), BTA as blue stick, and Cu substrate as coral red ball. (g) Schematic influence of concentration gradient and nucleation overpotential on the growth of deposition seed layer in ZnSO_4_ electrolyte. (h) Variation of nucleation overpotential during different plating stage in ZnSO_4_ electrolyte. (i) Schematic influence of concentration gradient and nucleation overpotential on the growth of pulverized zinc in ZnSO_4_ electrolyte. (j) Variation of nucleation overpotential during seed layer deposition in the ZnSO_4_+BTA electrolyte. (k) Schematic influence of nucleation overpotential during different plating stage in the ZnSO_4_+BTA electrolyte. (l) Schematic influence of concentration gradient in the ZnSO_4_+BTA electrolyte.

The impact of BTA additive on the reduction kinetics of Zn^2+^ ions during the electroplating process was first studied via galvanostatic cycling tests using Zn@Cu||Zn@Cu symmetric cells with different electrolytes (Figure S21). As shown in Figure 3a, the cell with ZnSO_4_+BTA electrolyte showed a lower exchange current density of 6 mA cm^−2^ compared to that of the cell without BTA additive electrolyte (10 mA cm^−2^), demonstrating that the BTA additive can effectively regulate the critical Zn^2+^ reduction kinetics. In addition, we used a rotating disk electrode (RDE) to study the reduction kinetics more accurately by eliminating the influence of mass transfer in the diffusion‐layer near the electrode. Tafel plots under a high rotation speed of 3000 rpm were obtained in different electrolytes (Figure 3b). It is well known that, a high Tafel slope and low exchange current density indicate sluggish electrochemical reduction kinetics [30, 31]. The Tafel slopes calculated for the ZnSO_4_ and ZnSO_4_+BTA electrolytes are 97.2 and 239.3 mV dec^−1^, respectively (Figure 3c). Meanwhile, a lower exchange current density was observed for the RDE electrode in the ZnSO_4_+BTA electrolyte than in the ZnSO_4_ electrolyte. Therefore, the above electrochemical testing results demonstrate that the BTA additive reduces the reduction kinetics of Zn^2+^ ions during the deposition process.

To elucidate the mechanism of BTA additive on the Zn^2+^ reduction kinetics, we investigated Zn^2^^+^ diffusion dynamics near the surface of the Cu (111) substrate in the two different electrolytes (ZnSO_4_ vs. ZnSO_4_+BTA electrolytes) via molecular dynamics (MD) simulations. The generated images of Zn^2+^ diffusion from the initial state to the final state are shown in Figure 3d. The energy of the initial state was used as a reference. The relative energy of the other seven states has been plotted in Figure 3e, showing the barrier E_a_ increases from 0.17 eV in ZnSO_4_ electrolyte to 0.24 eV in ZnSO_4_+BTA electrolyte, due to the absorption of BTA additives on the Cu substrate. This implies that the diffusion of the Zn^2+^ ions to the surface of the Cu substrate requires higher energy in the ZnSO_4_+BTA electrolyte than in the ZnSO_4_ electrolyte to complete the final reduction process. Because Zn^2+^must overcome a higher diffusion energy barrier in the electrolyte with BTA before reaching the electrode surface, its arrival rate is limited, leading to an overall slowdown of the reduction kinetics. Additionally, we further investigated the Zn^2^^+^ reduction process without considering the influence of H_2_O molecules (Figures S22 and S23). When two BTA molecules were adsorbed on the Cu substrate, the reduction of Zn^2^^+^ ions was also significantly hindered, which further confirms the inhibitory effect of the strong BTA‐Zn^2^^+^ interactions, which is consistent with the trend shown in Figure 3e.

More importantly, we also analyzed the change of Bader charge during the process from the initial state to the final state (Figure 3f). This analysis method can show the charged state of atoms intuitively and then analyze the ability of atoms to gain and lose electrons. The modeling of the Zn^2+^ ion is derived from the description of their apparent charge, but the Bader charge is based on the calculated electron density, and there is no complete electron gain or loss. The Bader charge value reflects the redistribution of spatial electron density rather than a true change in oxidation state, and thus Zn^2+^ in solution appears with a value of about +1. In the ZnSO_4_ electrolyte, Zn^2+^ ions are reduced gradually when approaching the electrode surface, so the Bader charge gradually decreases during the period from the initial state to the final state. In sharp contrast, the Bader charge curve is significantly different in the presence of BTA. At coordinates 1–2, the Bader charge decreases because of the reduction of Zn^2+^ ions near the electrode surface. When Zn^2+^ ions get close to the BTA molecules, strong interactions are formed between BTA molecules and Zn^2+^ ions, and the N atoms of the BTA molecules with strong electronegativity have the effect of electron absorption on Zn^2+^ ions. Consequently, the Bader charge increases significantly in the process of reaction coordinates 2–5. Because the reduction of Zn^2+^ ions must escape from the interaction with BTA, the final state cannot be completed until the reaction coordinate 9 is reached, which fully reveals that the BTA adsorbed on the anode surface directly leads to the decrease of Zn^2+^ ions reduction kinetics.

Based on the above analysis, we can conclude that during the first layer deposition stage, the mild reduction kinetics matched well with the mass transfer rate, allowing Zn^2+^ ions to fully crystallize and form flaky Zn stack (Figure 3g). However, the nucleation overpotential drops sharply when zinc begins to deposit above the seed layer (Figure 3h). The corresponding reduction kinetics have been significantly accelerated. Subsequently, the Zn^2+^ ions’ mass transfer rate is insufficient to meet the demands of accelerated reduction, leading to a concentration gradient (Figure 3i). Considering the fact that the total charge amount for the reduction of Zn ions to metallic Zn is constant [32], and due to the poor driving force for the nucleation(≥1 mA h cm^−2^), as well as insufficient Zn^2+^ ions on the electrode surface, in order to neutralize the same number of electrons, a limited amount of Zn^2+^ ions have to be quickly be reduced to small metallic Zn particles, and then wait for the subsequent Zn^2+^ ions to replenish, resulting in the gradually formation of the pulverized zinc morphology. This explains the reason why the pulverized zinc on top of the seed layer becomes more rampant as plating prevails, and Zn flakes no longer appear.

In a sharp contrast, the electrolyte with BTA additive exhibits a higher overpotential that provides sufficient nucleation driving force. This facilitates well‐matched reduction kinetics and mass transfer rate, enabling a large number of Zn^2+^ ions on the surface of the electrode to fully crystallize. Consequently, a flat and uniform morphology of Zn deposits with a regular hexagonal (002) crystal plane is formed (Figure 3j–l). In comparison, electrolytes without BTA show a dramatically reduced nucleation overpotential, quickly approaching 0 mV. The same trend occurs on Cu/Zn/Ti/Stainless steel substrates (Figure S24). Therefore, the BTA molecules effectively maintain the nucleation overpotentials during the plating stage, thus effectively preventing the Zn pulverization phenomenon.

### 2.4 The Relation Between the Pulverized Zn and Irreversible Capacity Loss in the Lean Zn Electrode During Cycling

**FIGURE 4:**
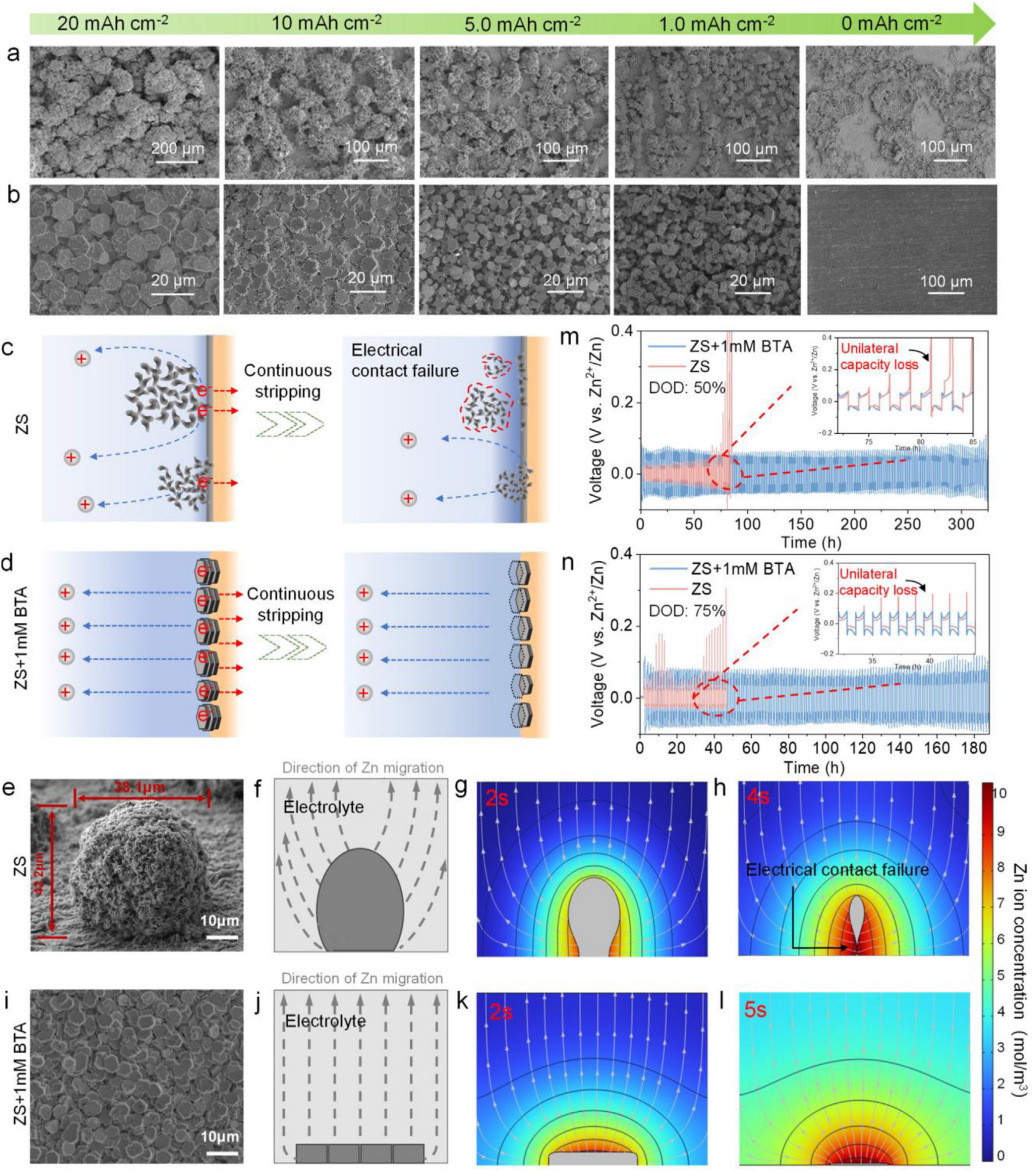
The characteristic of Zn stripping behavior in the ZnSO_4_ and ZnSO_4_+BTA electrolytes. SEM of the lean Zn electrode after different capacities of Zn gradually stripping in (a) ZnSO_4_ and (b) ZnSO_4_+BTA electrolytes. The values on the arrow represent the remaining Zn capacity left on the lean Zn electrode during stripping. Schematic diagram of Zn stripping behaviors in (c) ZnSO_4_ and (d) ZnSO_4_+BTA. (e) Cross‐sectional SEM image on the representative pulverized Zn particle deposited on the Cu substrate in ZnSO_4_ electrolyte. (f) Simulation cell geometry for the Zn stripping behavior on the Cu substrate by mimicking the corresponding pulverized Zn deposition feature in ZnSO_4_ electrolyte. (g, h) Simulations of Zn ion flux distribution, Zn stripping behavior and amount of inactive Zn residue in the ZnSO_4_ electrolyte. (i) SEM image on the representative granular Zn particle deposited on the Cu substrate in ZnSO_4_+BTA electrolyte. (j) Simulation cell geometry for the Zn stripping behavior on the Cu substrate by mimicking the corresponding granular Zn deposition feature in the ZnSO_4_+BTA electrolyte. (k, l) Simulations of Zn ion flux distribution, Zn stripping behavior in the ZnSO_4_+BTA. The symmetrical cells based on lean Zn electrode in ZnSO_4_ and ZnSO_4_+BTA electrolytes at (m) 1 mA cm^−2^ and 1 mA h cm^−2^, (n)2 mA cm^−2^ and 1.5 mA h cm^−2^.

For conventional Zn foil anodes with excessive Zn, the short circuit induced by the rampantly growing Zn dendrites is the main reason for cell failures. However, for the lean Zn anodes, the failure mechanism is different. The morphology of the pulverized Zn bud directly affects the following Zn stripping process. We have clearly observed a significant difference in the zinc stripping morphology between the lean Zn electrode with and without BTA additive (Figure 4a–d). When used as lean‐zinc electrodes, this pulverized zinc morphology is apt to cause contact failure during stripping, finally resulting in cell failure (Figure 4a,c). In contrast, in the ZnSO_4_+BTA electrolyte, uniform and fine zinc particles on the Cu substrate were formed, which ultimately leads to a flat stripping morphology. Therefore, the contact failure does not occur during stripping in the ZnSO_4_+1 mm BTA electrolyte (Figure 4b,d).

To clearly disclose the relation between the pulverized Zn morphology and the contact failure, we further modeled the stripping behavior of Zn@Cu composite anodes under different deposition morphology configurations by COMSOL Multiphysics software. Based on these morphology characterization results (Figure 4e), we created a simulation cell containing one vertically‐standing Zn bud (Figure 4f) on the Cu current collector by simulating the pulverized Zn cluster morphology in the pure ZnSO_4_ electrolyte, and also constructed a dumpy Zn particle on the Cu foil by simulating the platelet‐shaped Zn deposition morphology in the ZnSO_4_+BTA electrolyte. As shown in Figure 4g, when stripping begins, the Zn at the bottom of the mossy morphology is closer to the current collector, resulting in lower electronic resistance, and a shorter electron transfer path way, leading to faster oxidation (Zn→Zn^2+^) into the pure ZnSO_4_ electrolyte. Therefore, during the stripping process, there is a higher stripping current density and Zn^2+^ ion concentration at the bottom side as observed in Figure 4h. This bottom‐preferred stripping behavior leads to the isolated Zn^0^ metal at the top side when the bottom Zn is fully stripped away. In contrast, in the ZnSO_4_+BTA electrolyte, the formation of dense and fine Zn particles leads to a low deposition height difference (Figure 4i,j). Thus, the top and the bottom of the particles have similar stripping speeds, which can be demonstrated by the smaller concentration gradient near the zinc particles in the COMSOL images (Figure 4k,l). Therefore, the inactive Zn° caused by electrical contact failure of the lean Zn anode during stripping has been significantly suppressed in the ZnSO_4_+BTA electrolyte.

Stable plating and stripping behaviors are key to reducing Zn loss for lean Zn electrode during cycling. Coulombic efficiency (CE) is a crucial parameter to evaluate the reversibility of Zn plating/stripping. As depicted in Figures S25 and S26, the Zn|Cu asymmetric cell with ZnSO_4_+1 mm BTA electrolyte displayed stable plating/stripping CE of 99.2% at 1.0 mA cm^−2^–0.5 mA h cm^−2^ for 1150 cycles. In sharp contrast, a short circuit occurred after only 166 cycles in ZnSO_4_ electrolyte. Additionally, we performed cycling tests on high current Zn plating/stripping. Figure S27 respectively verify the CE of Zn|Cu asymmetric cells at 5.0 mAcm^−2^–1.0 mA h cm^−2^ and 10 mA cm^−2^–1.0 mA h cm^−2^, which are as high as 99.6% and 99.2% for 1250 cycles and 1450 cycles. On the contrary, Zn|Cu asymmetric cells without BTA have a much shorter cycle life and are accompanied by larger voltage fluctuations. This fully confirms the role of BTA in promoting favorable Zn plating/stripping morphology and maintaining interface stability for the lean Zn electrodes.

Moreover, the electrochemical performance of the lean Zn electrodes further verified the impact of BTA in suppressing the exhausting speed of Zn. In Figure S28, we pre‐deposited metallic Zn at a capacity of 2 mA h cm^−2^ on each Cu foils as lean‐Zn electrodes, and assembled the Zn@Cu||Zn@Cu symmetrical cells. It is clear that no matter if cycling was proceeded at 50% DOD or 75% DOD (Figure 4m,n), the cells with the bare ZnSO_4_ electrolyte showed a typical voltage profile reflecting severe capacity loss accompanied with one side substantial increase in voltage polarization and inability to attain the applied plating capacity (Figure S29). By contrast, the lean Zn symmetrical cells with the BTA additives maintained a stable cycling performance and do not show significant capacity loss. Furthermore, to fully identify the effectiveness of the BTA additive, we also used 100 µm zinc foil anodes. The Zn||Zn symmetric cells with the ZnSO_4_+BTA electrolyte achieved an ultralong cycle life (over 1450 h) at the current density of 2 mA cm^−2^ with a capacity limitation of 1 mA h cm^−2^ (Figure S30).

### 2.5 The Impact of BTA Additives on the EDL Characteristics of the Lean Zn Electrode

The electric double layer (EDL) is formed due to the selective adsorption of certain ions from the electrolyte onto the electrode interface and its characteristics determine the electrochemical behaviors at the electrode/electrolyte interface [33]. In the conventional ZnSO_4_ electrolyte (Figure S31), [Zn(H_2_O)_6_]^2+^ anions can only approach the outer Helmholtz layer (OHL), large amounts of hydrophilic SO_4_^2−^ and free water solvent molecules preferentially occupy the inner Helmholtz layer (IHP) because of their less solvated structure and faster diffusion kinetics in comparison to the desolvated Zn ions, which cause large amounts of Zn^2+^ ions’ active sites being occupied on the electrode and result in an unstable Zn interface. In sight of this situation, the influence of the BTA additive on the EDL structure of Zn anode is also an important parameter for evaluating the stability of the lean Zn anodes. We initially conducted cyclic voltammetry (CV) tests of Zn@Cu symmetric cells with ZnSO_4_ and ZnSO_4_+BTA electrolytes at different scanning rates (Figure S32). As shown in Figure S33, the interface capacitance of the cells’ electrodes with ZnSO_4_+BTA (0.12 mF cm^−2^) is much lower than that of ZnSO_4_ electrolyte (0.31mF cm^−2^), mainly ascribed to the large‐sized BTA molecules squeeze out H_2_O molecules and absorb on the electrode interface, which increase the thickness of EDL and modify the original water‐rich surface of the IHP [34].

MD simulations were used to investigate the electric double layer (EDL) structure on the Cu substrates under −1.5 V potentials (deposition state) with/without BTA additive (Figure S34). In snapshots of the surface and cross‐sectional images of the EDL structure on the Cu anodes with a negative potential in the ZnSO_4_ electrolyte, a large number of H_2_O molecules, and a small number of Zn^2+^ and SO_4_^2−^ are present in the inner Helmholtz layer. In contrast, after the addition of BTA to the ZnSO_4_ electrolyte, a large number of BTA molecules entered the EDL to reshape its structure, which causes numerous H_2_O molecules to be replaced. Through the normalized density profiles of the EDL on the negative Cu anodes in ZnSO_4_ electrolyte (Figure S35a), it can be more intuitively found that the inner layer contains a considerable number of H_2_O molecules, but this value is significantly reduced to half after adding BTA into the ZnSO_4_ electrolyte (Figure S35b), confirming that BTA can reduce the interfacial water within the EDL. Furthermore, we characterize the influence of BTA on the surface chemistry of the Cu substrate, the XPS of fresh Cu foil and Cu foil extracted from ZnSO_4_+BTA electrolyte were conducted. As shown in the Figure S36c. No obvious N 1s signal was detected on the surface of fresh Cu substrate, while a significant nitrogen peak appearing at 398.1 eV was detected on the Cu substrate exposed to the electrolyte with BTA, which can be attributed to the N 1s of BTA molecules adsorbed on the Cu surface [35], this signifies the formation of N─Cu coordination bonds between BTA and Cu substrate. In addition, the Cu 2p1/2 and Cu 2p3/2 peaks in spectra of the bare Cu foil appear at 952.5 and 932.5 eV (Figure S36d), respectively, both the Cu 2p1/2 (953 eV) and Cu 2p3/2 (933 eV) peaks of the BTA‐Cu foil shift toward higher binding energy, confirming the chemisorption of BTA molecules on the surface of the Cu foil. Based on this above analysis, we can speculate that the chemisorption of BTA molecules on the IHP isolate water molecules from the anode surface to inhibit corrosion and reduce the EDL repulsive force of Zn^2+^ ions. To prove the above expectation, linear sweep voltammetric (LSV) measurements were carried out to evaluate the anticorrosion ability of the Zn@Cu composite anode in ZnSO_4_ electrolyte with and without BTA additive. As shown in Figure S37a, the corrosion current (I_corr_) of the electrode in these electrolytes dropped from 2.160 to 0.101 mA cm^−2^ after adding BTA, indicating that the BTA‐containing EDL exerted an inhibitory effect on the corrosion reaction. In addition, the onset of hydrogen evolution potential decreases from −0.013 to −0.189 V after the addition of BTA (Figure S37b), further demonstrating the successful suppression of the water‐induced HER. In situ differential electrochemical mass spectrometry (DEMS) was used to monitor gas evolution by HER, in contrast, the ZnSO_4_ electrolyte with BTA demonstrated optimal effectiveness in suppressing side reactions, minimizing hydrogen evolution (Figure S37c).

It should be noted that regulating the original [Zn(H_2_O)_6_]^2+^ solvation environment is not the main role of BTA. As depicted in Figure S38a, Nuclear magnetic resonance (1H NMR) spectra showed that the ^1^H chemical shift of the ZnSO_4_ electrolyte with 1 mm BTA slightly decreased to 4.7344 ppm, exhibiting a very slight chemical shift of only 0.0004 ppm compared with the pure ZnSO_4_ electrolyte. Even with a high concentration of BTA (5 mm), no significant chemical shift was detected. Moreover, the ^67^Zn chemical shift in parallel NMR measurements is also invariant to the presence of BTA (Figure S38b). These results clearly prove that the relatively small amount of BTA additive is ineffective in reducing the number of the bound water molecules in the original [Zn(H_2_O)_6_]^2+^ solvation structure [36]. Overall, the main reason for inhibiting the reactivity of H_2_O and improving Zn stability is through the reconstruction of the EDL by BTA molecules, as the aromatic ring of BTA provides a hydrophobic domain that repels water, while the nitrogen atoms (with lone pairs) coordinate strongly to the Cu surface. This interaction is more stable than hydrogen bonding and may explain the observed water corrosion inhibition.

### 2.6 The Influence of BTA on the Static Capacity Loss of the Lean Zn Electrode During Calendar Life

For Zn foil anode, only chemical corrosion occurs through direct charge transfer between the Zn metal and the electrolyte during rest (Figure S39a), leading to irreversible electrolyte decomposition on the surface of the Zn metal. While as for the lean Zn electrode (Zn@Cu), the static capacity loss caused by chemical corrosion, and galvanic corrosion during calendar life. Galvanic corrosion (Figure S39b) is caused by the direct potential difference between the Zn deposits and the Cu foil (current collector), which results in electrons transfer from the zinc metal to the Cu foil. Ultimately, this will lead to the loss of active Zn metal and result in HER on the surface of the Cu foil [37, 38].

To enhance both the cycling performance and calendar stability of lean zinc anodes, various artificial interfacial layers, including ZnO [39], ZnS [40], and ZnF_2_ [41], were employed due to their effectiveness in mitigating hydrogen evolution and suppressing parasitic side reactions on bare zinc foil. In addition, electrolyte additives like TPPS [42] and DMSO [43], which can participate in the solvation structure of Zn^2^^+^ ions and reduce water activity, were incorporated. These strategies were systematically explored to evaluate their impact on mitigating the static capacity loss in lean Zn anodes configured with a zinc/copper current collector structure. It was found that the static capacity loss within 24 h was serious using those strategies (Figure S40). Therefore, the interface hydrogen evolution and passive side reactions caused by the thermodynamic instability of zinc, could be not the main cause of the static capacity loss in lean Zn anode.

**FIGURE 5:**
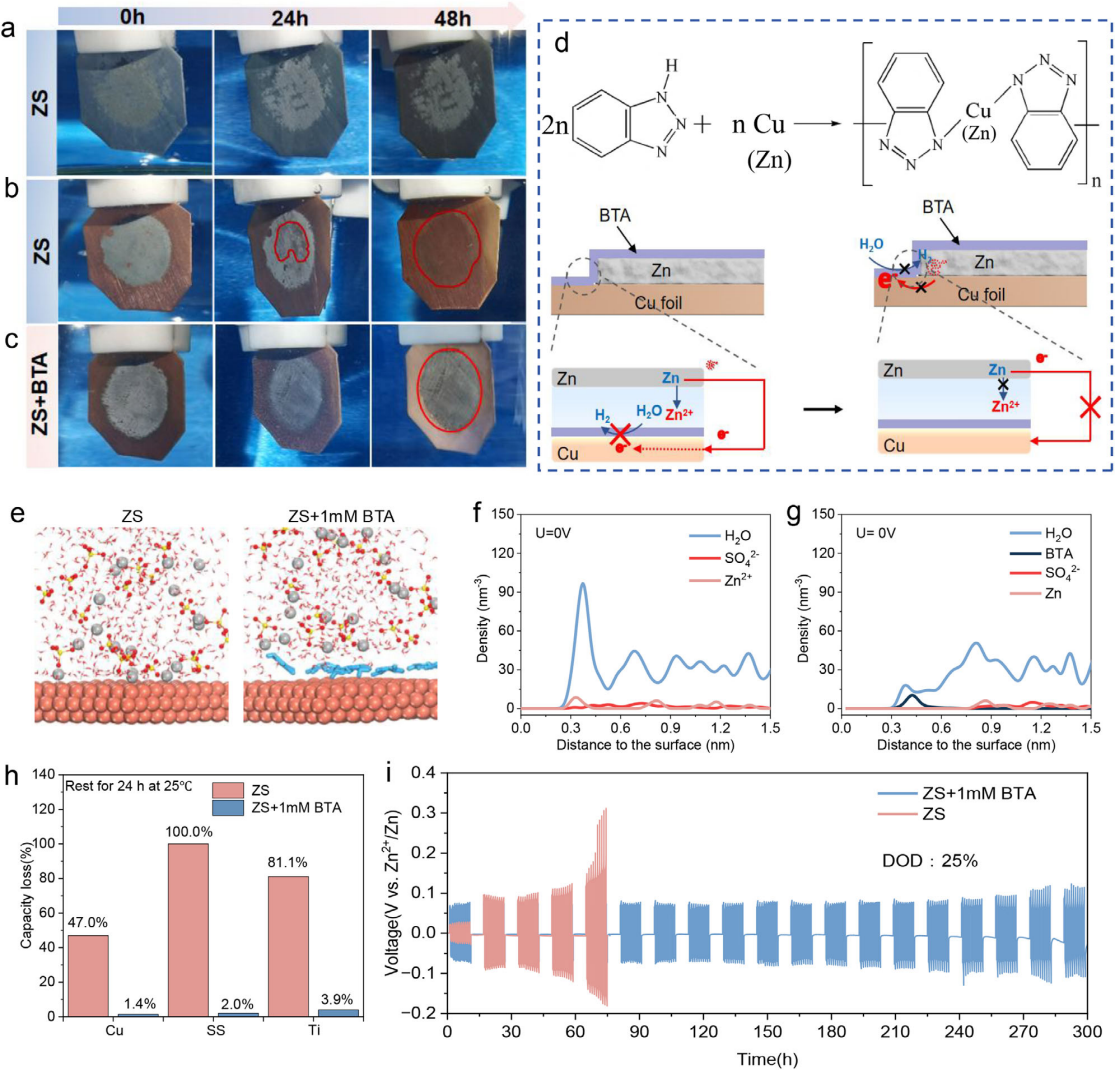
The static capacity loss evaluation of lean Zn anode. (a) Photographs of Zn@Zn electrodes after being immersed in ZnSO_4_ electrolytes. (b) Photographs of lean Zn electrode after being immersed in ZnSO_4_ electrolytes. (c) Photographs of the lean Zn electrode after being immersed in ZnSO_4_+BTA electrolytes. (d) Illustration the suppression of galvanic corrosion on the lean Zn electrode after being immersed in ZnSO_4_+BTA electrolyte. (e) M1 geometry after MD simulations without external voltage U = 0 V. M1: ZnSO_4_/water/Cu substrate. H_2_O is shown as thin sticks. O, H, S, Zn, and Cu are shown as red, white, yellow, gray, and red colors. M2 geometry after MD simulations with a copper anode (U = 0 V). M2: ZnSO_4_+BTA/water/Cu substrate. BTA shown as blue sticks. Density profiles as a function of distance from the Cu anode (U = 0 V) for the SO_4_^2−^, H_2_O, and Zn^2+^ in (f) ZnSO_4_ electrolyte and (g) ZnSO_4_+BTA electrolyte. (h) Static capacity loss of lean Zn electrode based on different substrate (Cu, SS, and Ti) over 24 h in ZnSO_4_ and ZnSO_4_+BTA electrolytes. (i) Shelving‐recovery performance of Zn@Cu||Zn@Cu symmetric cells (1 mA, 0.5 mA h cm^−2^).

To further identify the main cause of the static capacity loss in the lean Zn anode, we pre‐deposited 0.5 mA h cm^−2^ Zn onto Zn foil current collector and immersed them in the 2 m ZnSO_4_ electrolyte to mimic the influence of the chemical corrosion process (Figure 5a). In parallel, we also pre‐deposited 0.5 mA h cm^−2^ Zn onto Cu foil current collectors and immersed them in the 2 m ZnSO_4_ electrolyte with and without BTA to evaluate BTA's influence on the galvanic corrosion and chemical corrosion coexisting conditions (Figure 5b,c). Notably, both the appearance and the Zn deposits’ color on the Zn foils remained unchanged after 48 h of immersion, because there is no potential difference between the Zn deposits and the Zn foil current collectors. Consequently, galvanic corrosion does not occur. On the other hand, the 0.5 mA h cm^−2^ Zn deposits on the Cu foils immersed in the 2 m ZnSO_4_ electrolyte were completely dissolved (Figure 5b), this demonstrated that the galvanic corrosion induces the contact loss between heterogeneous metals (the Zn deposits and Cu substrate), which is significantly contribute to the active Zn loss for the lean Zn anode. Meanwhile, we also noticed that the surface area and color of the Zn deposits on the Cu foil that was immersed in the electrolyte with BTA remained unchanged (Figure 5c), proving that BTA could effectively inhibit galvanic corrosion and chemical corrosion.

We employed a zero‐resistance ammeter (ZRA), a typical technique to measure the galvanic coupling current between two dissimilar electrodes [44, 45] (Figure S41), to quantify the BTA's contribution to the suppression of galvanic corrosion, Cu and Zn foils spaced by a separator were placed in a cell with pure ZnSO_4_ and ZnSO_4_+BTA electrolyte and were externally connected to ensure that the potential differences were consistent. This process mimics galvanic corrosion by separating the anodic and cathodic processes, and then records the electrons passing through, which measures the galvanic corrosion current [37]. As shown in Figure S42, the overall galvanic corrosion current of Zn|Cu cell containing the ZnSO_4_+BTA was much lower than that in a pure ZnSO_4_ electrolyte with a smaller initial corrosion current and decays to only 7 µA cm^−2^ after about 800 min of experiments. Based on this result, it is clear that due to the specific chemisorption ability of BTA on Zn and Cu, an insulating layer can be formed on the Zn@Cu electrode interface, which can effectively block the electron transfer between Zn─Cu galvanic couples existing with the lean Zn anodes which are explored herein (Figure 5d).

Figure 5e shows results of MD simulations that demonstrate the contribution of BTA toward suppression of galvanic corrosion in ZnSO_4_ electrolyte on Cu (111) substrate occurring upon rest. It was found that during the static resting process, BTA molecules accumulated abundantly within the inner layer of the EDL, significantly reducing the number of H_2_O molecules adsorbed on the anode surface (Figure 5f,g). Although the above calculations were performed on the widely used Cu (111), reflecting the major surface chemistry of real polycrystalline Cu substrates, the qualitative trends identified here help us to clarify that BTA can physically hinder electron transfer between the Cu substrate and H_2_O molecules, thereby blocking the galvanic corrosion pathway and effectively suppressing the static capacity loss.

We further quantified the static capacity loss of the lean Zn electrodes prepared on different current collectors (e.g., Cu, Carbon Paper, Ti, Graphite foil, Al, Ni, and Stainless steel) in additive‐free ZnSO_4_ electrolyte within 24 h. Severe capacity degradation was observed for all types of anodes, with more than 40% capacity loss detected in each case (Figures S43–S45). Notably, lean Zn electrodes on Ni and stainless steel substrates suffered complete (100%) capacity loss accompanied by pronounced voltage fluctuations during the rest process (Figure S44). These results demonstrate that calendar aging poses a critical challenge for the practical development of secondary Zn batteries, regardless of the conductive current collectors employed. In contrast, the introduction of BTA additive remarkably mitigated the static capacity loss (24 h) of the lean Zn electrodes on the Cu, Ti, and stainless steel substrates (Figure 5h), reducing the losses to only 1.4%, 2.0%, and 3.9%, respectively.

We further investigated the inhibitory effect of different BTA concentrations on the static corrosion of lean Zn anodes within 24 h by Tafel polarization curves and electrochemical impedance spectroscopy (EIS) analyses [46, 47]. As shown in Figure S46a and Table S1, when the BTA concentration is 0.1 and 0.5 mm, the static corrosion inhibition effect is negligible, and the corrosion inhibition efficiency η is only 4.0% and 7.0%, respectively. Remarkably, as the BTA concentration increases to 1 or 1.5 mm, the corrosion inhibition efficiency η increases significantly, to 97.0% and 99.3%, respectively. Furthermore, the EIS analyses (Figure S46b) reveal that the impedance of the electrodes increases significantly with increasing BTA concentration, confirming that the enhanced corrosion inhibition resistance originates from the progressive adsorption of BTA molecules onto the anode surface, which effectively hinders charge transfer and suppresses electrochemical corrosion.

Finally, to confirm the practical applicability of BTA for lean Zn anodes, symmetric cells comprising Zn@Cu electrodes were tested in prolonged cycling experiments that included shelving‐recovery steps. Such steps included rest periods (at OCV) during which self‐discharge occurred because of the above‐mentioned Zn galvanic corrosion. Then, the cells were returned to repeated cycling tests. Symmetric cells with lean Zn electrodes (2 mA h Zn deposits on the Cu substrate) in ZnSO_4_+BTA electrolyte were cycled at different depths of discharge (DOD). 25% and 50% DOD were evaluated. As for the 25% DOD test in Figure 5i, the repeated steps of cycling and rest were 10 and 6 h respectively. After the cells in pure ZnSO_4_ electrolyte were shelved for the first 6 h, there was a significant increase in their voltage hysteresis upon cycling. Whether during charging or discharging, the voltage increases symmetrically, showing bilateral polarization (Figure 5i and Figure S47a), and implying the accumulation of byproducts during rest and hindered ion transport channels. Afterward, for the BTA free cells after 65 h of cycling/rest experiments, we see asymmetry in the voltage curve that we term unilateral voltage polarization. This phenomenon presented in Figure S47b means that one of the electrodes in the cell lost its pre‐deposited zinc and thereby its anodic polarization at constant current requires the use of high voltages, which represents a loss of electrode capacity and finally results in failure. In contrast, at 25%DOD, the cell in ZnSO_4_+BTA electrolyte can run stably for more than 300 h. In addition, when the discharge depth reaches 50%, the repeated steps of cycling and rest were 10 and 12 h respectively, the introduction of BTA can also effectively improve the electrochemical performance in such more demanding experiments as well (Figure S48).

### 2.7 The Evaluation of BTA Effect on the Performance of Zn‐Ion Full Cells With Controlled N/P Ratio

**FIGURE 6:**
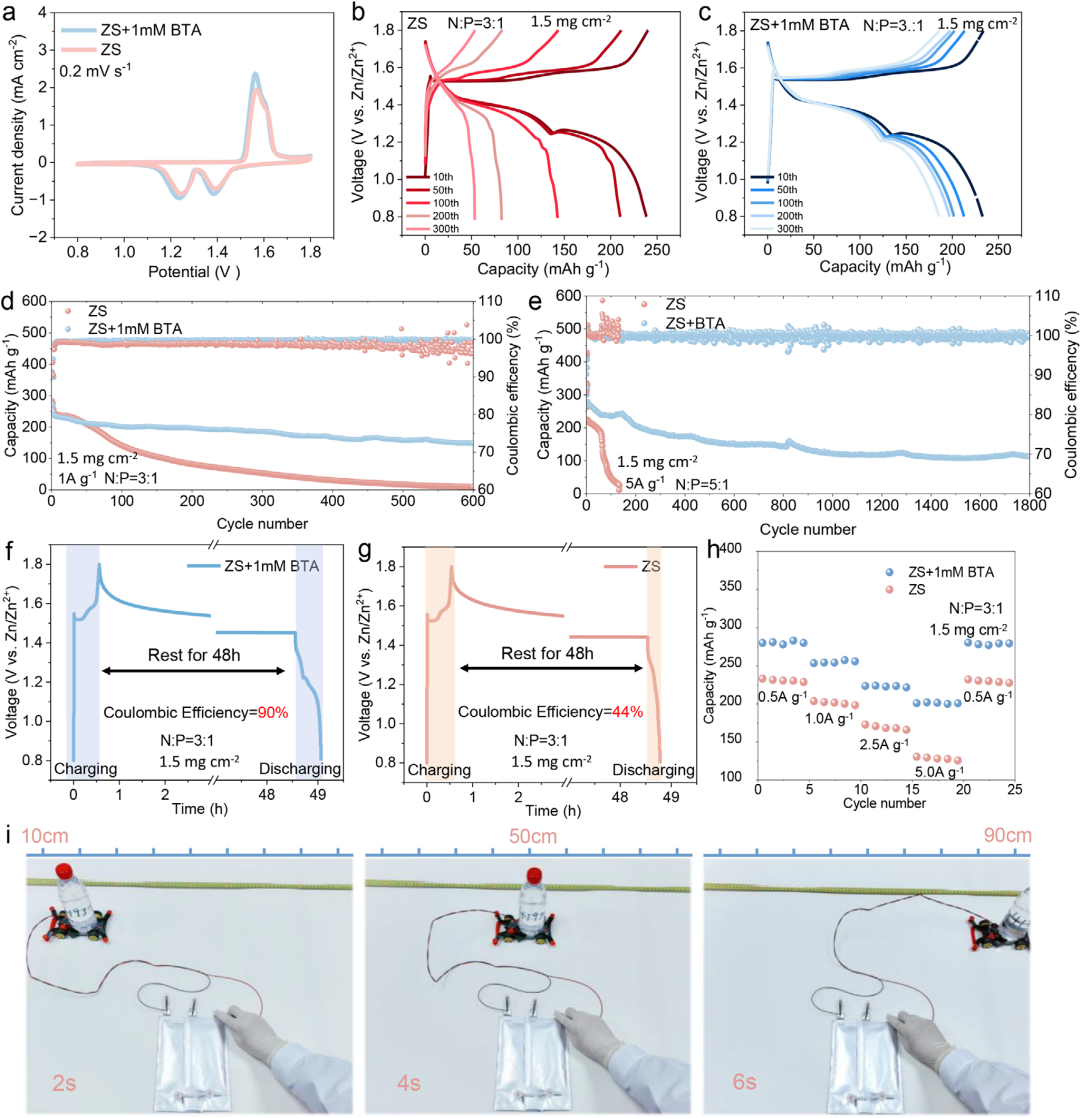
The electrochemical characteristics of lean Zn anode. (a) CV curves of the Zn@Cu||MnO_2_ full cells in ZnSO_4_ electrolyte with/without BTA at 0.2 mV s^−1^. (b, c) The corresponding capacity‐voltage curves for the long‐term cycling performance of different full cells at 1 A g^−1^. (d) Long‐term cycling performance of full cells at 1 A g^−1^. (e) Long‐term cycling performance of full cells at 5 A g^−1^. Self‐discharge performance of Zn@Cu||MnO_2_ full cells in (f) ZnSO_4_+BTA and (g) ZnSO_4_ electrolyte. (h) Rate capacities at different current densities. (i) Images of Zn@Cu||MnO_2_ full cells with BTA additive driving a toy car loaded with 419 g of water.

To demonstrate the performance of the Zn‐ion full cells using ZnSO_4_+BTA electrolyte, MnO_2_ nanofibers were synthesized as cathode materials and the prototype full batteries were assembled (Figure S49). Cyclic voltametric measurements of Zn@Cu||MnO_2_ full cells (lean Zn anode) reflect the behavior of the MnO_2_ cathodes as Zn ions intercalation electrodes. No matter if the cells contained BTA, their voltammograms maintained a similar shape and representative peaks at 1.4 and 1.25 V, demonstrating that BTA does not alter the typical redox reaction of the MnO_2_ cathode as Zn ions intercalation electrodes in these full cells (Figure 6a). In addition, we further tested the cycling performance of Zn@Cu||MnO_2_ full cells with a N/P ratio of 3:1 (can be considered as a practical ratio for secondary batteries based on active metal anodes). Voltage profiles of these cells are presented in Figure 6b,c, and representative prolonged cycling tests results are presented in Figure 6d. It is worth noting that the Zn@Cu||MnO_2_ full cells with BTA delivered outstanding electrochemical reversibility for cycling 600 times (157 mA h g^−1^) at a current density of 1 A g^−1^, demonstrating high CE (99.93%) and excellent capacity retention (90.03%), while still maintaining the distinct discharge voltage plateaus after 300 cycles. In turn, the full cells without BTA additive exhibited severe capacity degradation in similar prolonged cycling tests. More encouragingly, when we increased the current density to 5 A g^−1^, Zn@Cu||MnO_2_ full cells with the BTA additive under the N/P ratio of 5 can also keep a stable cycling performance even after 1800 cycles, which far exceeds the poor stability (less than 200 cycles) of BTA free full cells (Figure 6e).

The self‐discharge tests were also employed to reveal the stability of these full cells upon rest at OCV. Specifically, based on the N/P ratio of 3, Zn@Cu||MnO_2_ full cells with BTA additive is charged to 1.8 V followed by a subsequent rest for 48 h, and then discharged to 0.8 V (Figure 6f). These cells could deliver a higher CE of 98% after 48 h of resting compared to BTA free full cells (44%) (Figure 6g). The rate performance tests of these cells with the BTA also outperform the BTA‐free full cells (Figure 6h). In addition, we assembled full cells with Zn‐free anodes (only bare Cu foils) and pre‐zincificated MnO_2_ cathodes. The capacity of such cells (1C, 0.9 mg cm^−2^) with Zn‐free anodes containing BTA‐free ZnSO_4_ electrolyte began to decline sharply after the first few cycles and decayed to zero capacity after 90 cycles only. However, 60% capacity retention has been achieved after 100 cycles in the ZnSO_4_+BTA electrolyte (Figures S50 and S51). As a practical application demonstration, Figure 6i and Videos S1 and S2 show that two 1Ah pouch Zn@Cu||MnO_2_ batteries (10 mg/cm^2^, N:p = 3:1) with BTA additive can successfully drive a toy car loaded with 419 g of water (Figures S52–S54), manifesting good power characteristics of these battery prototypes in pouch cells.

## 3 Conclusion

This work reveals the failure mechanisms of AZIBs with lean Zn anodes for cycling and calendar aging. In traditional ZnSO_4_ electrolyte, zinc metal anodes (Zn, Cu, Ti, and SS substrates) subjected to high areal capacity cycling exhibit a severe zinc pulverization problem which has been long overlooked. The pulverized zinc tends to lose contact with the current collector, leading to a significant loss of active zinc. In this work, we demonstrate rapid Zn nucleation rate produces numerous competing nuclei, hindering the growth of existing ones, and resulting in pulverized Zn deposits. Restricting Zn nucleation kinetics, such as introducing a nucleation inhibitor (e.g., BTA), is essential to allow Zn nuclei to grow into compact microflakes rather than fragile, pulverized structures. With the deposition areal capacity increases, the BTA‐regulated zinc deposits exhibit progressively larger grain sizes, accompanied by a bottom‐up longitudinal growth phenomenon, which benefits for the subsequent uniform and continuous stripping. On the other hand, for calendar aging control, BTA can physically isolate the electron transfer between the Cu substrate and H_2_O molecules, making the overall galvanic corrosion blocked, thus the static capacity loss is suppressed. Our work provides new theories and strategies on the failure mechanism and structure‐performance relationship of the zinc anode with controlled N/P ratio, which is important for the building of high utilization and more practical Zn metal battery.

## Author Contributions

J.X. conceived the idea and took the lead in writing the manuscript. H.L. and Q.J. conducted the main experiments. L.Z. performed synchrotron X‐ray tomography, G.W., D.A., B. S., and E.B. provided critical feedback and helped shape the research, analysis, and manuscript.

## Funding

Jing Xu would like to thank the financial support by the National Natural Science Foundations of China Grant (52002358) and Henan Natural Science Foundation General Project (252300421315).

## Conflicts of Interest

The authors declare no conflicts of interest.

## Supporting information




**Supporting File 1**: advs77731‐sup‐0001‐SuppMat.pdf.

**Supporting File 2**: advs77731‐sup‐0002‐VideoS1.mp4.

**Supporting File 3**: advs77731‐sup‐0003‐VideoS2.mov.

## Data Availability

The data that support the findings of this study are available from the corresponding author upon reasonable request.
