# Supplementary material for: Synergistic Regulation of Deep‐Cycling‐Induced Zn Pulverization and Resting Galvanic Corrosion in Practical Lean Zinc Anodes
Source: Adv Sci (Weinh). 2026 Sep 10:e77731. Online ahead of print. doi: 10.1002/advs.77731 (PMC13562791; doi:10.1002/advs.77731)
Supplement: Supplementary file 1 [file ADVS-9999-e77731-s001.pdf]

Supplementary Information for

Synergistic Regulation of Deep-Cycling-Induced Zn Pulverization and Resting Galvanic Corrosion in Practical Lean Zinc Anodes

Jing Xu[a], Haolin Li[a], Qiyin Jing[a], Lingfei Zhao[b], Elad Ballas[c], Bing Sun[b]*, Doron Aurbach[c]*, Guoxiu Wang[b]**

[a] Research Center of Grid Energy Storage and Battery Application, School of Electrical and Information Engineering, Zhengzhou University, Zhengzhou 450001, China

[b] Centre for Clean Energy Technology, School of Mathematical and Physical Sciences, Faculty of Science, University of Technology Sydney, Ultimo NSW 2007, Australia

[c] Department of Chemistry and Institute of Nanotechnology & Advanced Materials (BINA), Bar-Ilan University, Ramat Gan, Israel

* Corresponding Authors: xu.jing@zzu.edu.cn; bing.sun@uts.edu.au; Doron.Aurbach@biu.ac.il; guoxiu.wang@uts.edu.au;

Experimental Section

The preparation of the electrolytes and lean-zinc anode Cu@Zn. The pure 2 M $ZnSO_4$ electrolyte was prepared using $ZnSO_4·7H_2O$ (Sinopharm, >99.5%) and deionized water. The control electrolytes were prepared by adding different amount (0.1mM, 0.5mM, and 1mM, 1.5mM, respectively) of benzotriazole ($C_6H_5N_3$, Aladdin, 99%) into 2M $ZnSO_4$ electrolyte. The optimized concentration of benzotriazole is 1mM, and the relevant electrolyte was denoted as ZS+1mM BTA electrolyte. **lean-zinc anode:** Firstly, polish the 100μm Cu foil with 1500 grit sandpaper to a bright finish, then immerse it in alcohol for ultrasonic washing for 15 mins. Then take it out and put it into an oven to dry for 30 mins. Finally, zinc with capacities of 1mAh cm^{-2} and 2 mAh cm^{-2} were deposited on Cu foil as lean-zinc anodes with different capacities, which was denoted as Cu@Zn.

Cathode Preparation. The MnO_2 was prepared through a facile hydrothermal method.[1] Firstly, 0.3803 g $MnSO_4·H_2O$ (Aladdin, 99%) was added to 15 ml deionized water to form solution A. 0.237g $KMnO_4$ was dissolved in 15 ml deionized water to form solution B. Secondly, solution B was added to solution A to form solution C, which was stirred for 15 mins, then transferred to a Teflon-lined autoclave and heated at 160°C for 12 h. Finally, after cooling naturally to room temperature, the precipitate obtained was centrifuged, washed with deionized water, and then dried overnight. The prepared MnO_2 was prepared and will be used as a cathode material in the future. To prepare the cathode electrode, MnO_2 material, CNT and Polyvinylidene fluoride (PVDF) with a mass ratio of 4.5:4.5:1 were ground and fully mixed in NMP to prepare the slurry. The slurry was evenly coated on carbon paper (CP) and dried in a vacuum oven at 60 °C for 12 h. Finally, the dried carbon paper was cut into circular electrodes with a diameter of 12 mm.

Preparation of materials involved in other modification measures. The preparation method of ZnSe-$CoSe_2$@NC is provided by the reference literature.[2] The PVDF binder

with a mass ratio of 1:9 to the ZnSe-$CoSe_2$@NC powder was added. The mixture was ground mechanically for 20 mins, then mix thoroughly in NMP to prepare the slurry. After stirring for 12 hours, the slurry was applied uniformly onto the Cu foil. The coated electrode was placed in a vacuum oven and dried at 80 °C for 12 h to remove the NMP solvation. Finally, take out the coated Cu foil and cut it into 12 mm round slices. The ZnO and ZnF_2-coated Cu electrodes were prepared using a similar method. The modified electrolyte (ZS+2wt% TPPS) was prepared by adding 2wt% tetraphenylporphyrin tetrasulfonic acid (TPPS, $C_{44}H_{30}N_4O_{12}S_4$, Maclin, >85%) into 2M $ZnSO_4$ electrolyte. The 3M $Zn(OTf)_2$ electrolyte was prepared using $Zn(OTf)_2$ (Maclin, 98%) and deionized water.

Material Characterization. Nuclear magnetic resonance (NMR) spectra of different aqueous solutions were collected via AVANCE III 400 MHz for illustration of the BTA interaction with Zn^{2+} and H_2O. When preparing the electrolyte, substitute the ordinary ^{1}H-containing solvent (H_2O) with deuterated solvent (D_2O). Carefully and thoroughly rinse all samples for material characterization with deionized water to eliminate any trace of residual salts. X-ray photoelectron spectroscopy (XPS) test of N 1s and Cu 2p at Cu foil were obtained from AXIS Supra; the binding energies were referenced to the C 1s peak at 284.8 eV of the surface adventitious carbon. In a quartz cell, Zn foil and Cu foil are employed as the anode and cathode, respectively, with an effective reaction area of 10 cm^{-2}. Then, deposit 30 mAh cm^{-2} of zinc on Cu foil at 10mA cm^{-2} with/without BTA. Crystalline structures of all of the Zn deposits were analyzed by X-ray diffraction (XRD, Bruker D8 diffractometer) at a scanning mode of 1D, a scanning step of 0.01°, and a scanning rate of 10° min^{-1}. Scanning electron microscopy (SEM, ZEISS Auriga) was applied to investigate the surface morphologies of Zn deposits. For TEM sample preparation, zinc was deposited on Cu foil in ZS and ZS+1mM BTA electrolytes respectively. A total capacity of 30 mAh cm^{-2} was deposited under a constant current of 10 mA cm^{-2}. Then, gently scrape off the deposited zinc and collect the powder. Subsequently, it was ultrasonically dispersed and loaded onto a copper mesh. TEM images were collected firom FEI Tecnai F20 transmission electron

microscope. In situ optical microscope is used to study the deposition behavior of Zn on Cu substrate in different electrolytes at 5mA cm^{-2}.

Electrochemical Measurements. The cycling performances were tested on LAND battery testers using coin cells (CR 2016). Electrochemical measure was performed on Bio-Logic VSP Five-Channel Electrochemical Workstations. Symmetric cells were assembled using positive shells, glass fiber separators, Cu@Zn electrode and negative shells, 70 μL 2 M electrolyte was added. ZnCu half cells were assembled with the Zn electrode (counter electrode, 100μm), Cu (100μm) as the current collector of the working electrode and glass fiber as the separator. The full cells were assembled using Cu@Zn as the anode, MnO_2 on CP as the cathode and a glass fiber as the separator. The nucleation overpotential (NOP) was tested using cyclic voltammetry (CV) measurement by scanning between -0.2 and 0.5V at 0.1 mV s^{-1}, using half cells with the Zn as anode and Cu as the cathode in ZS and ZS+1mM BTA electrolytes. In the Tafel diagram comparing the electrochemical reduction kinetics of Zn^{2+} ions in the two electrolytes, a rotating disk electrode served as the working electrode, an Ag/AgCl electrode as the reference electrode, and a Pt foil as the counter electrode. Measurements were taken at a rotational speed of 3600 rpm to eliminate the effect of mass transfer. [3] The initial voltage for water-induced H_2 evolution of the ZS electrolyte with 1mM BTA (abbreviated as ZS+1mM BTA) and pure ZS electrolyte was measured using the linear sweep voltammetry (LSV) method at a scan rate of 1 mV s^{-1} and a scan range from -0.5 to 1 V with coin cells in which Cu foil is adopted as a working electrode and a Zn plate is adopted as a counter and reference electrode. Tafel tests were performed on Cu@ZnCu@Zn symmetrical cells at a rate of 1 mV s^{-1} in the range of -0.2 to 0.2V to calculate the corrosion current in different electrolytes. Chronoamperometry (CA) tests was measured at overpotentials of -150 mV with ZnCu cell, which aims to compare the diffusion processes during deposition on Cu substrates in different electrolytes. Zero resistance ammetry (ZRA) measures the corrosion rate by setting the potential between ZnCu half cells to 0 V and recording the current passing through the electrode using an electrochemical workstation (Bio-Logic). Coulombic

Efficiency (CE) was measured in half cells using different substrates with a cut-off voltage of 0.5 V vs. Zn^{2+}/Zn. Half cells with aging intervals were aged for 24 hours at an open circuit after the plating step of the tenth cycle.

Synchrotron Micro-Computed Tomography experiment. To determine the inhibitory effect of BTA on the formation of pulverized zinc, a constant areal value of 5mAh cm^{-2} zinc was deposited on Cu foil in ZS and ZS+1mM BTA electrolytes. Then, the two Cu foils deposited with Zn were subjected to ultrasonic cleaning in alcohol, followed by drying in a 60° C oven. Finally, they were cut into 1mm×4mm strips and attached to plastic petri dishes for testing. The synchrotron MCT experiment was carried out at Australia's Nuclear Science and Technology Organisation (ANSTO) with a white beam detector of 39 keV, a magnification of 9X, and an effective pixel size of 0.65 μm.

COMSOL simulation. To clarify the influence of deposition morphology on Zn loss during cycling, a simplified two-dimensional distribution model at the interface between zinc anode and electrolyte was established using COMSOL. In all models, the length of both the cathode and the anode is 60 μm, and the distance between the them is 40 μm. This model mainly simulates the interfacial migration of Zn and the change of ion concentration during stripping. The ellipsoidal model simulates the morphology of pulverized mossy zinc, while the small square stacking model simulates the morphology of finely granular zinc. The Tertiary Current Distribution and Nernst-Planck Equations interface are used to set the initial conditions. The flux of each ion in the electrolyte is calculated using the Nernst-Planck (1) equation.

$$N_i = -D_i \nabla C_i - z_i u_i F c_i \nabla \phi_l$$

Where N_i represents the transfer vector (mol/(m^2·s)), c_i represents the electrolyte concentration(mol/m^3), z_i represents the number of charges carried by the ion, u_i represents the mobility of the charged substance (m^2/(s·J·mole)), F represents the Faraday constant (As/mole), ϕ_l is the electrolyte potential (V). The anode potential is

set to 0.6V, and the cathode potential is set to 0V. The diffusion coefficient D_i is set to 10^{-11} m^2/s. The transfer coefficient of the anode and cathode is set to 0.5.

Computational method

Force field molecular dynamics: In this work, computational simulations were first conducted using Materials Studio under the force field molecular dynamics (MD) scheme. Specifically, the COMPASS III force field was utilized for simulating Cu foil surface, Zn^{2+}, H_2O, SO_4^{2-} and BTA. During the calculations, Zn^{2+} ions and SO_4^{2-} were randomly dissolved in an aqueous solution, initially displaying a typical $[Zn(H_2O)_6]^{2+}$ structure with a Zn-O distance ranging from 2.02 to 2.10 Å. The Cu surface has been modeled using a Cu(111)-p(10×10) slab, on which $ZnSO_4$ solutions have been loaded with or without BTA coverage on the surface. In the simulations, 18 Zn^{2+}/SO_4^{2-} pairs are dissolved in the solution with 513 water molecules and fully optimized with energy converged to 10^{-4} kcal/mol and force converged to 0.005 kcal/mol/Å, followed by force-field molecular dynamics (MD) simulations. Specifically, MD simulations were conducted with a time step Δt=1 fs, running for 5,000 ps. The last 1,000 ps has been collected for further analysis. The simulation was performed with NVT ensemble with T= 298.0 K with a Nose themostate. For the BTA-modified surface, since the adsorption geometry of single BTA has been initially tested by first principle calculations, this calculation has been utilized as a reference for randomly loading six BTA molecules on Cu(111). This simulates the restructuring effect of the EDLs upon the addition of BTA. Same optimizations and MD simulations were further carried out to compare with non-modified Cu(111), with a vacuum space (~20 Å) above the solution.

Density functional theory calculations: The diffusion of Zn^{2+} from the solution to Cu(111) experiences a reduction process, first principle calculations were performed using spin-polarized density functional theory (DFT), using the Vienna ab initio simulation package (VASP) with the Perdew, Burke and Ernzerhof (PBE) exchange-correlation potential within the generalized gradient approximation. The projector augmented wave (PAW) pseudopotential was used to describe core electrons. The

valence/outer-core electrons that are included in the self-consistent-field calculations are listed inter parentheses for each atom: Zn($3d^{10}$ $4s^2$), Cu($3d^9$ $4s^2$), O($2s^2$ $2p^4$), N($2s^3$ $2p^2$), C($2s^2$ $2p^2$), and H($1s^1$). To simulate the adsorption of BTA on the electrode surface and its alteration of the EDL structure, a model with Cu(111)-p(4×4) with four Cu-layers has been used in DFT calculations, on which single and double BTA molecules were tested. For the diffusion and reduction process of Zn^{2+} in ZS electrolyte, energy profile and Bader charge changes have been observed through inserting 7 points between initial state (IS, Zn^{2+} being in the solution and away from the surface) and final state (FS, fully adsorbed on Cu(111)). In contrast, due to BTA's effect of reducing reduction kinetics, it is necessary to insert two additional points to fully capture the entire process from IS to FS of energy and Bader charge changes. For all optimization calculations, the cutoff energy was set to 420 eV and K-space was sampled by Gamma point, with bottom two layers fixed during the optimization. The convergence criteria for energy and force were set to 10^{-4} eV and 0.03 eV $Å^{-1}$, respectively. The van der Waals interaction has been considered using the Grimme dispersion scheme. Charge transfer was analysed based on the Bader population.

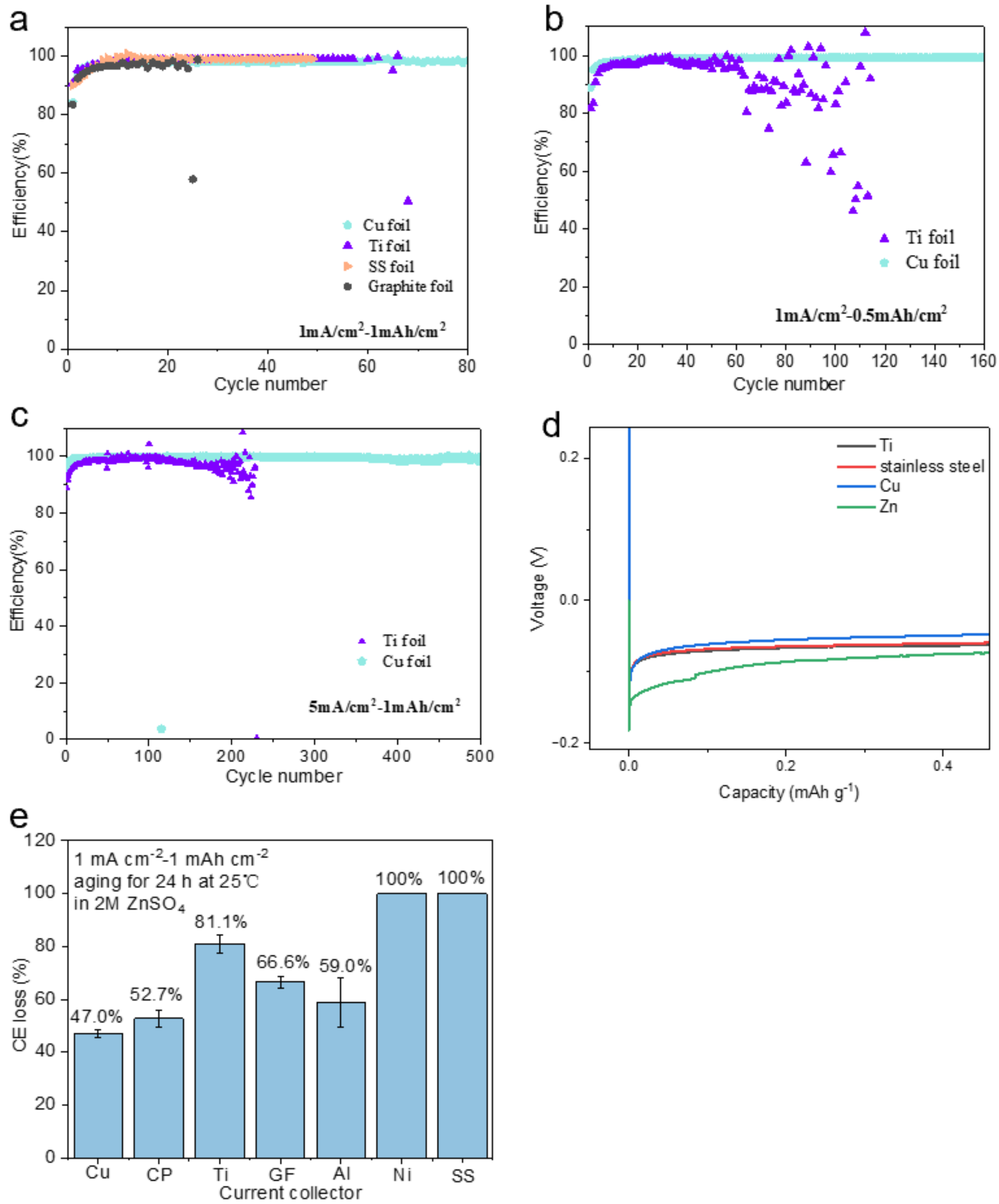

Figure S1 (a-c) Effects of different current collectors on the coulombic efficiency testing of zinc half-cells (d) The nucleation overpotential on the different substrates in 2 M $ZnSO_4$ electrolyte (e) The static capacity loss of lean Zn anode based on different current collectors

Note S1

When using pure zinc foil of the nearly same mass as the copper current collector + low-zinc anode, it may raise the following issues:

Challenge 1: Inhomogeneous Zinc Deposition/Dissolution

Due to the inherent surface roughness and unevenness of the bare zinc foil, zinc ions tend to preferentially deposit at protrusions, leading to rapid dendrite formation and perforation. Dendrites can pierce the separator, causing short circuits, and may detach to become "dead zinc". In contrast, as for lean Zinc anodes, the copper current collector exhibits good lattice matching with zinc, providing abundant nucleation sites on its surface [4]. This can promote uniform zinc deposition and growth compared with bare Zn foil, reducing local current density and suppressing dendrite formation.

Challenge 2: Mechanical Stability and Contact Failure

When the entire pure zinc foil serves as the active material, it undergoes significant thickness expansion and contraction during repeated dissolution-deposition cycles. This macroscopic deformation generates substantial stress, leading to localized foil fracture, wrinkling, and loss of electrode integrity [5]. Once electrical contact is lost in certain areas, they become "dead zinc" (**Figure S2a**), resulting in rapid capacity fade. In contrast, zinc-lean anodes experience much smaller changes (**Figure S2b-d**).

Challenge 3: Current Distribution

The electrical conductivity of zinc (~16.6 nΩ·m) is far lower than that of copper (~1.68 nΩ·m). As zinc dissolves from the Zn foil surface, the current path lengthens and internal resistance increases, leading to highly uneven current distribution. Current tends to concentrate at the edges or tips of the pure zinc foil, causing localized over-dissolution or excessive deposition, which accelerates failure in these areas while leaving the central regions underutilized.

Challenge 4: Limited Cycle Life and Depth

The theoretical energy density of the full cell with pure zinc foil may be misleadingly high. Due to the issues above, the practical utilization of zinc can be very low (possibly limited to the surface), resulting in a reversible capacity far below the theoretical value [6]. Moreover, to mitigate short-circuit risks, thicker and heavier separators might be required. To achieve acceptable cycle life, a large negative/positive capacity ratio (N/P ratio)-i.e., a significant excess of zinc is often necessary. However, this excess zinc counteracts the energy density advantage. The reviewer's "equal mass" condition may also imply a potentially low N/P ratio (the total zinc foil may not be in excess), which would further deteriorate the cycle performance of cells employing pure zinc foil anodes.

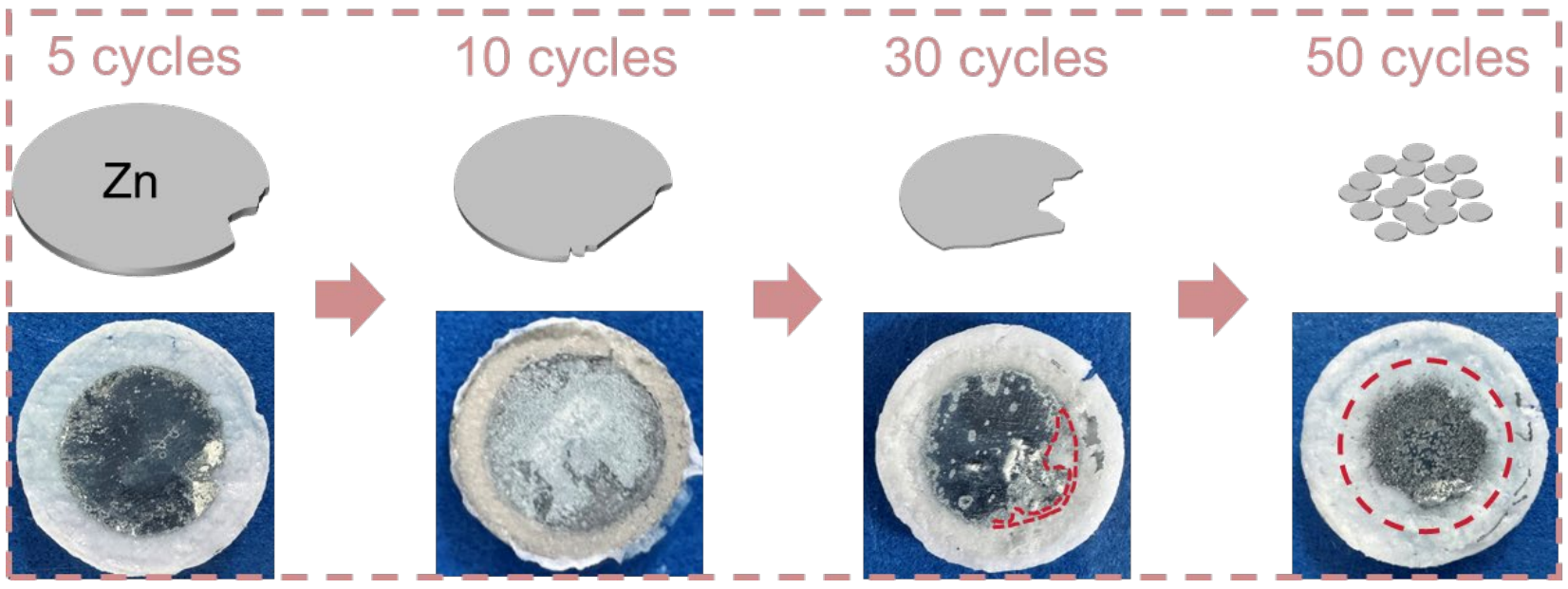

Figure S2a The images of Zn foil with the thickness of 20 μm after cycles

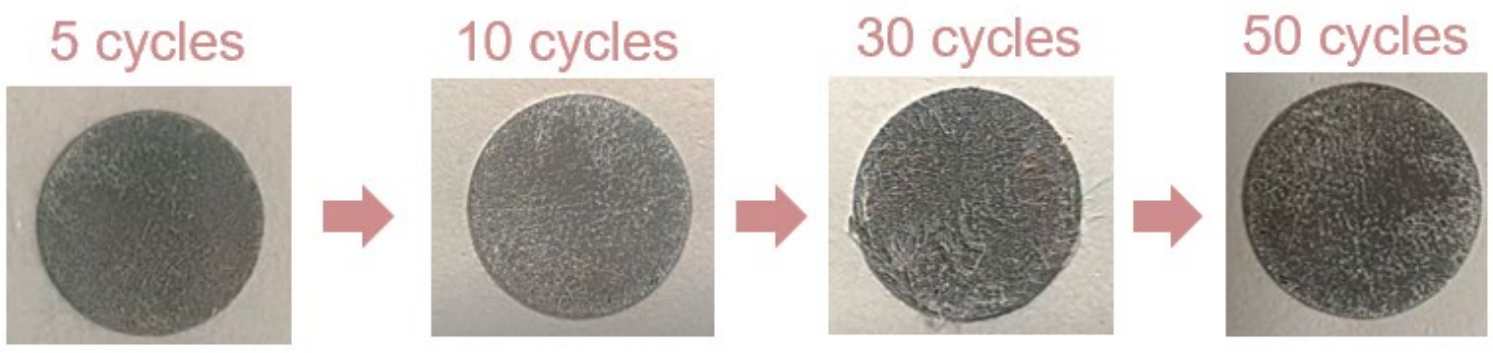

Figure S2b The images of Zn@Cu electrode (10 μm Cu substrate + 12 um Zn) after cycles

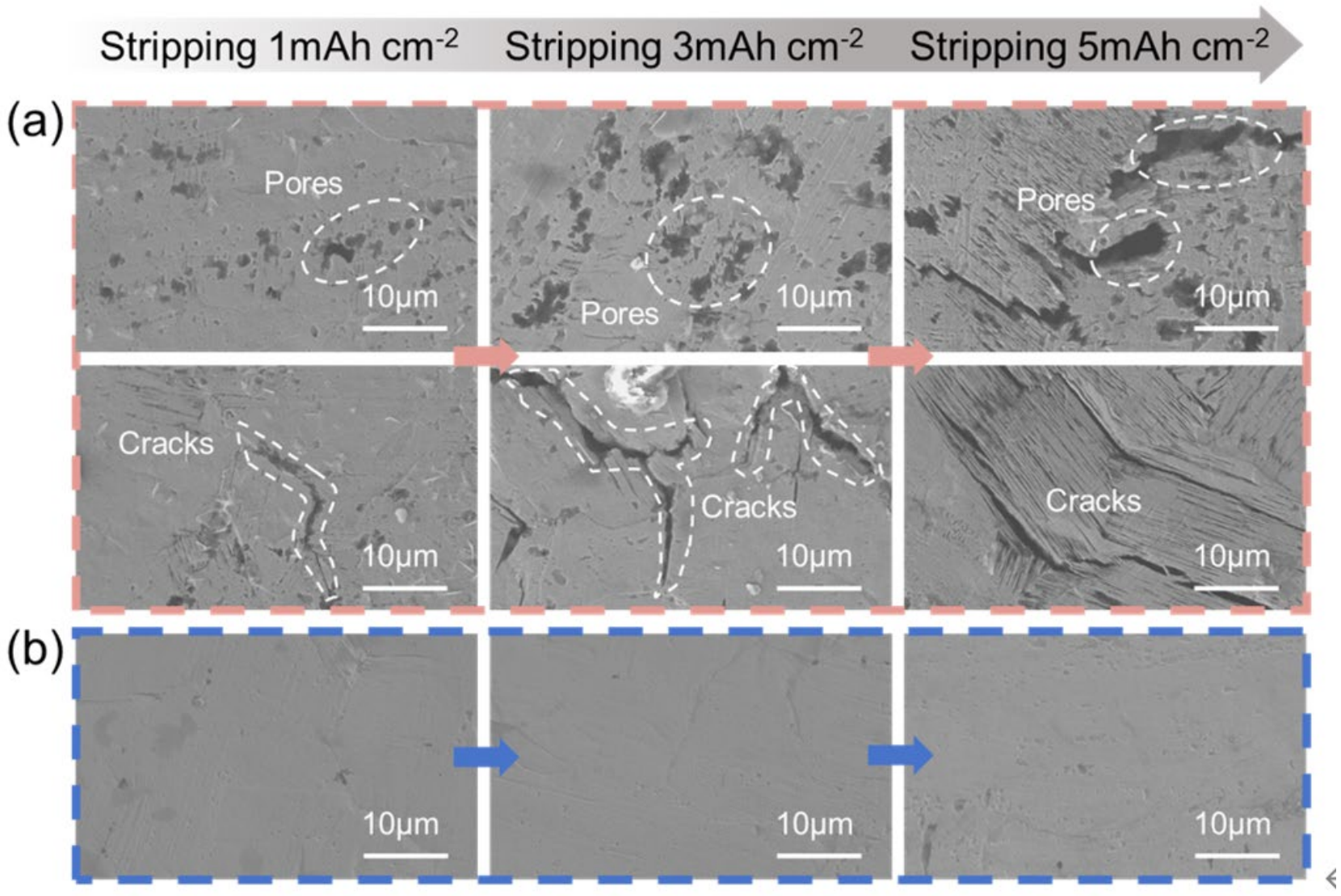

Figure S2c (a) SEM images of zinc foil electrode (20 μm) after different stripping capacities (b) SEM images of lean Zn@Cu electrode (10μm Cu substrate + 12 μm Zn deposits) after different stripping capacities.

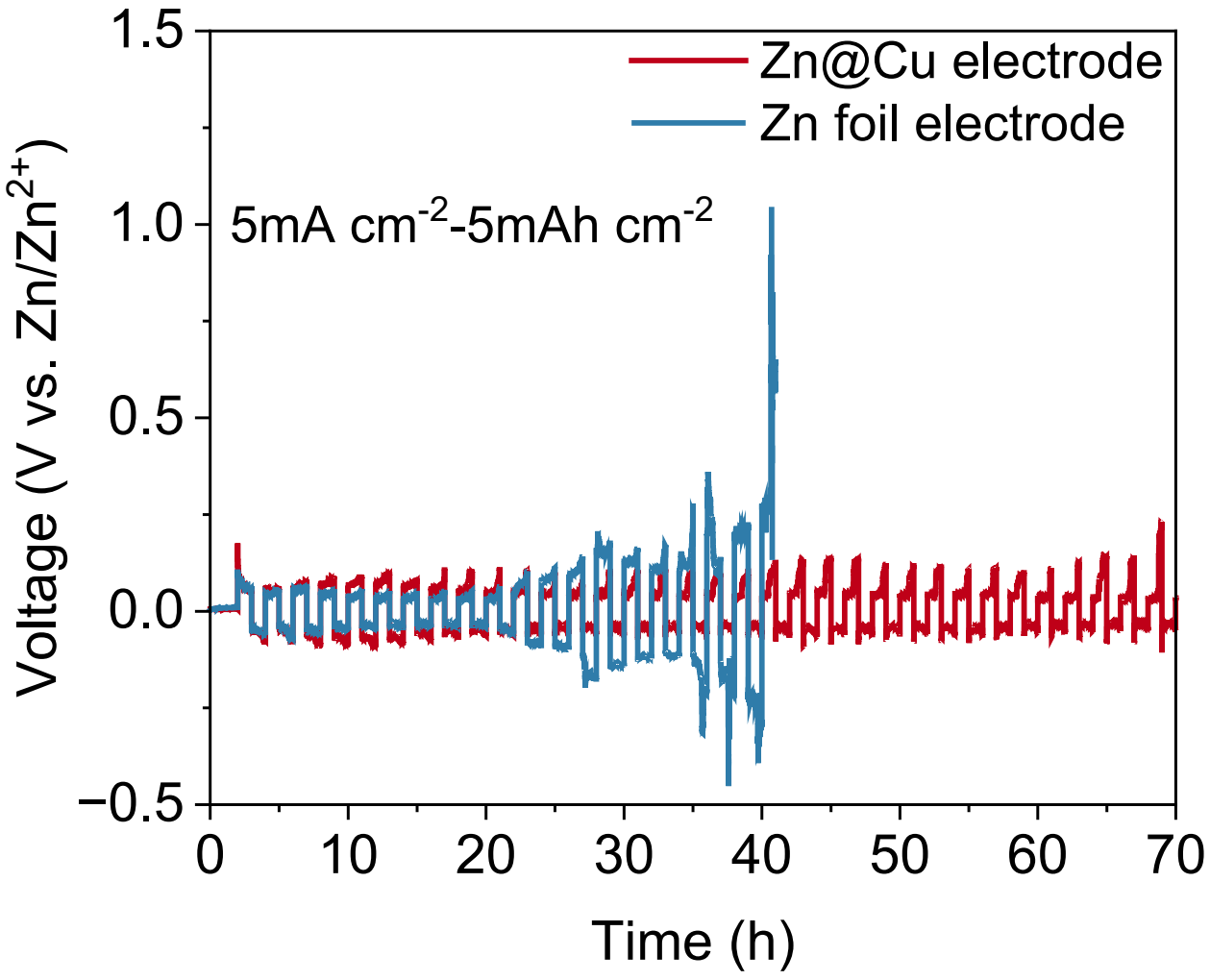

Figure S2d The cycling performance of same mass symmetrical cells based on lean Zn@Cu electrode and Zn foil electrode

a

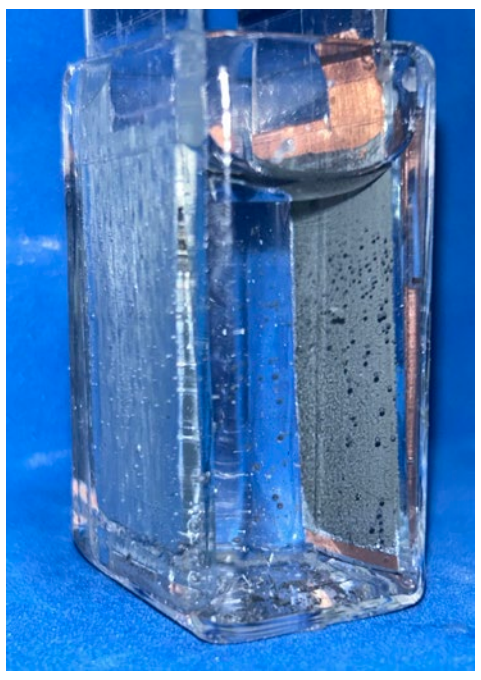

b

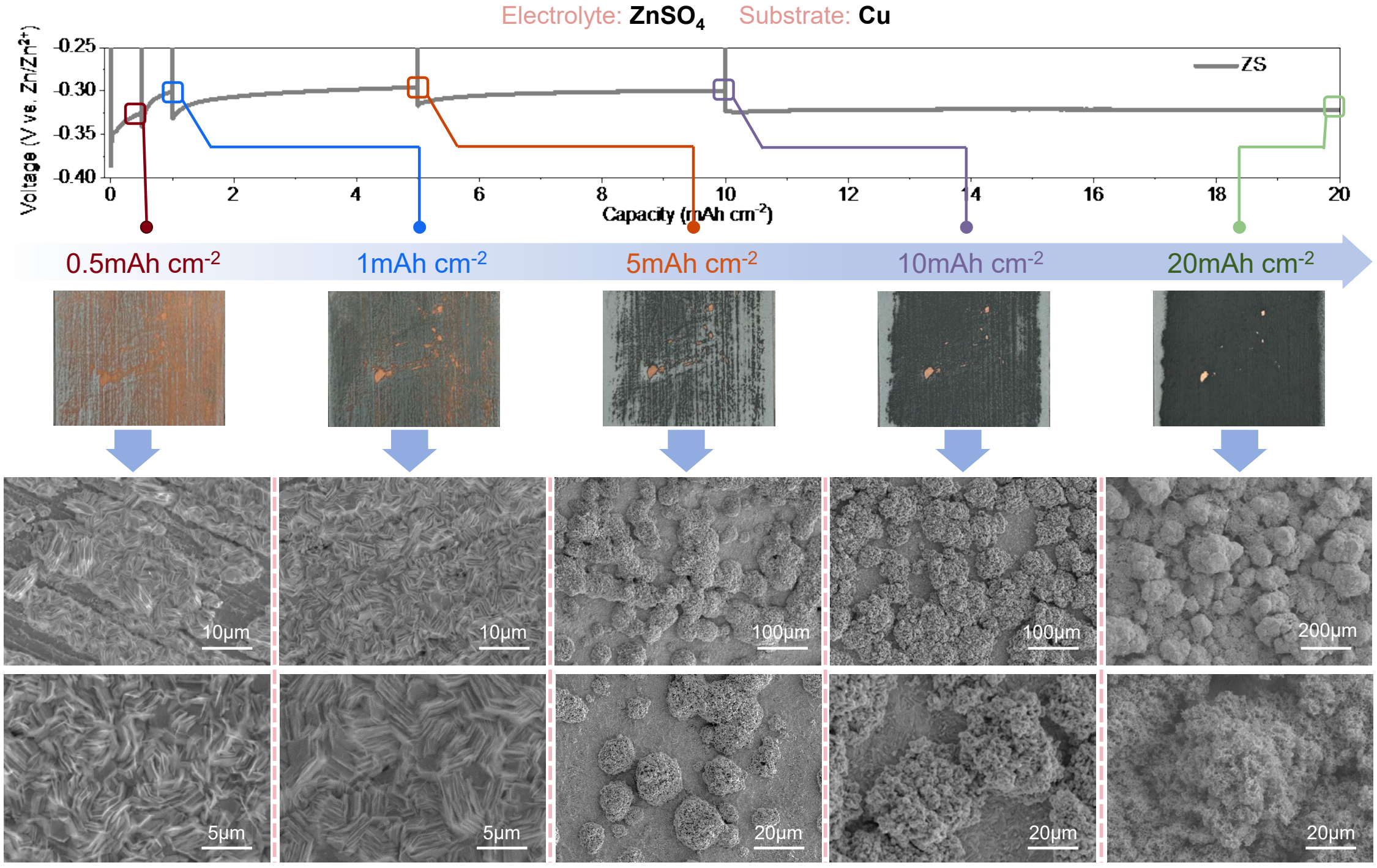

Figure S3 (a) Photo of the quartz cell. (b) Digital photos and SEM images of the gradual formation of pulverized zinc with the increasing plating capacity on the Cu substrate in 2 M $ZnSO_4$ electrolyte.

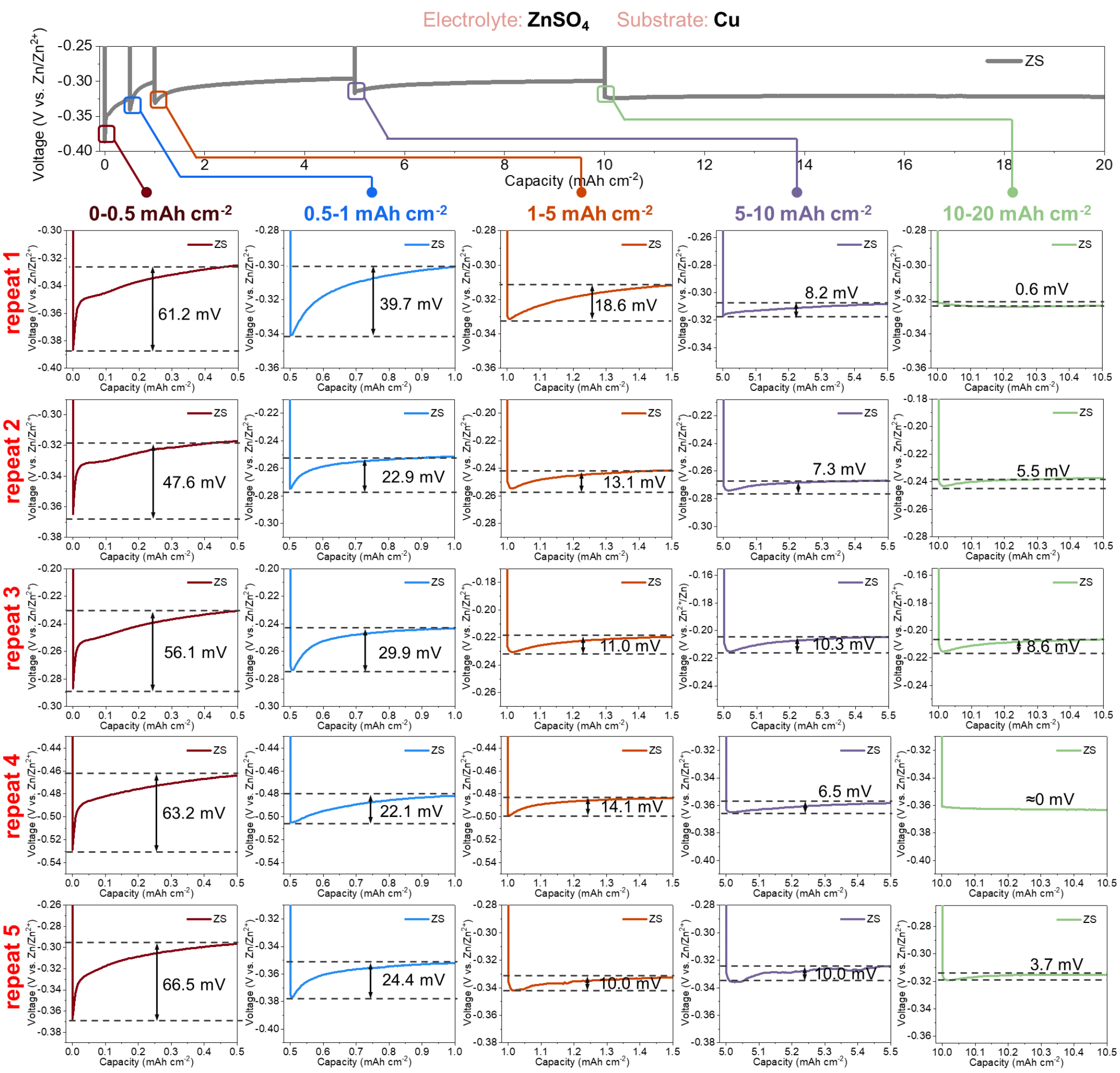

Figure S4 Five sets of repeated experimental data for the nucleation overpotential during the Zn plating capacity increasing from 0 mAh cm^{-2} to 20 mAh cm^{-2} on the Cu substrate in 2 M $ZnSO_4$.

Note S2: 0-0.5 mAh cm^{-2} means that there is a nucleation overpotential deposited on the bare Cu foil. 0.5-1 mAh cm^{-2} means the nucleation overpotential during plating on the basis of 0.5 mAh cm^{-2} zinc. After each plating stage is completed, the electrode sheet was took out from the quartz cell, rinse it thoroughly with alcohol and deionized water, then take photos and SEM image of it. After that, put the electrode sheet back into the original quartz cell to proceed with the following plating process.

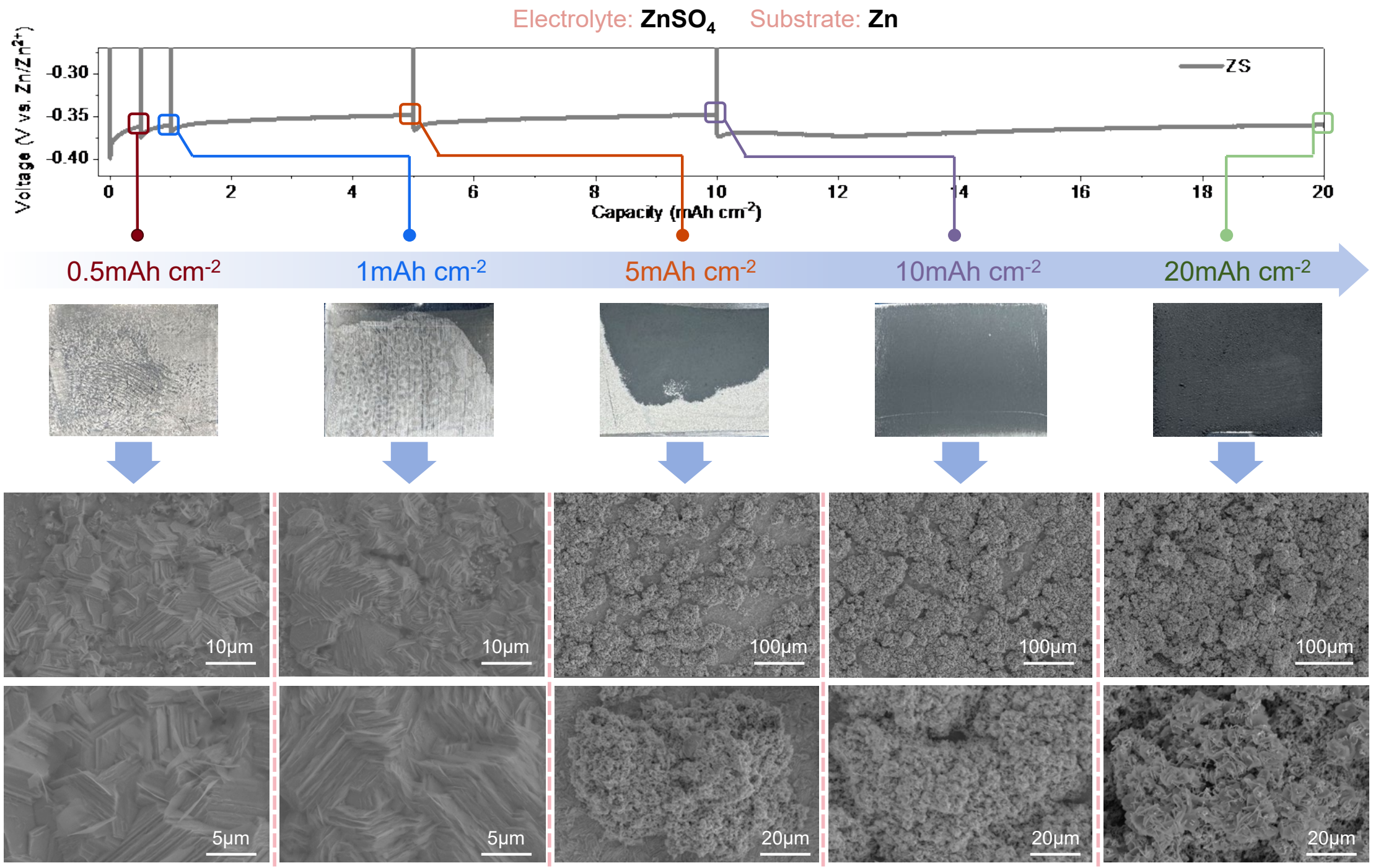

Figure S5 Digital photos and SEM images of the gradual formation of pulverized zinc with the increasing plating capacity on the Zn substrate in 2 M $ZnSO_4$.

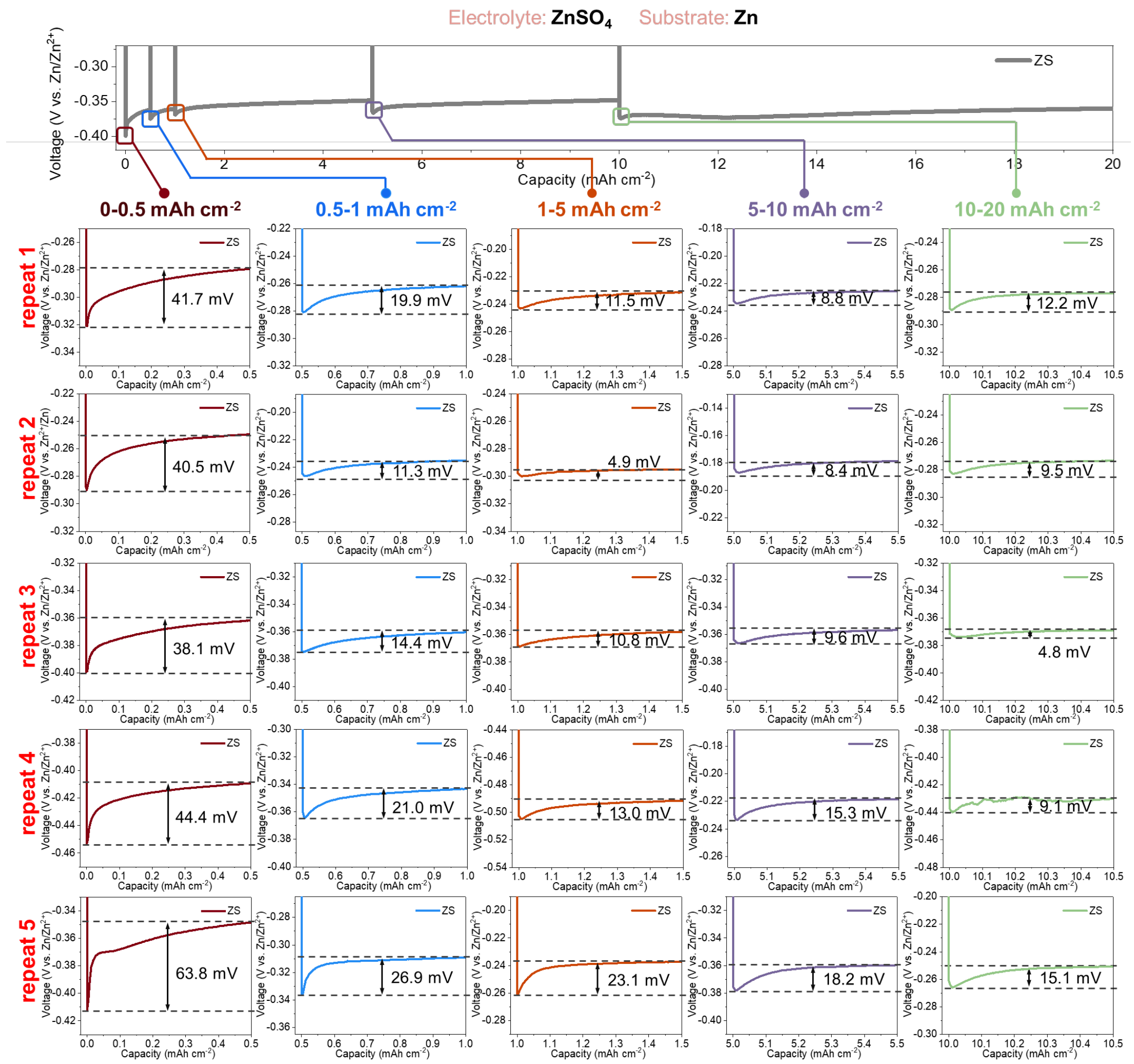

Figure S6 Five sets of repeated experimental data for the nucleation overpotential during the Zn plating capacity increasing from 0 mAh cm^{-2} to 20 mAh cm^{-2} on the Zn substrate in 2 M $ZnSO_4$.

Note 2: 0-0.5 mAh cm^{-2} means that there is a nucleation overpotential deposited on the bare Zn foil. 0.5-1 mAh cm^{-2} means the nucleation overpotential during plating on the basis of 0.5 mAh cm^{-2} zinc. After each plating stage is completed, the electrode sheet was took out from the quartz cell, rinse it thoroughly with alcohol and deionized water, then take photos and SEM image of it. After that, put the electrode sheet back into the original quartz cell to proceed with the following plating process.

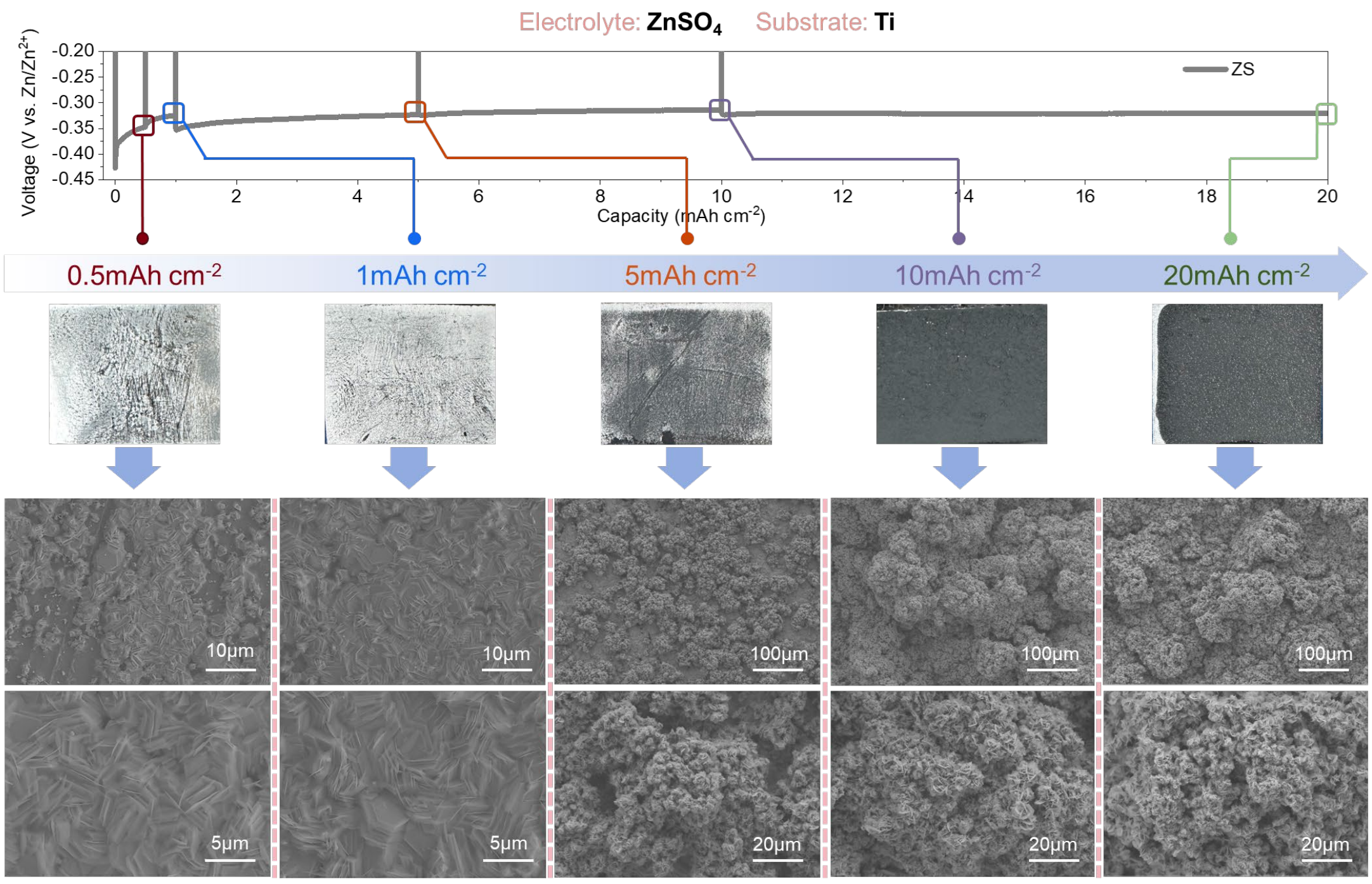

Figure S7 Digital photos and SEM images of the gradual formation of pulverized zinc with the increasing plating capacity on the Ti substrate in 2 M $ZnSO_4$.

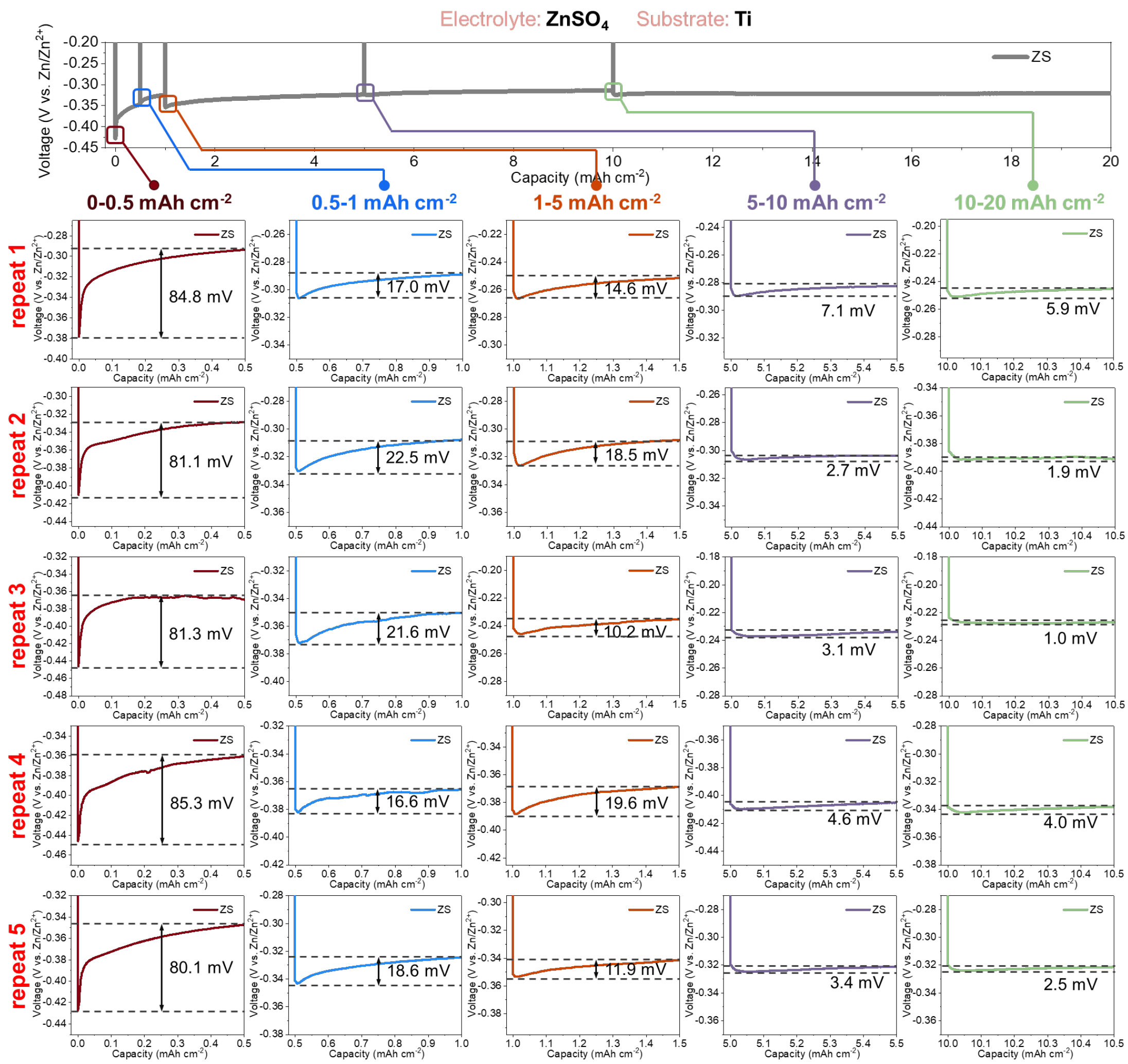

Figure S8 Five sets of repeated experimental data for the nucleation overpotential during the Zn plating capacity increasing from 0 mAh cm^{-2} to 20 mAh cm^{-2} on the Ti substrate in 2 M $ZnSO_4$.

Note 4: 0-0.5 mAh cm^{-2} means that there is a nucleation overpotential deposited on the bare Ti foil. 0.5-1 mAh cm^{-2} means the nucleation overpotential during plating on the basis of 0.5 mAh cm^{-2} zinc. After each plating stage is completed, the electrode sheet was took out from the quartz cell, rinse it thoroughly with alcohol and deionized water, then take photos and SEM image of it. After that, put the electrode sheet back into the original quartz cell to proceed with the following plating process.

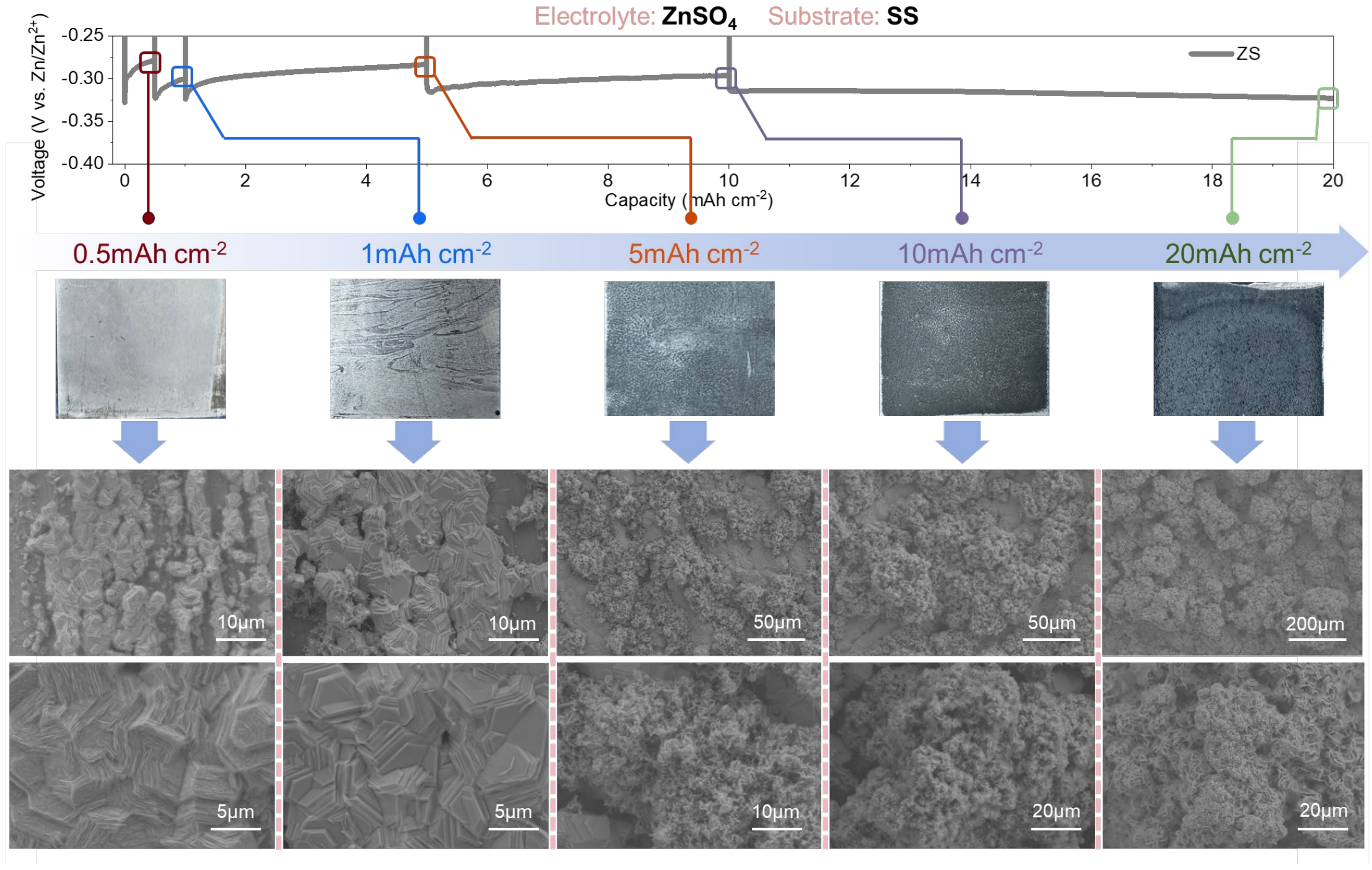

Figure S9 Digital photos and SEM images of the gradual formation of pulverized zinc with the increasing of plating capacity on the SS substrate in 2 M $ZnSO_4$.

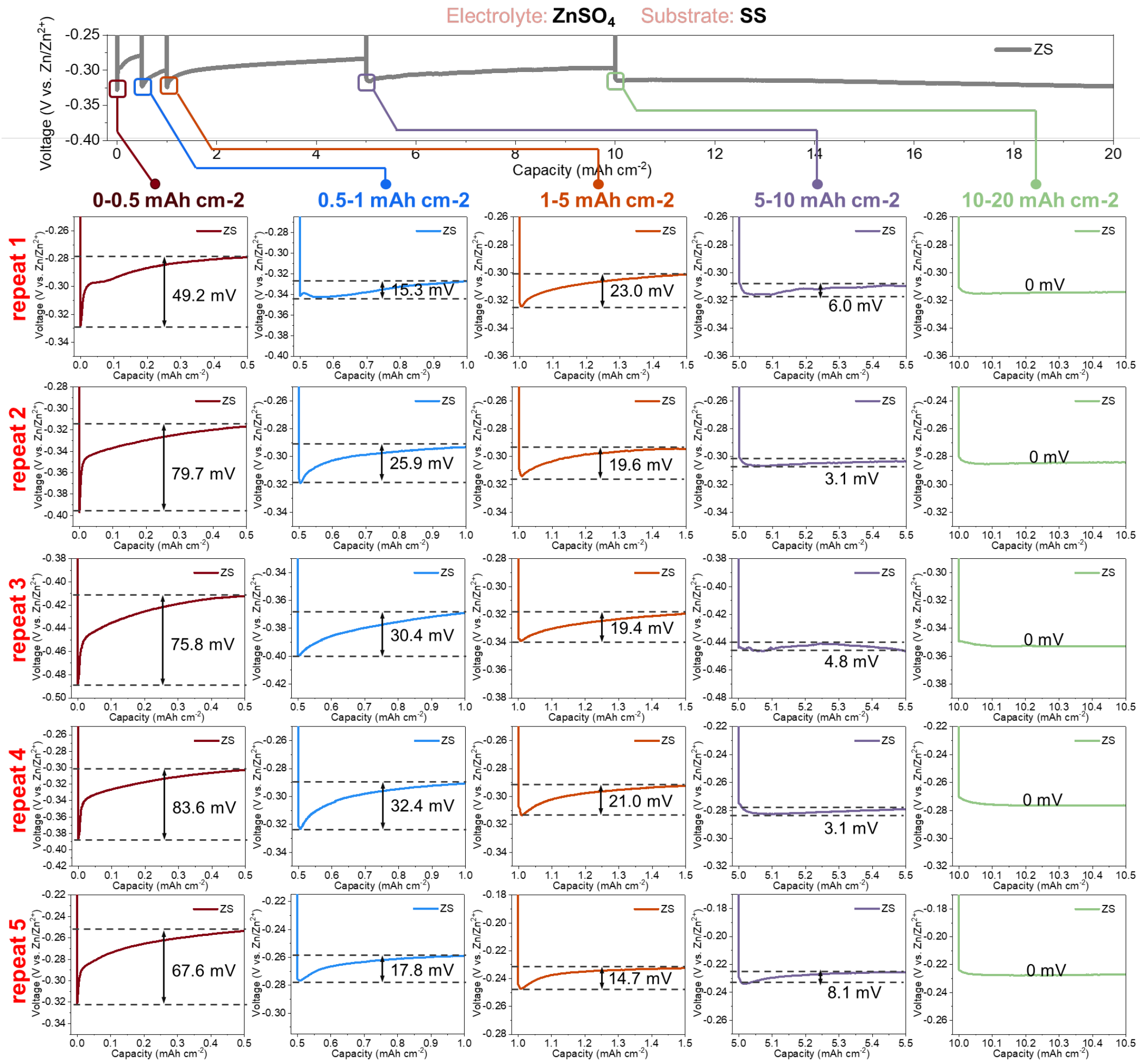

Figure S10 Five sets of repeated experimental data for the nucleation overpotential during the Zn plating capacity increasing from 0 mAh cm^{-2} to 20 mAh cm^{-2} on the SS substrate in 2 M $ZnSO_4$.

Note 5: 0-0.5 mAh cm^{-2} means that there is a nucleation overpotential deposited on the bare SS foil. 0.5-1 mAh cm^{-2} means the nucleation overpotential during plating on the basis of 0.5 mAh cm^{-2} zinc. After each plating stage is completed, the electrode sheet was took out from the quartz cell, rinse it thoroughly with alcohol and deionized water, then take photos and SEM image of it. After that, put the electrode sheet back into the original quartz cell to proceed with the following plating process.

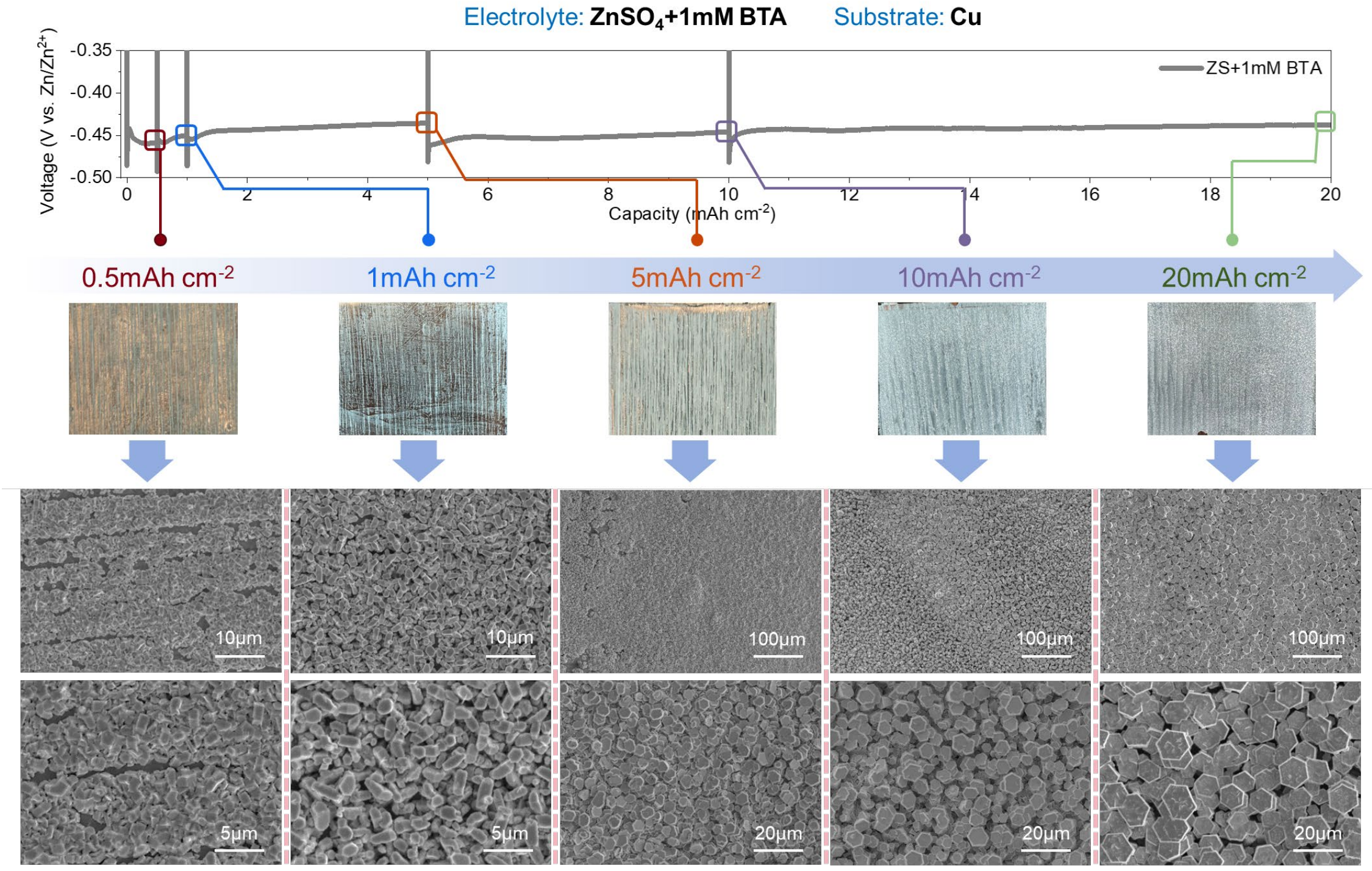

Figure S11 Digital photos and SEM images of the gradual formation of pulverized zinc with the increasing plating capacity on the Cu substrate in 2 M $ZnSO_4$+1mM BTA.

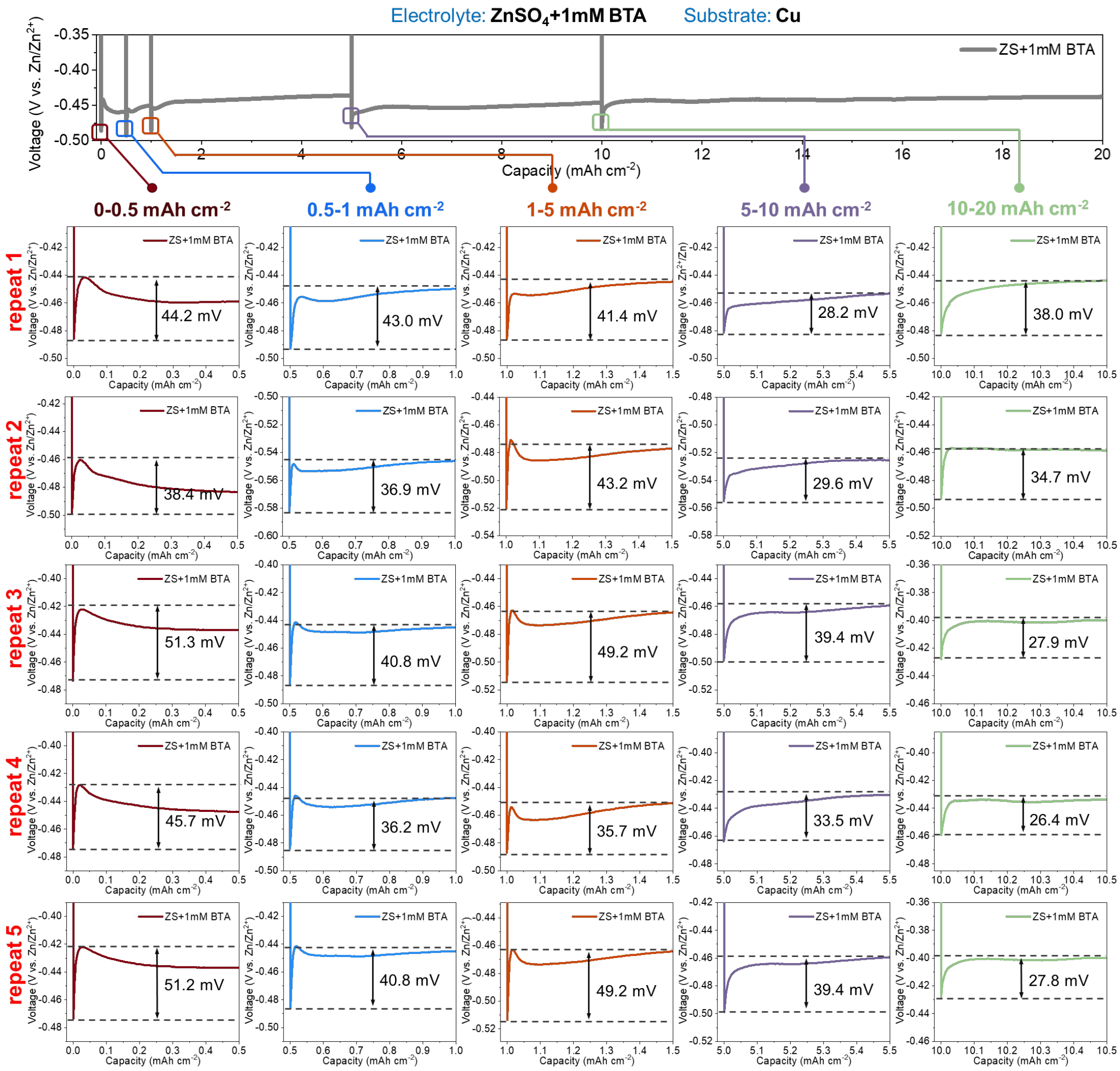

Figure S12 Five sets of repeated experimental data for the nucleation overpotential during the Zn plating capacity increasing from 0 mAh cm^{-2} to 20 mAh cm^{-2} on the Cu substrate in $ZnSO_4$ +1mM BTA.

Note S6: 0-0.5 mAh cm^{-2} means that there is a nucleation overpotential deposited on the bare Cu foil. 0.5-1 mAh cm^{-2} means the nucleation overpotential during plating on the basis of 0.5 mAh cm^{-2} zinc. After each plating stage is completed, the electrode sheet was took out from the quartz cell, rinse it thoroughly with alcohol and deionized water, then take photos and SEM image of it. After that, put the electrode sheet back into the original quartz cell to proceed with the following plating process.

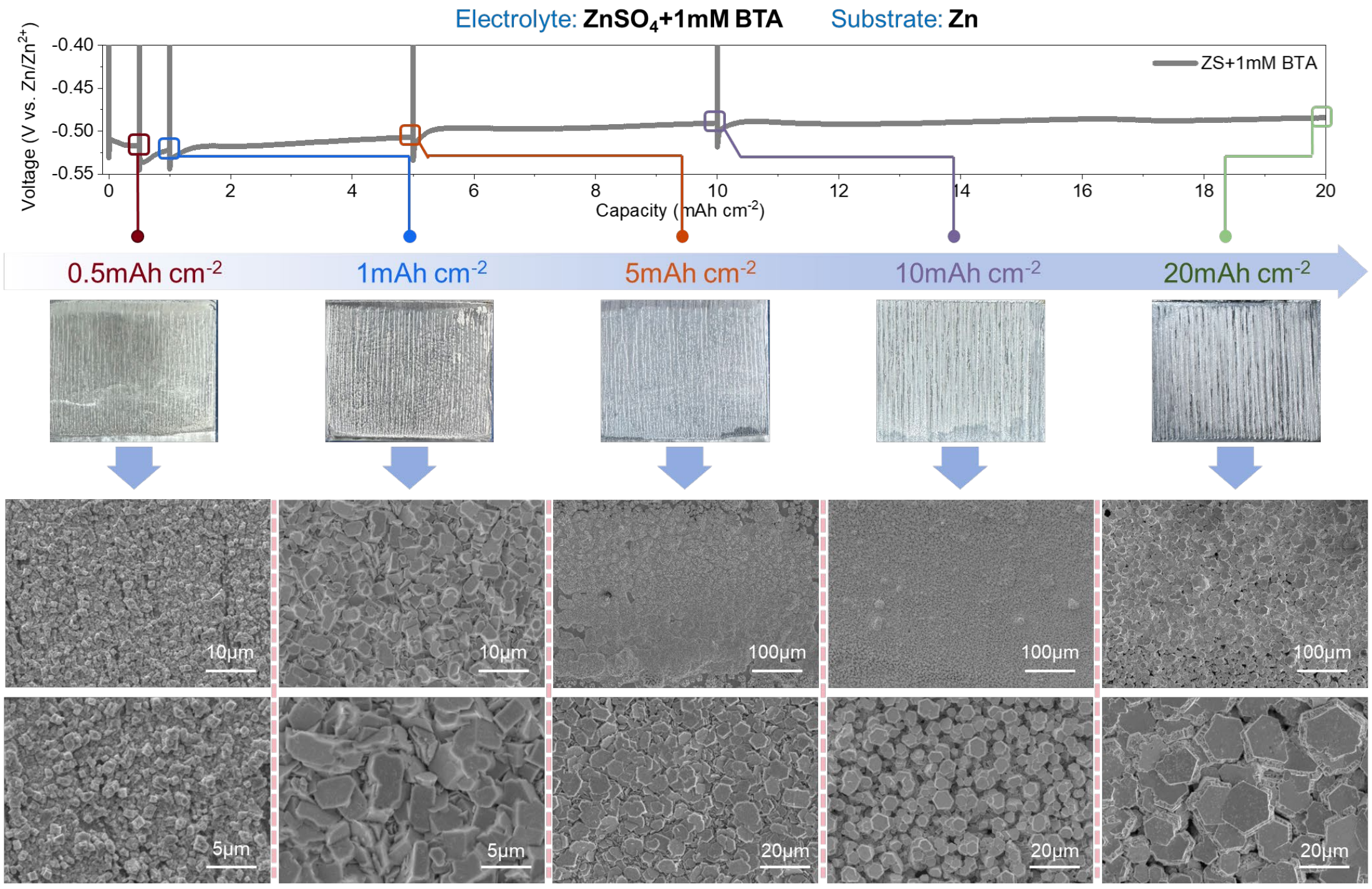

Figure S13 Digital photos and SEM images of the gradual formation of pulverized zinc with the increasing plating capacity on the Zn substrate in 2 M $ZnSO_4$+1mM BTA.

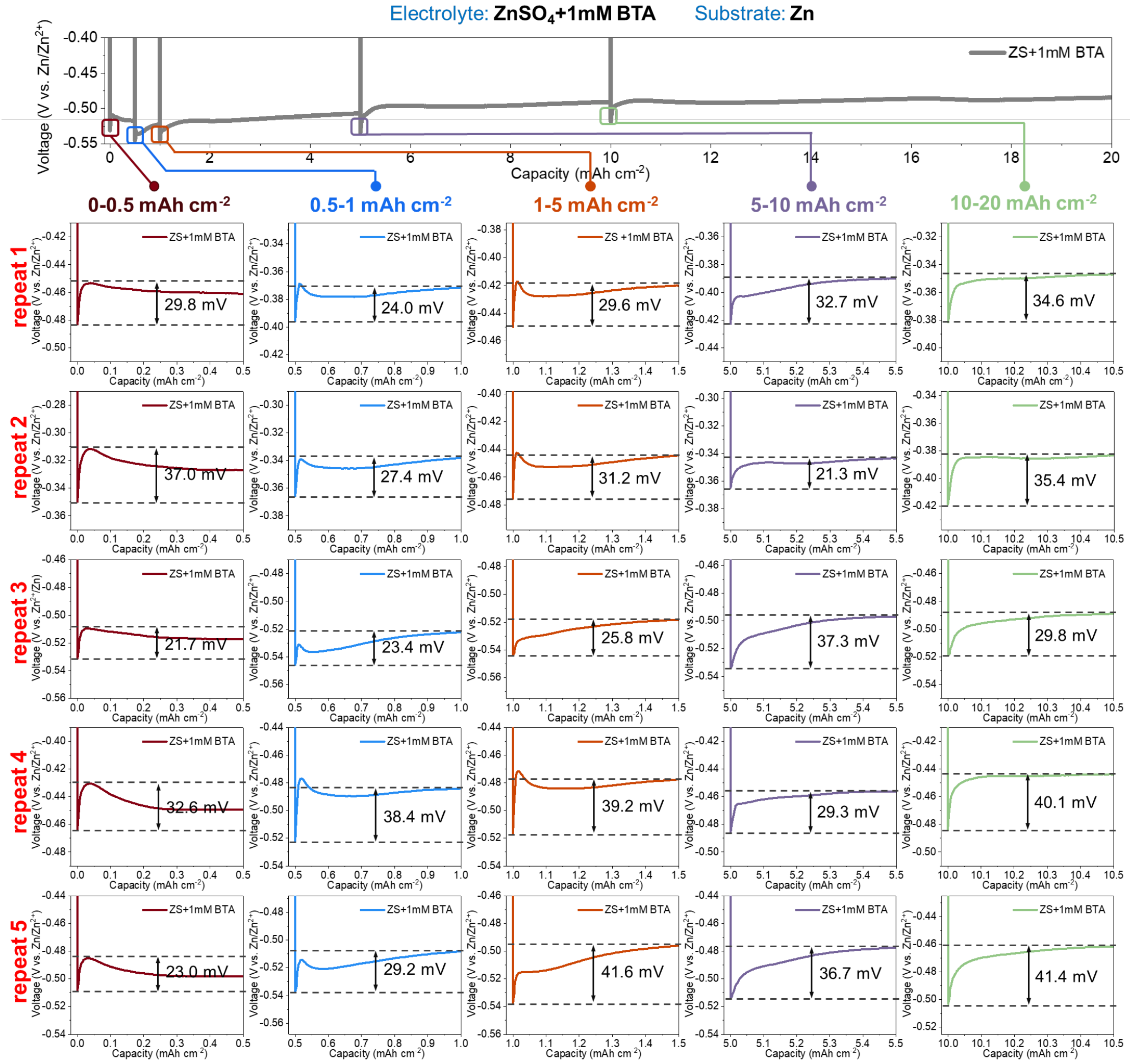

Figure S14 Five sets of repeated experimental data for the nucleation overpotential during the Zn plating capacity increasing from 0 mAh cm^{-2} to 20 mAh cm^{-2} on the Zn substrate in $ZnSO_4$ +1mM BTA.

Note S7:0-0.5 mAh cm^{-2} means that there is a nucleation overpotential deposited on the bare Zn foil. 0.5-1 mAh cm^{-2} means the nucleation overpotential during plating on the basis of 0.5 mAh cm^{-2} zinc. After each plating stage is completed, the electrode sheet was took out from the quartz cell, rinse it thoroughly with alcohol and deionized water, then take photos and SEM image of it. After that, put the electrode sheet back into the original quartz cell to proceed with the following plating process.

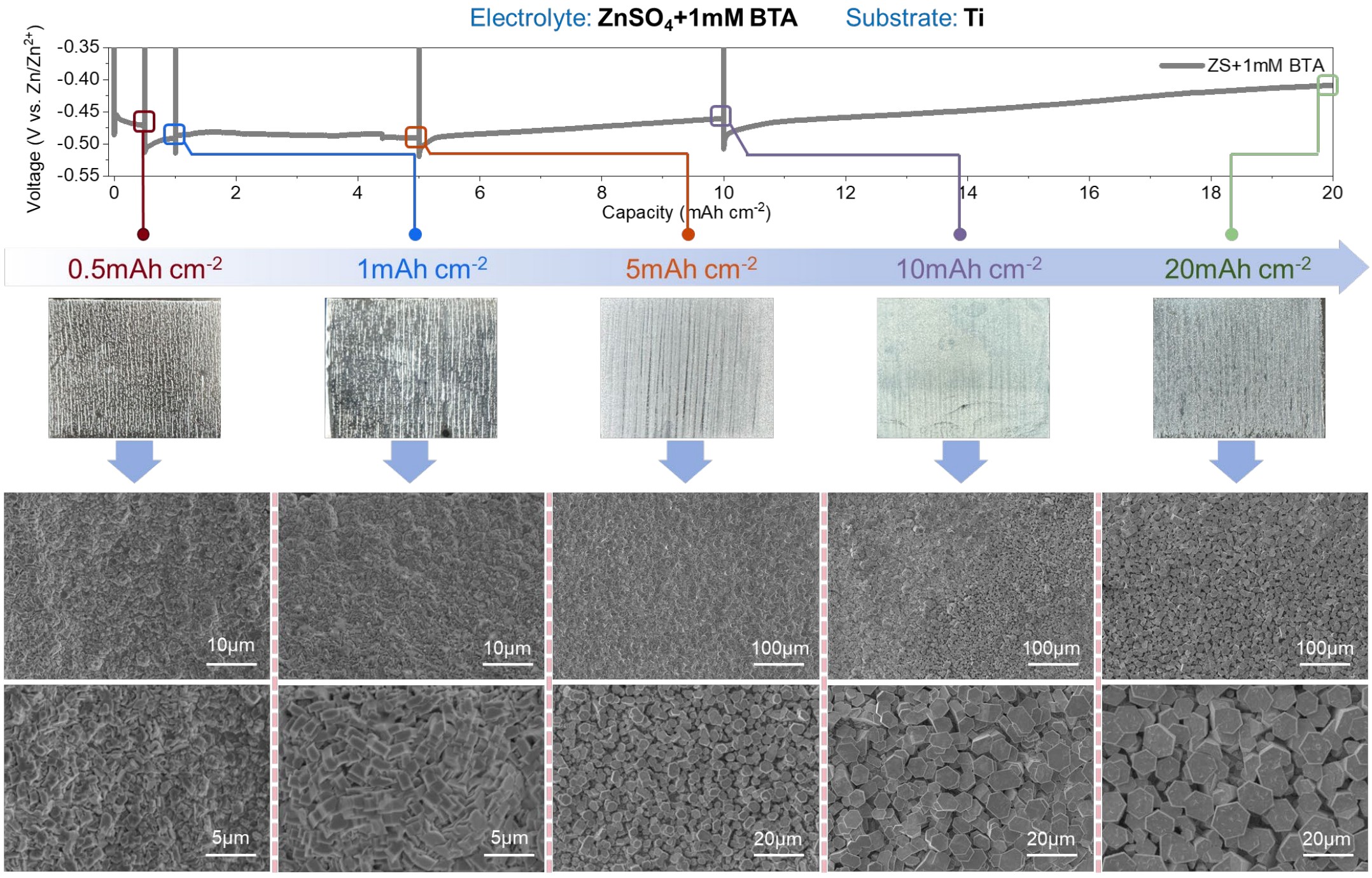

Figure S15 Digital photos and SEM images of the gradual formation of pulverized zinc with the increasing plating capacity on the Ti substrate in 2 M $ZnSO_4$+1mM BTA.

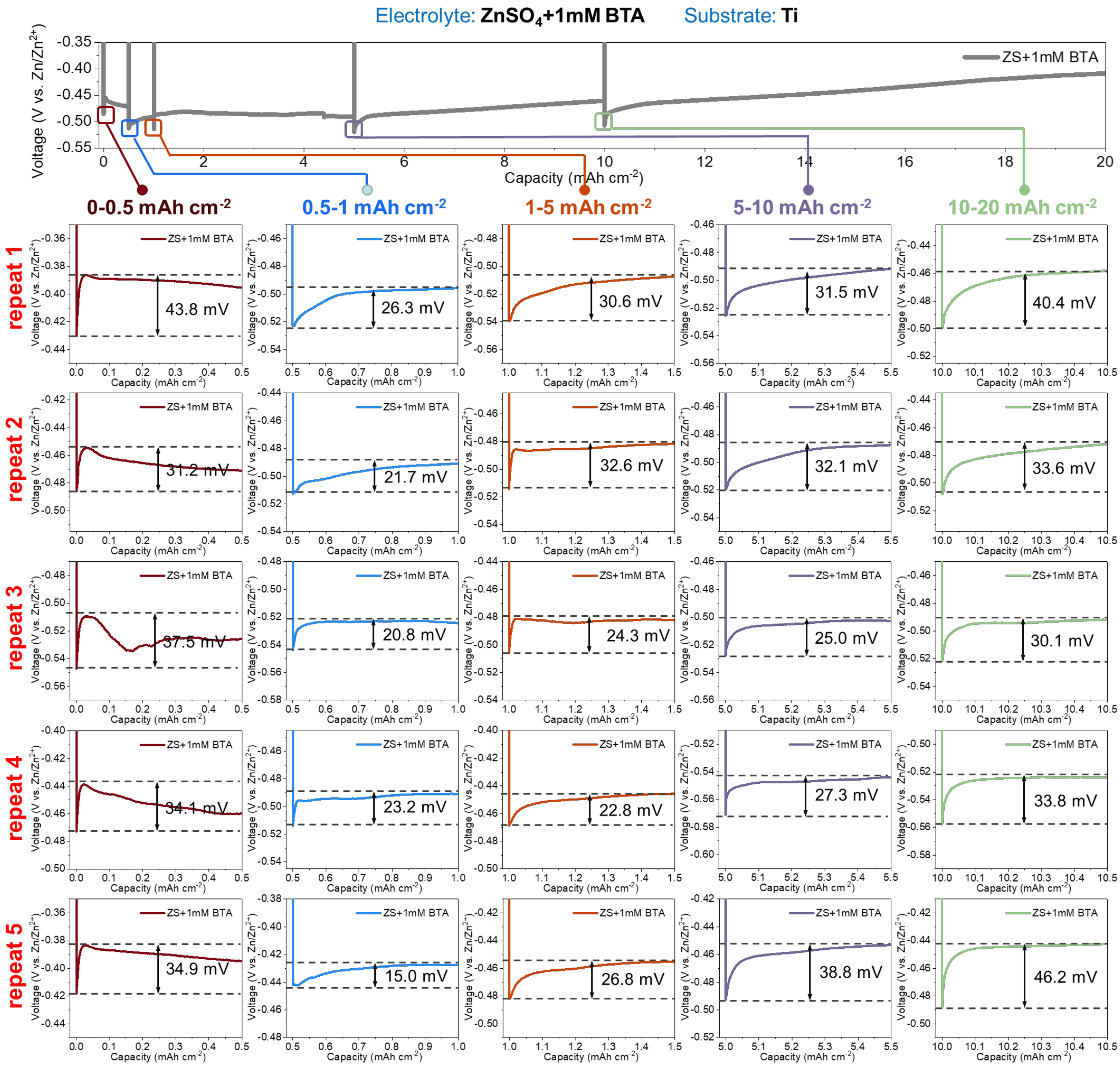

Figure S16 Five sets of repeated experimental data for the nucleation overpotential during the Zn plating capacity increasing from 0 mAh cm^{-2} to 20 mAh cm^{-2} on the Ti substrate in $ZnSO_4$ +1mM BTA.

Note S9: 0-0.5 mAh cm^{-2} means that there is a nucleation overpotential deposited on the bare Ti foil. 0.5-1 mAh cm^{-2} means the nucleation overpotential during plating on the basis of 0.5 mAh cm^{-2} zinc. After each plating stage is completed, the electrode sheet was took out from the quartz cell, rinse it thoroughly with alcohol and deionized water, then take photos and SEM image of it. After that, put the electrode sheet back into the original quartz cell to proceed with the following plating process.

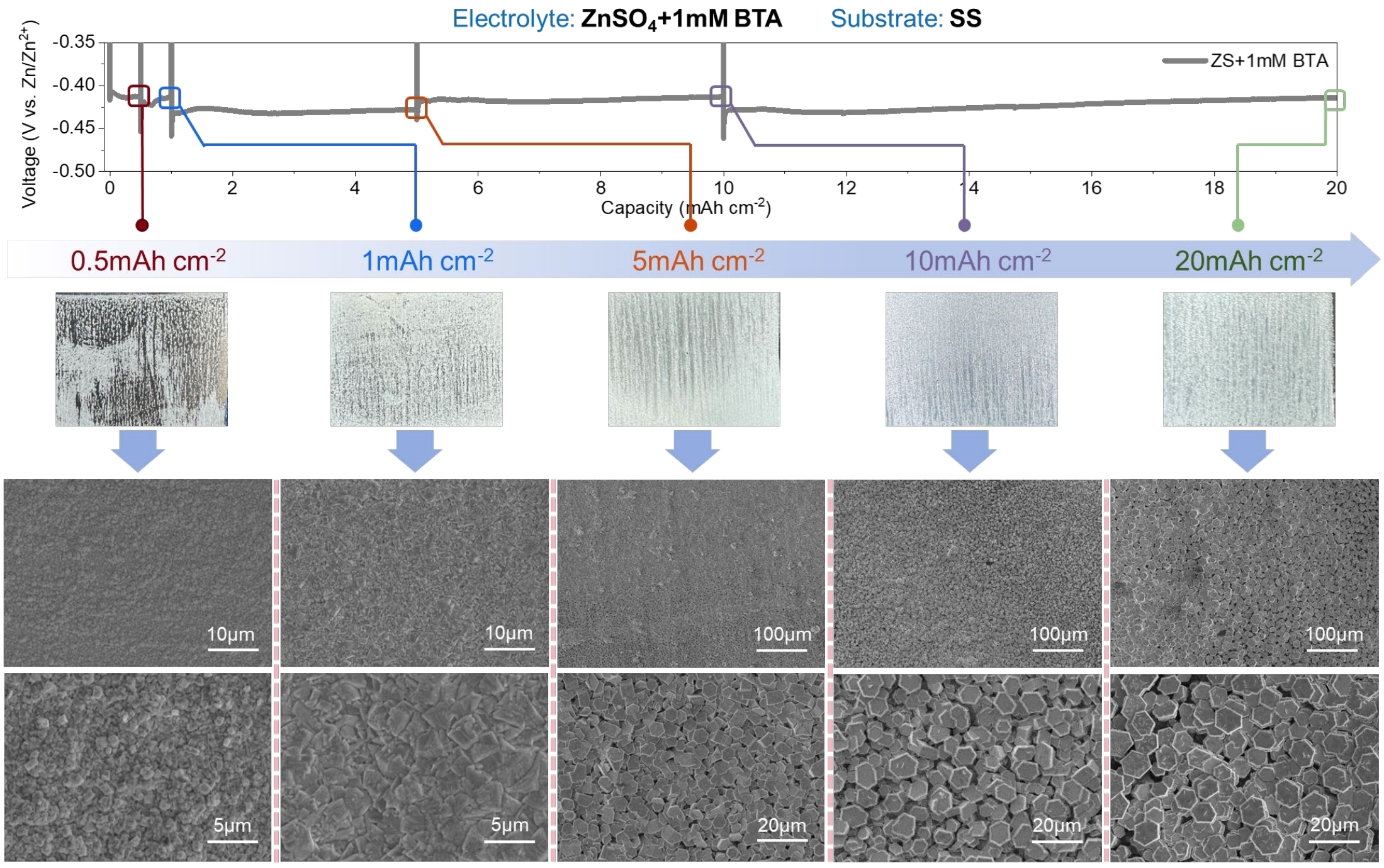

Figure S17 Digital photos and SEM images of the gradual formation of pulverized zinc with the increasing plating capacity on the SS substrate in 2 M $ZnSO_4$+1mM BTA.

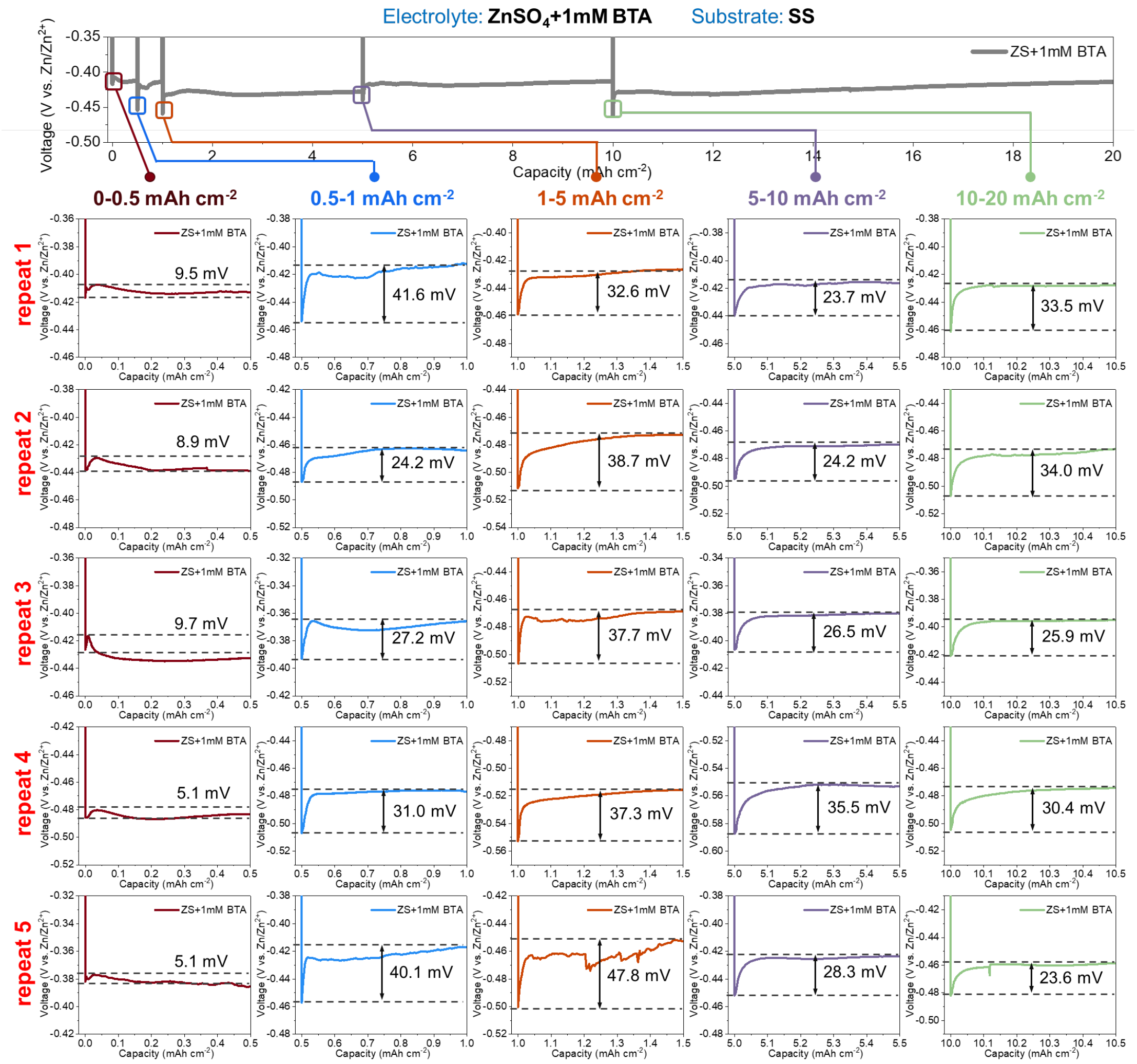

Figure S18 Five sets of repeated experimental data for the nucleation overpotential during the Zn plating capacity increasing from 0 mAh cm^{-2} to 20 mAh cm^{-2} on the SS substrate in $ZnSO_4$ +1mM BTA.

Note S9: 0-0.5 mAh cm^{-2} means that there is a nucleation overpotential deposited on the bare SS foil. 0.5-1 mAh cm^{-2} means the nucleation overpotential during plating on the basis of 0.5 mAh cm^{-2} zinc. After each plating stage is completed, the electrode sheet was took out from the quartz cell, rinse it thoroughly with alcohol and deionized water, then take photos and SEM image of it. After that, put the electrode sheet back into the original quartz cell to proceed with the following plating process.

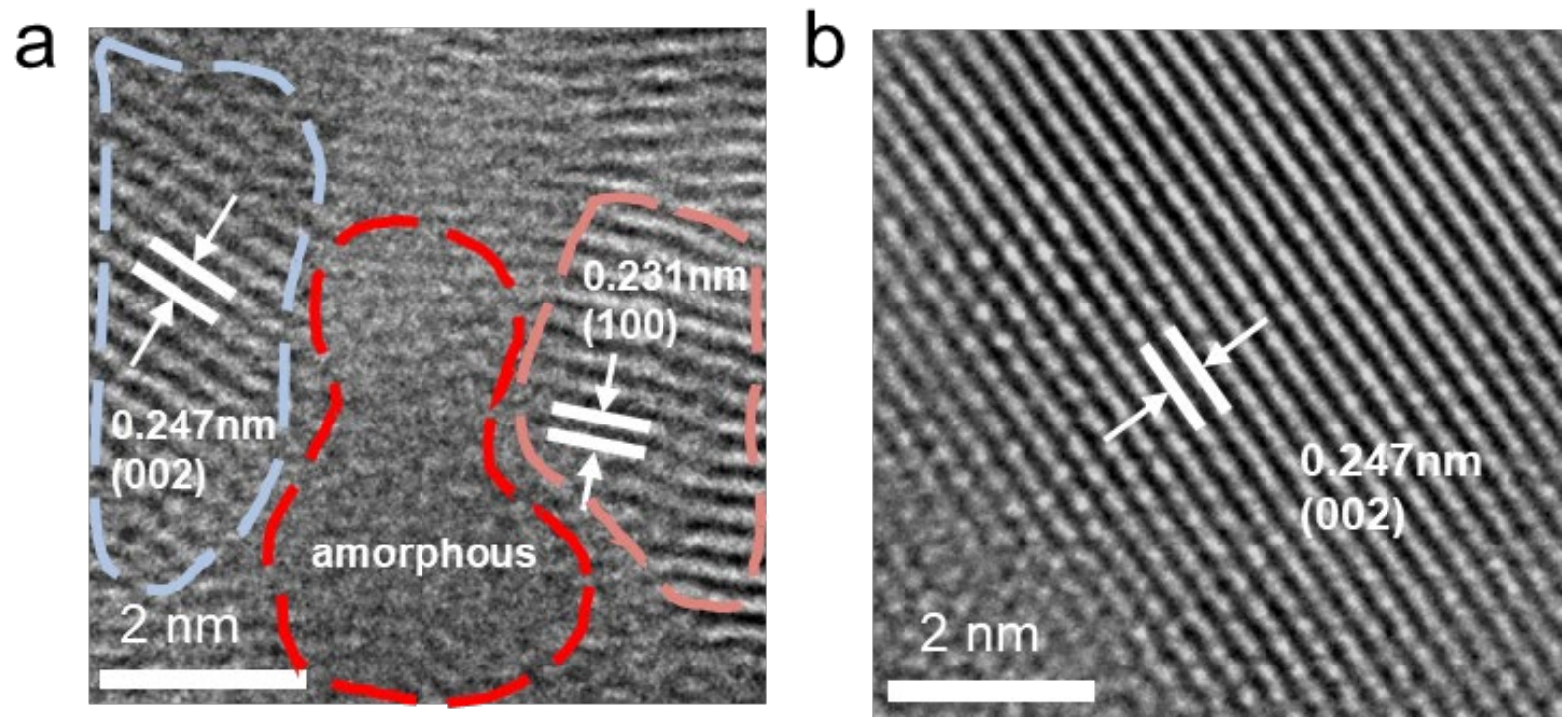

Figure S19 TEM image of Zn electrodeposits on Cu substrate in $ZnSO_4$ (a) and $ZnSO_4$+ 1mM BTA electrolytes(b).

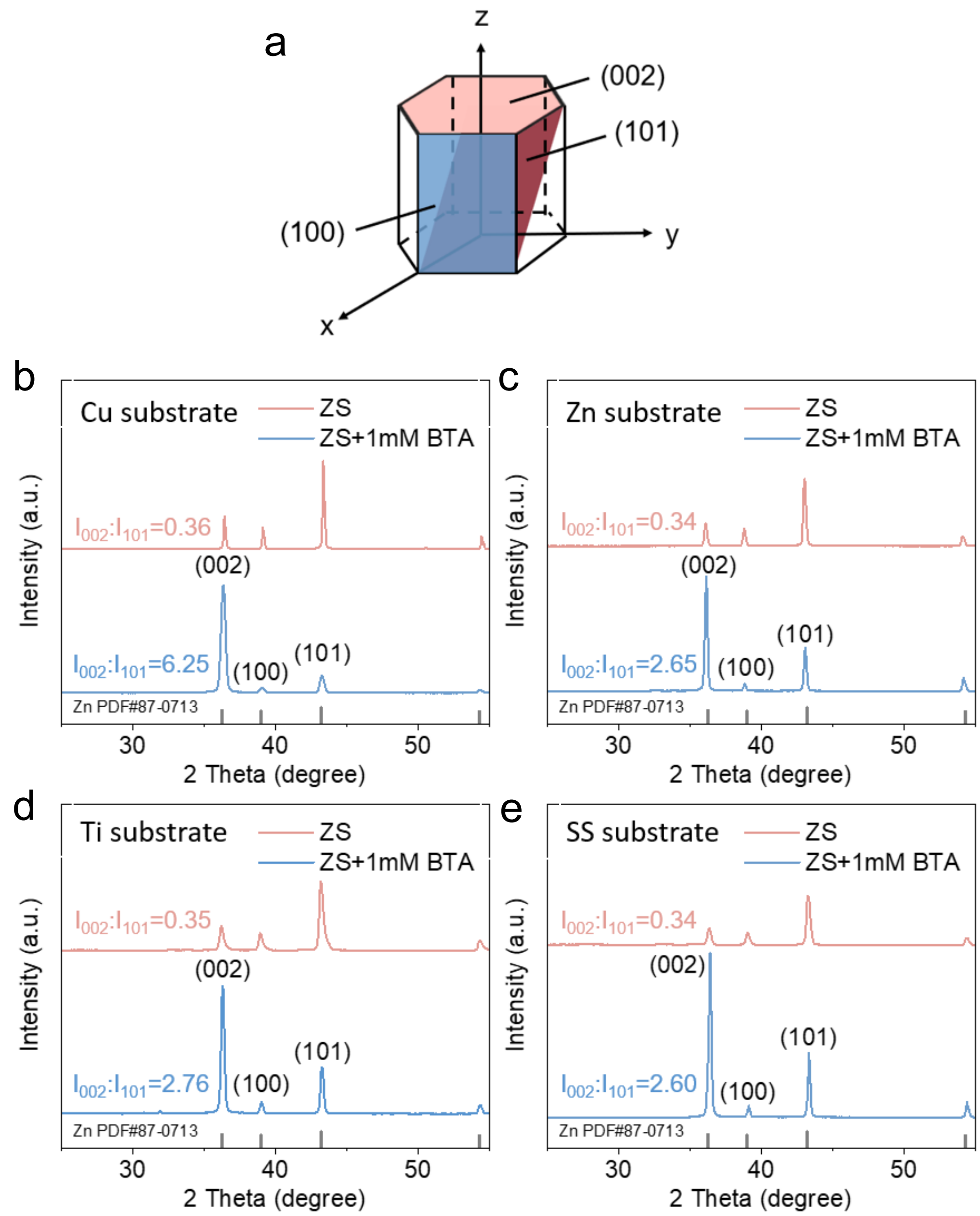

Figure S20 (a) Schematic diagram of growth direction of Zn lattice. XRD patterns of the Zn deposits on Cu (b), Zn (c), Ti (d) and SS (e) substrates with $ZnSO_4$ and $ZnSO_4$+1mM BTA electrolytes.

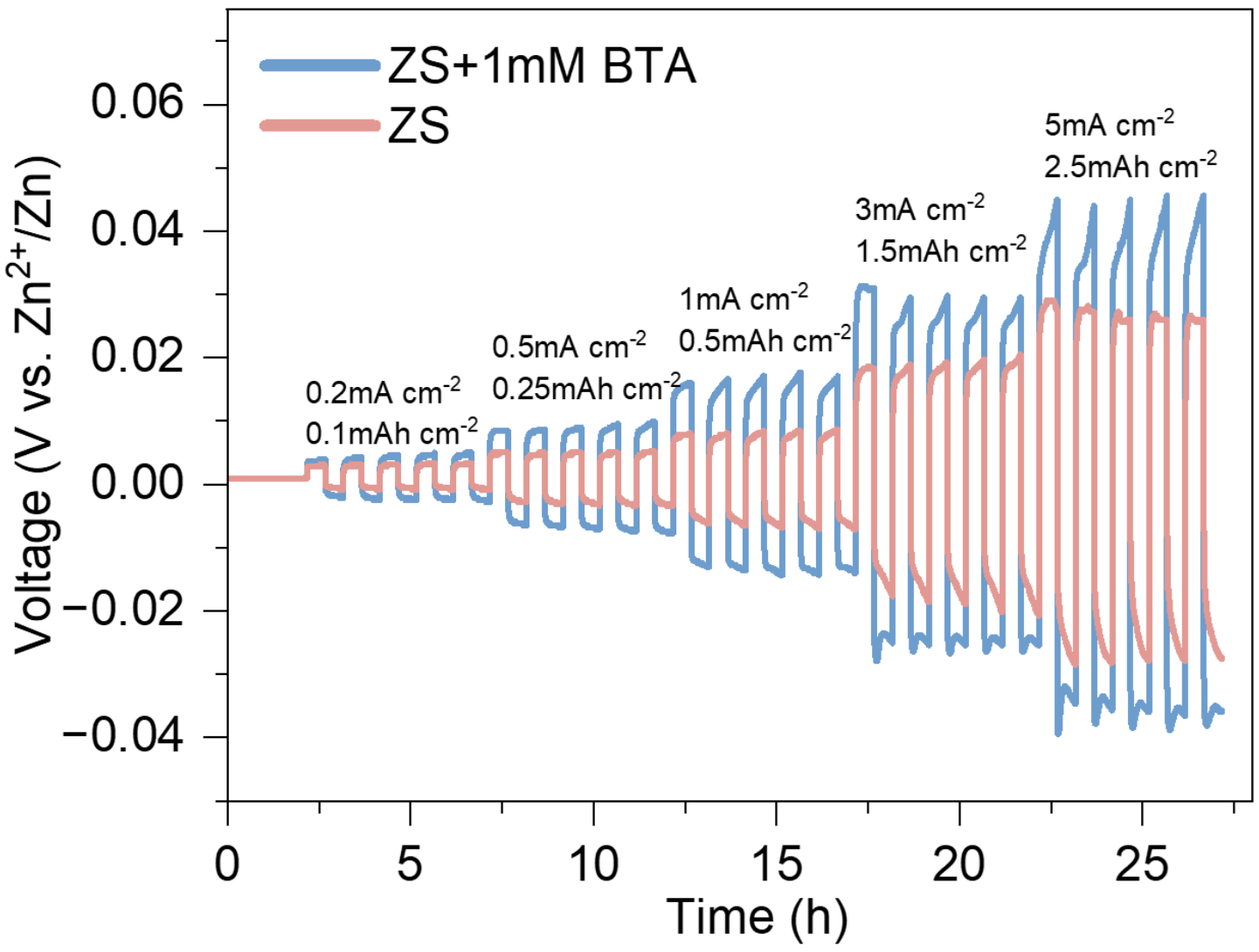

Figure S21 Galvanostatic cycling test of Zn@CuZn@Cu symmetric cells in different electrolytes are conducted at various current density.

Note S10: Apply currents of 0.2 mA cm^{-2}, 0.5 mA cm^{-2}, 1 mA cm^{-2}, 3 mA cm^{-2}, and 5 mA cm^{-2} in sequence, and cycle each current 5 times. Finally, plot a graph of the relationship between overpotential and current. The exchange current densities associated with Zn deposition were calculated *via* Eq.(1)

$$i = i_0 \frac{F}{RT} \frac{\eta}{2} \quad (1)$$

where i is the current density, i_0 is the exchange current density, η is the total overpotential, F is the Faraday constant, R is the gas constant, and T is the absolute temperature

Note S11: **Fig.5b** and **Fig.5c** shows the Tafel polarization plots and the corresponding fitted linear portions according to the Tafel Eq.(2):

$$\eta = b \log(j) + a \qquad (2)$$

where j is the current density, η is the overpotential, and b is the Tafel slope

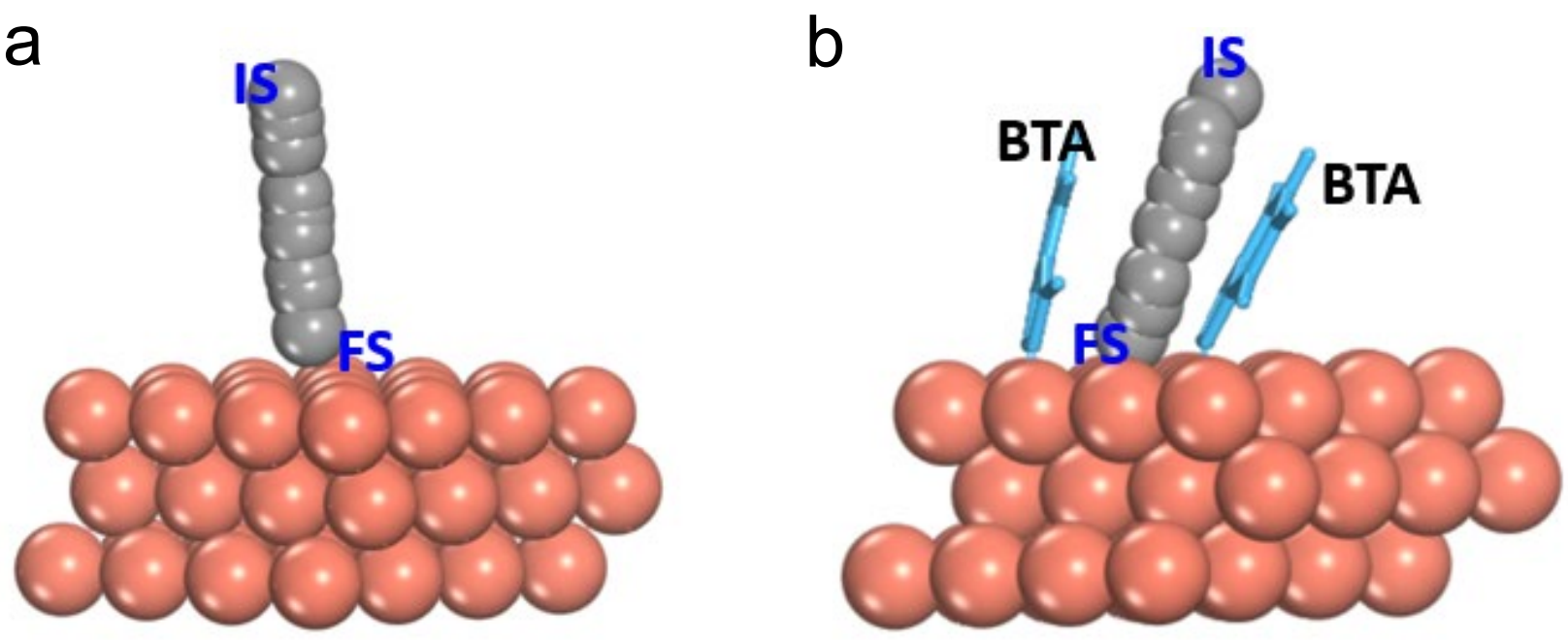

Figure S22 Computational investigation of Zn^{2+} adsorption on Cu (111) without considering H_2O molecules when two BTA molecules adsorbed. a bare Cu (111) substrate. **b** Cu (111) substrate with two BTA molecules absorbed.

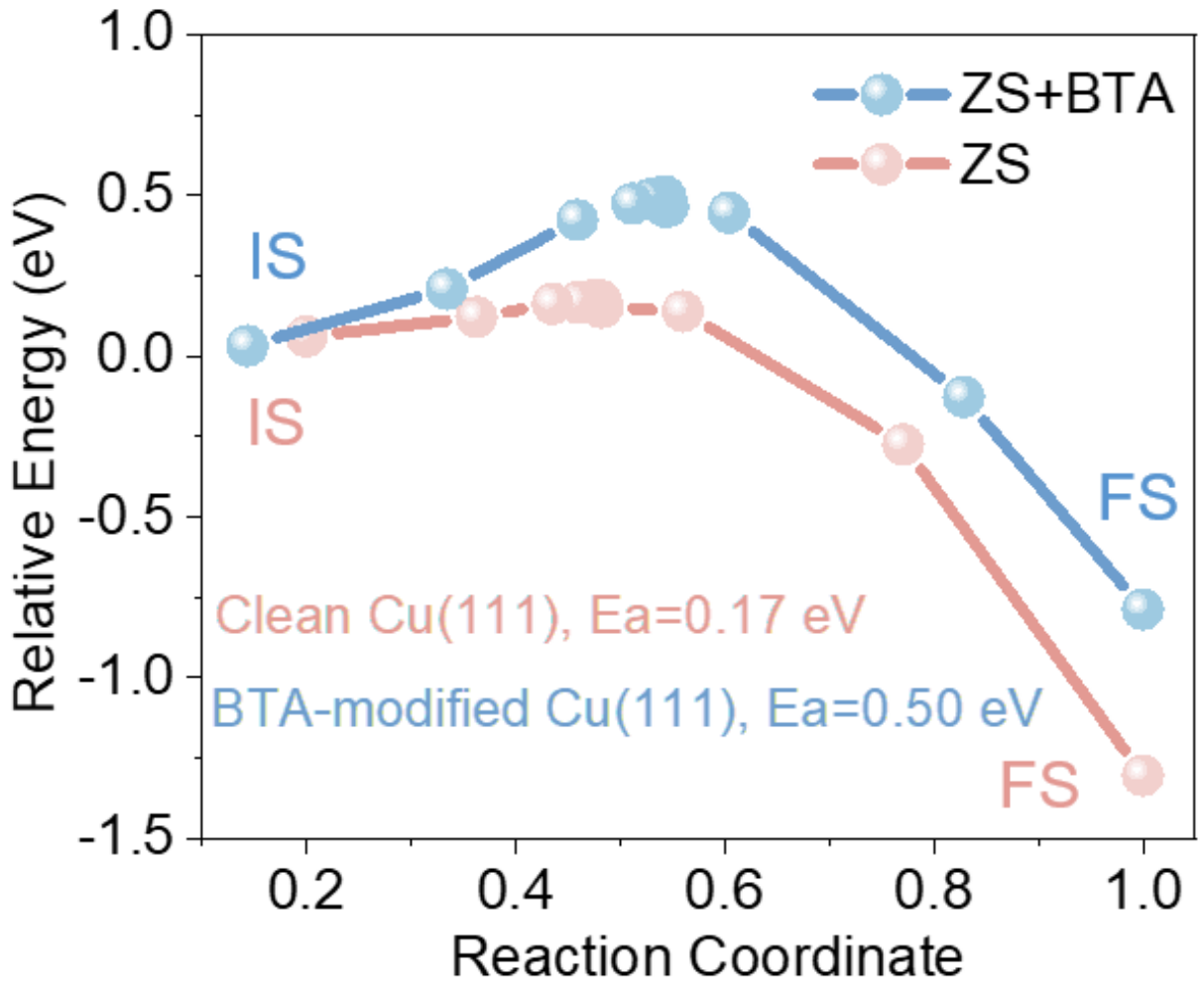

Figure S23 Energy profile for the transition state searching according to the model in Figure S20. Diffusion path has been illustrated with a series of intermediate images generated during transition state searching. During the searching, water environment has been modeled with implicit solution model.

Note S12: In this model, the interference of H_2O molecules is excluded, and the number of BTA molecules is changed from 1 to 2 (**Figure S22)**, in order to more clearly illustrate the inhibitory effect of BTA on Zn^{2+} ions. Using IS energy as a reference, a potential barrier of Ea=0.17eV was experienced in the bare $ZnSO_4$ electrolyte. In the $ZnSO_4$+BTA electrolyte, due to the absorption of BTA additive on the electrode, the potential barrier Ea increases to 0.50 eV (**Figure S23**).

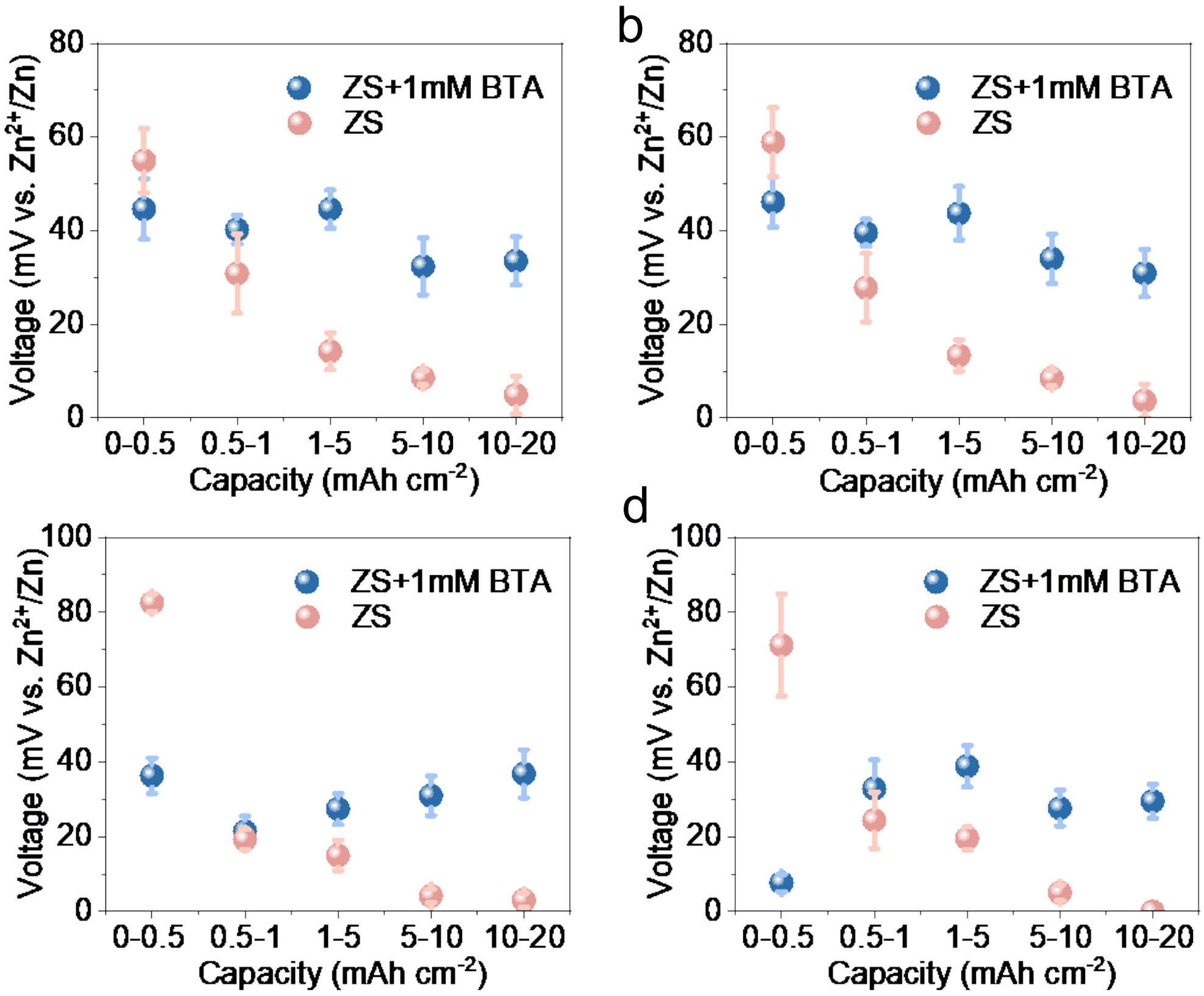

Figure S24 Nucleation overpotential trends at different Zn plating capacities on Cu (a), Zn (b), Ti (c), and SS (d) substrates. Each bar represents five data points. 0-0.5 mAh cm^{-2} means that there is a nucleation overpotential deposited on the bare Cu foil. 0.5-1 mAh cm^{-2} means the nucleation overpotential during plating on the basis of 0.5 mAh cm^{-2} zinc.1-5 mAh cm^{-2} means the nucleation overpotential during plating on the basis of 1 mAh cm^{-2} zinc. 5-10 mAh cm^{-2} means the nucleation overpotential during plating on the basis of 5 mAh cm^{-2} zinc. 10-20 mAh cm^{-2} means the nucleation overpotential during plating on the basis of 10 mAh cm^{-2} zinc

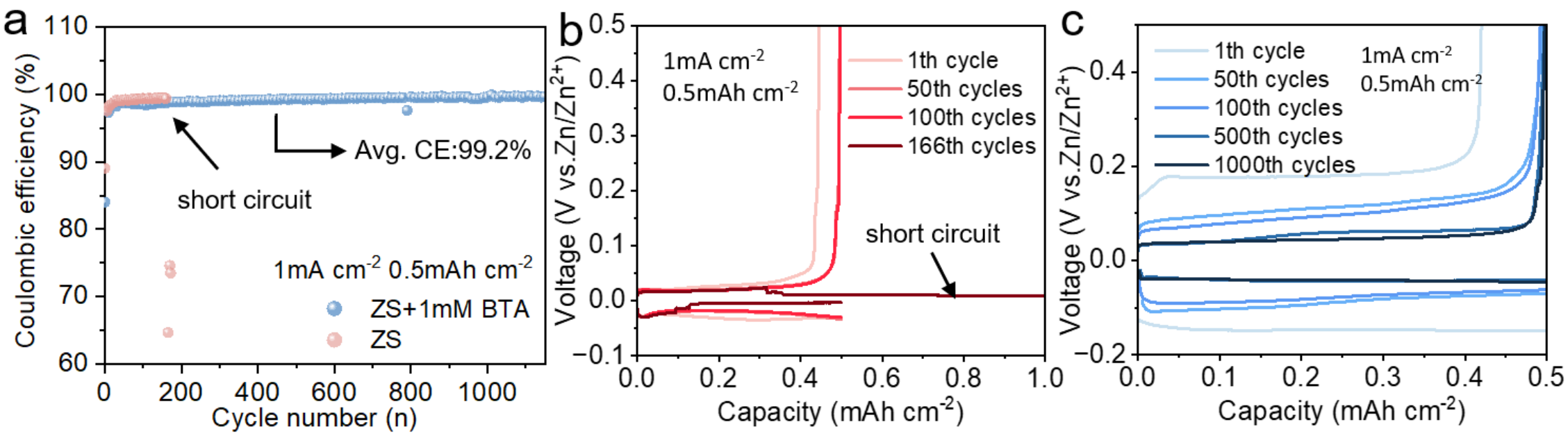

Figure S25 (a) Coulombic efficiency (CE) of ZnCu cells in $ZnSO_4$ and $ZnSO_4$+1mM BTA electrolytes and corresponding voltage profiles (b, c) at 1 mA cm^{-2}- 0.5 mAh cm^{-2}.

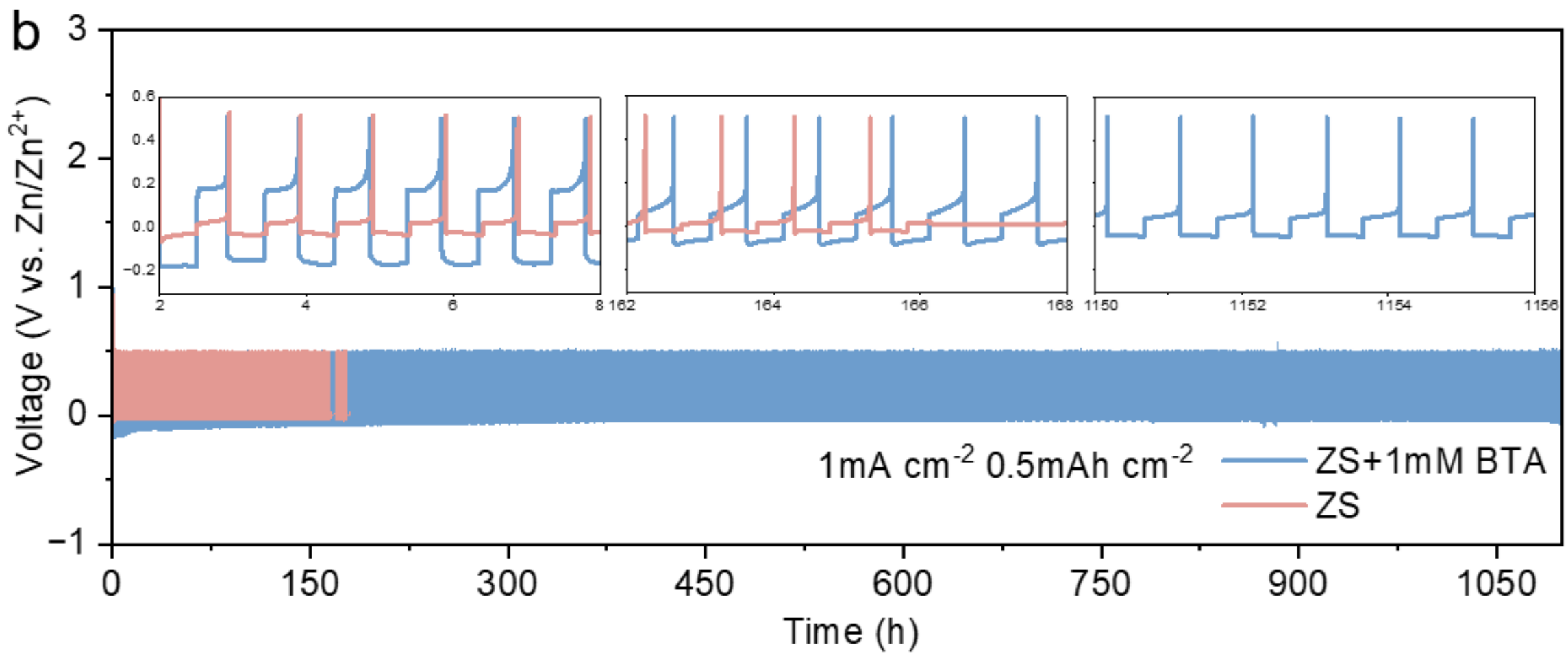

Figure S26 Voltage distribution of ZnCu cell in $ZnSO_4$+1mM BTA electrolyte at 1 mA cm^{-2}-0.5 mAh cm^{-2}.

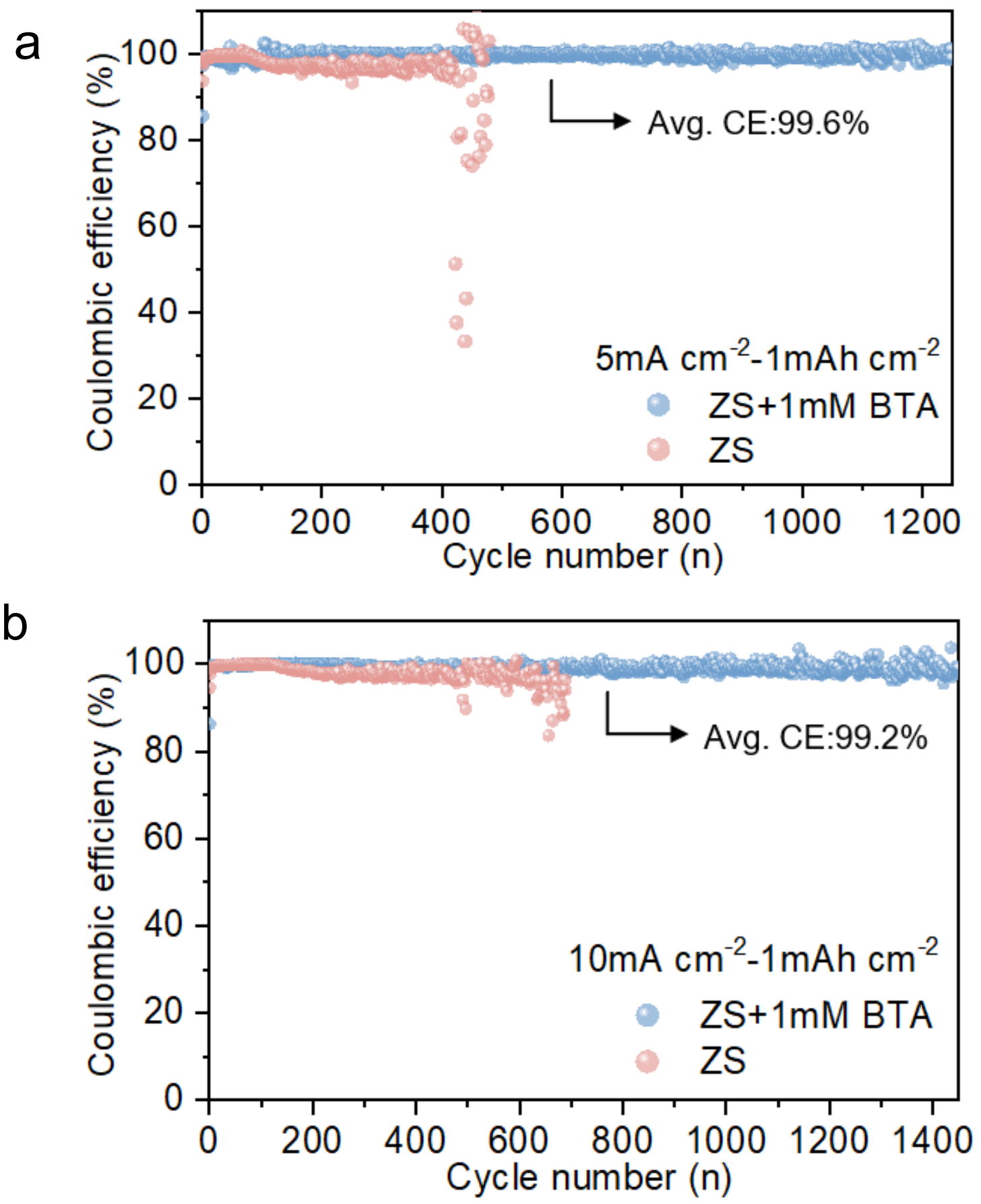

Figure S27 (a) CE of ZnCu cells in $ZnSO_4$ electrolytes with/without the BTA at 5mA cm^{-2} and 1mAh cm^{-2}. (b) CE of ZnCu cells in $ZnSO_4$ electrolytes with/without the BTA at 10mA cm^{-2} and 1mAh cm^{-2}.

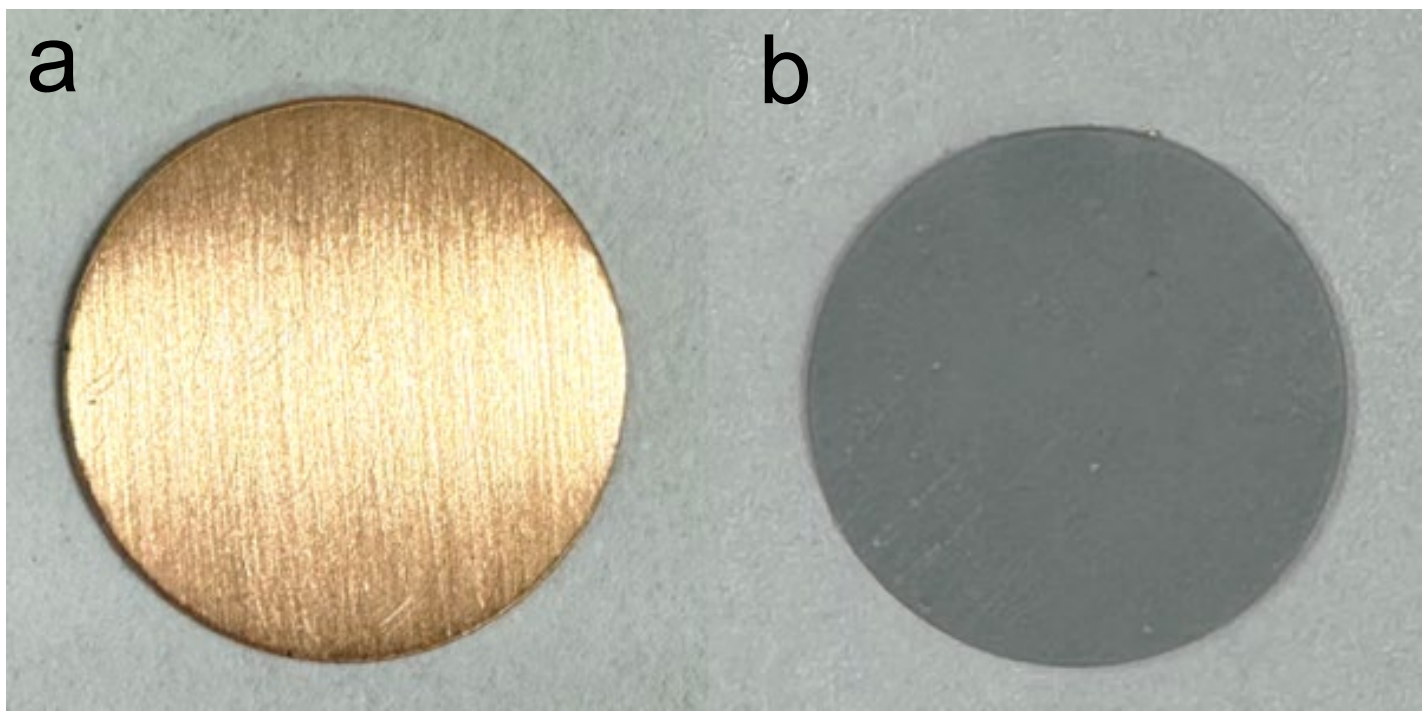

Figure S28 Cu substrate (a) and lean-zinc anode with pre-deposited 2mAh cm^{-2} zinc

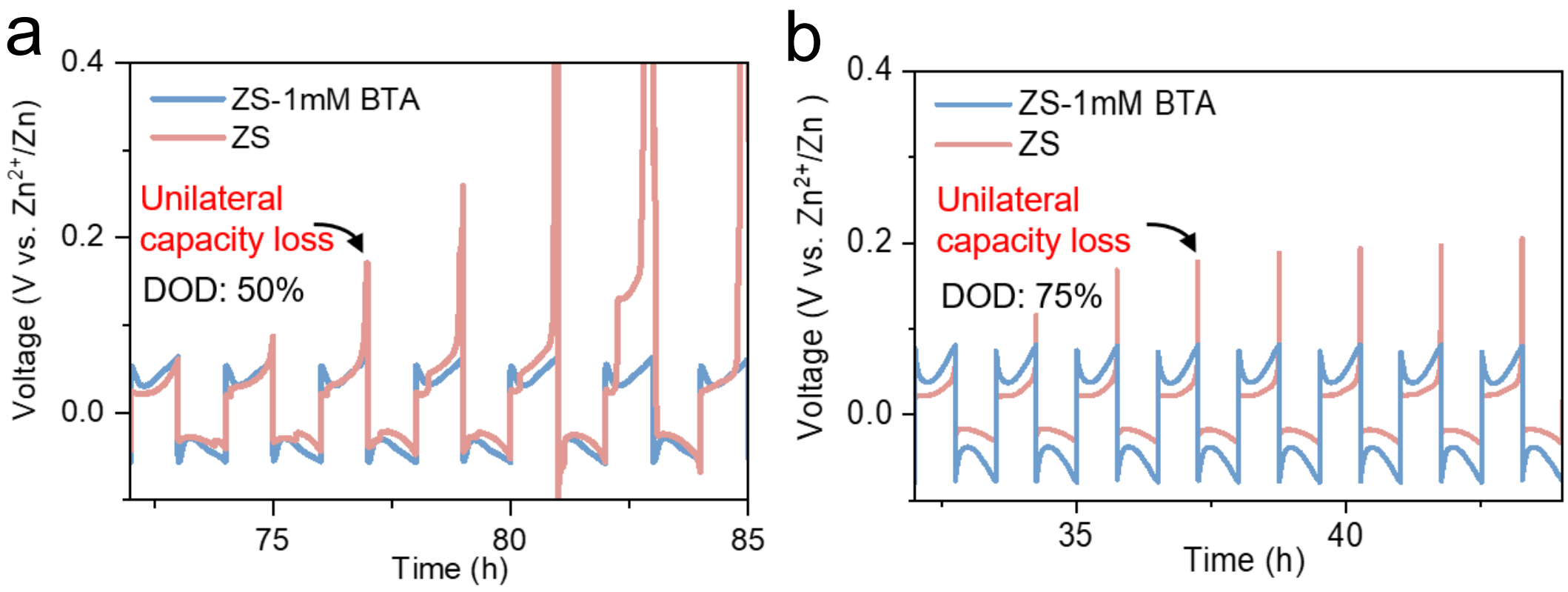

Figure S29 (a) The symmetrical cells of Zn@CuZn@Cu in $ZnSO_4$ and $ZnSO_4$+1mM BTA electrolytes at 1mA cm^{-2} and 1mAh cm^{-2}. (b) The symmetrical cells of Zn@CuZn@Cu in $ZnSO_4$ and $ZnSO_4$+1mM BTA electrolytes at 2mA cm^{-2} and 1.5mAh cm^{-2}.

Note S13: Though, the cells with the bare $ZnSO_4$ electrolyte work stably at the beginning and exhibit a normal voltage curve. However, the sharp increase in voltage polarization occurred prematurely, which means that the lean-zinc electrode has a serious capacity loss (**Figure S29a** and **b**). At this time, the zinc capacity on the substrate can not meet the capacity set by the target program. In addition, we can find that almost all the voltage severe polarization only occurs during the charging period, which means that the electrode located at the positive electrode fails, which is not an accident. The reason for this phenomenon is that when we set the symmetrical cell cycle program, we start from charging, and then discharge, and repeat. Thus, it seems to be a symmetrical cell experiment, but due to the violent depth of discharge, the electrode located on the positive side faces worse working conditions than the negative side.

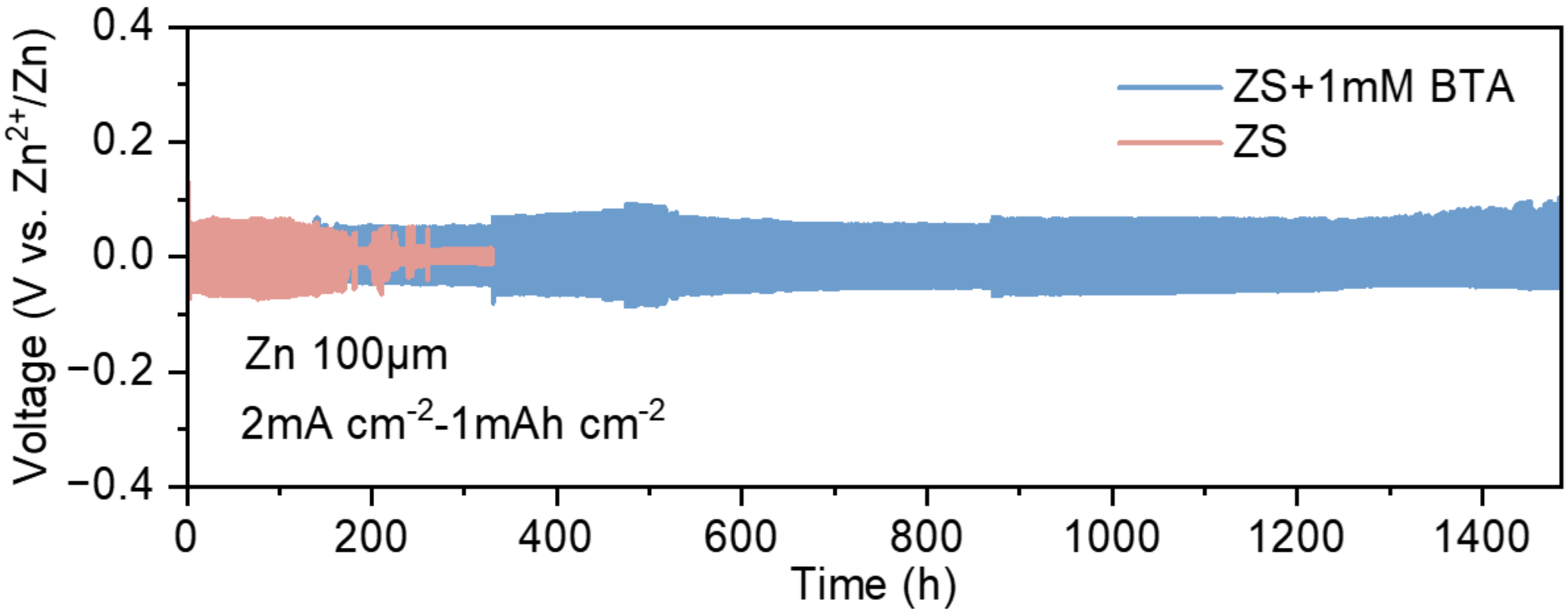

Figure S30 The symmetrical cells of Zn||Zn (100μm) in $ZnSO_4$ and $ZnSO_4$+1mM BTA electrolytes at 2mA cm^{-2} and 1mAh cm^{-2}.

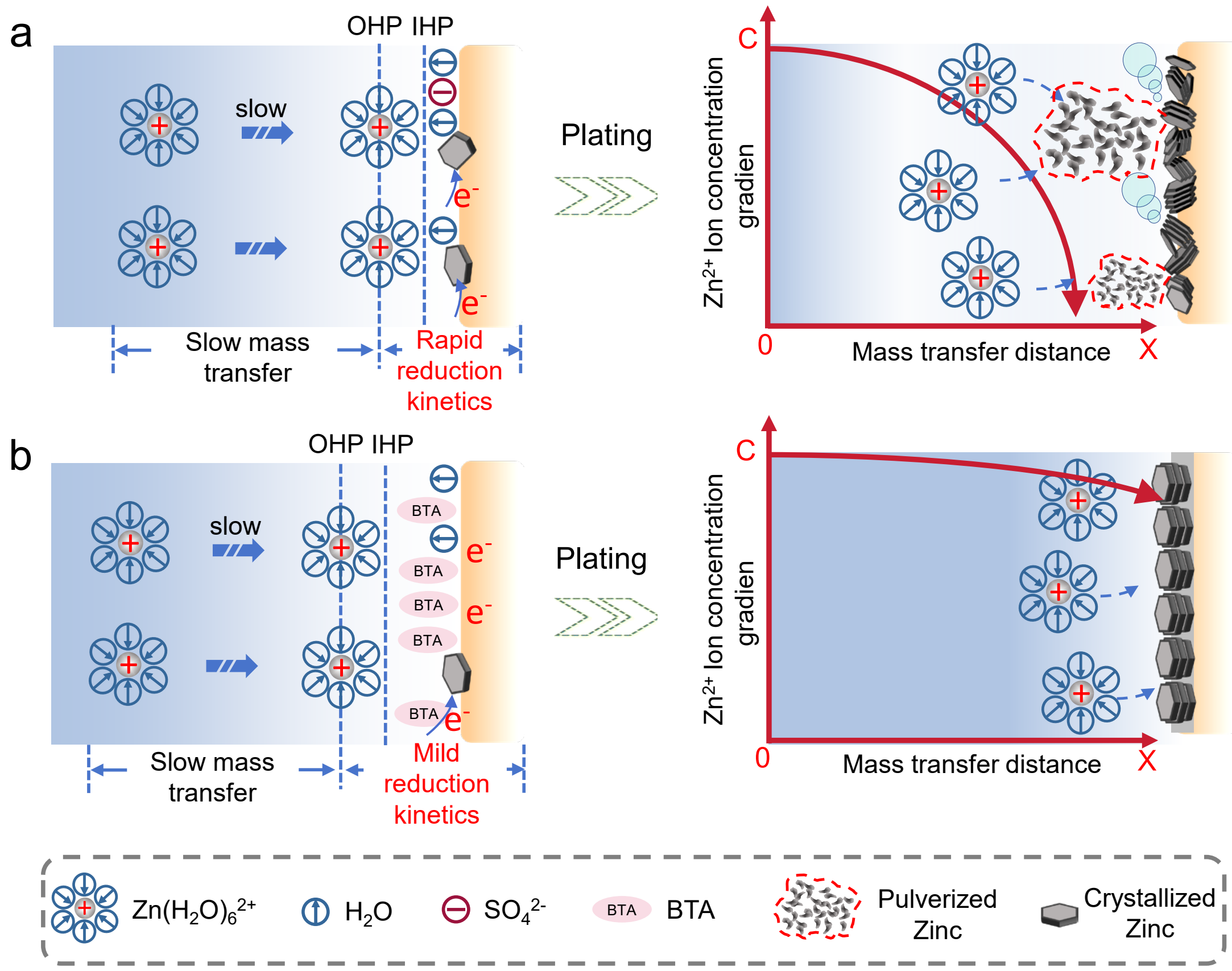

Figure S31 (a) Schematic diagram of zinc electrodeposition growth during concentration polarization process. (b) Schematic diagram of regulating concentration polarization by reducing reduction kinetics with the addition of BTA.

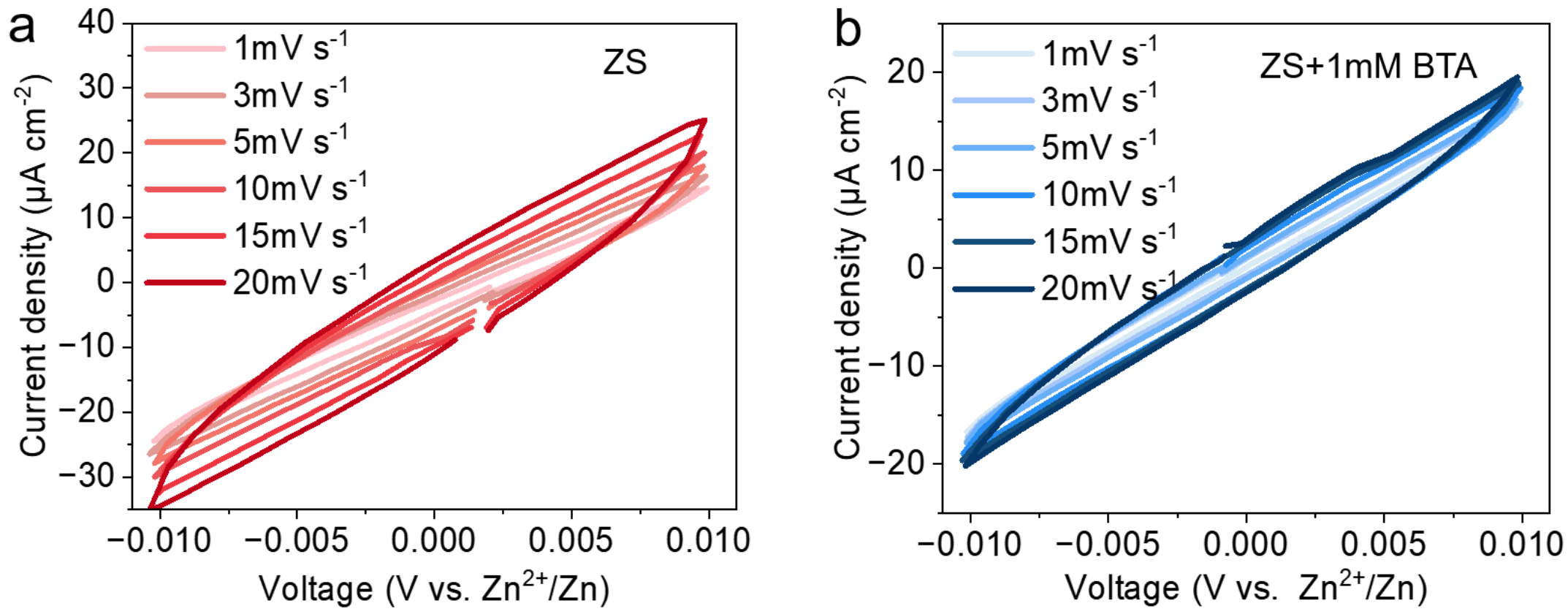

Figure S32 EDL measurements in different electrolytes. CV curves for Zn@CuZn@Cu symmetric cells with pre-depositing 5 mAh cm^{-2} of Zn in $ZnSO_4$ (a) and $ZnSO_4$+1mM BTA (b) electrolyte in a voltage range of -10 to 10 mV under various scanning rates.

Note S14: The capacitance (C) is determined by the linear relationship between capacitive current (i_c) and scan rate (v), which can be obtained from the slope of the i_c versus v curves. Electric double layer capacitances were calculated through the equation of $C = i_c/v$, where C is the capacitance. i_c is the double layer current, v is the scan rate. Here, we choose $i_c = (i_{0+} - i_{0-})/2$.

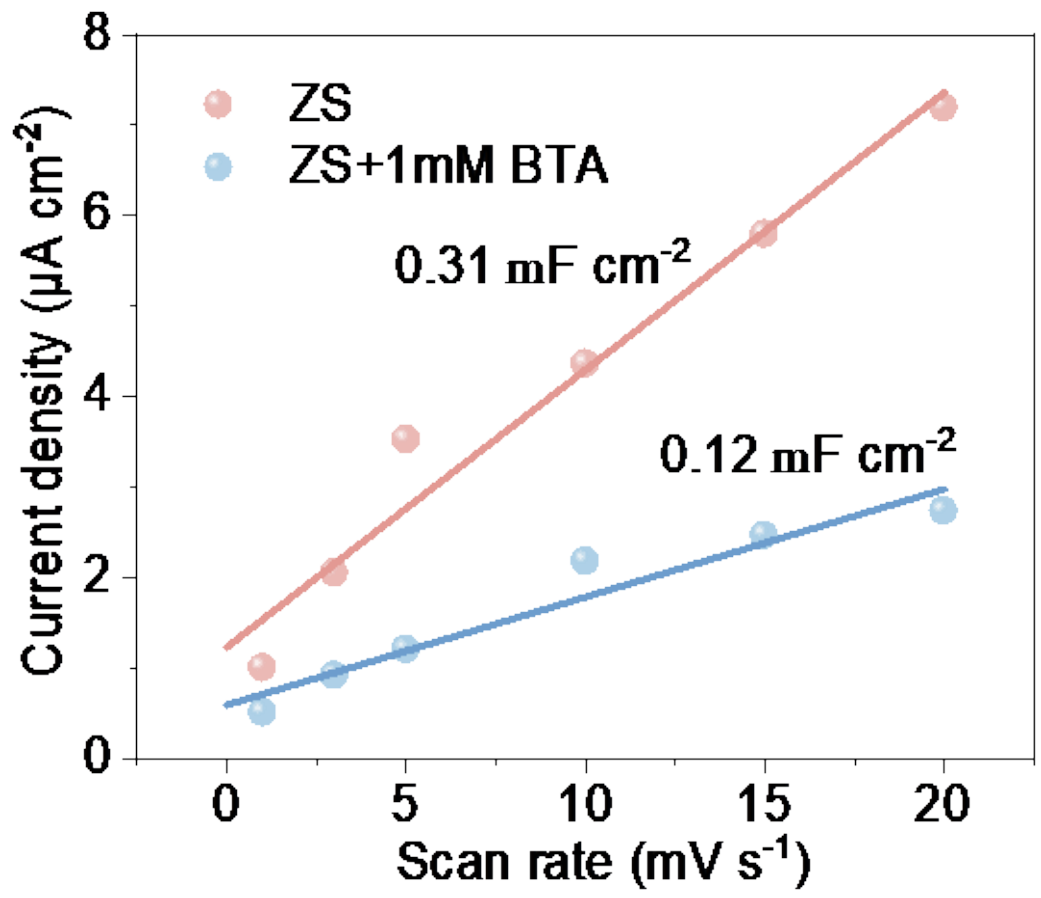

Figure S33 Plots of capacitive currents versus scan rates for the cells cycled in $ZnSO_4$ electrolytes with/without the BTA.

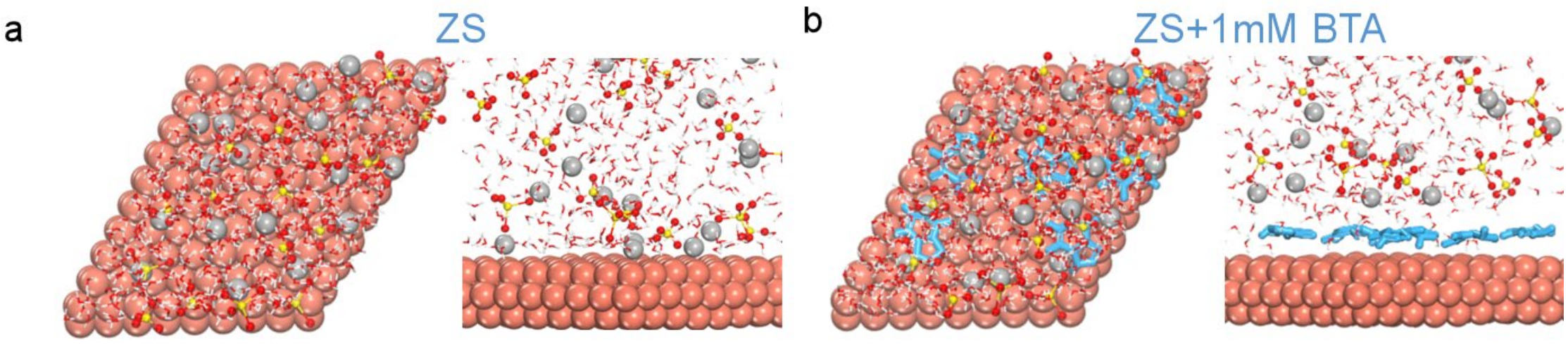

Figure S34 (a) M1 geometry after MD simulations with external voltage U=-1.5 V. M1: $ZnSO_4$/water/Cu substrate. H_2O is shown as thin sticks. O, H, S, Zn and Cu are shown as red, white, yellow, grey and red colors. (b) M2 geometry after MD simulations with copper anode (-1.5 V). M2: $ZnSO_4$+BTA/water/Cu substrate. BTA shown as blue sticks, H_2O as thin sticks. O, H, S, Zn and Cu are shown as red, white, yellow, grey and red colors.

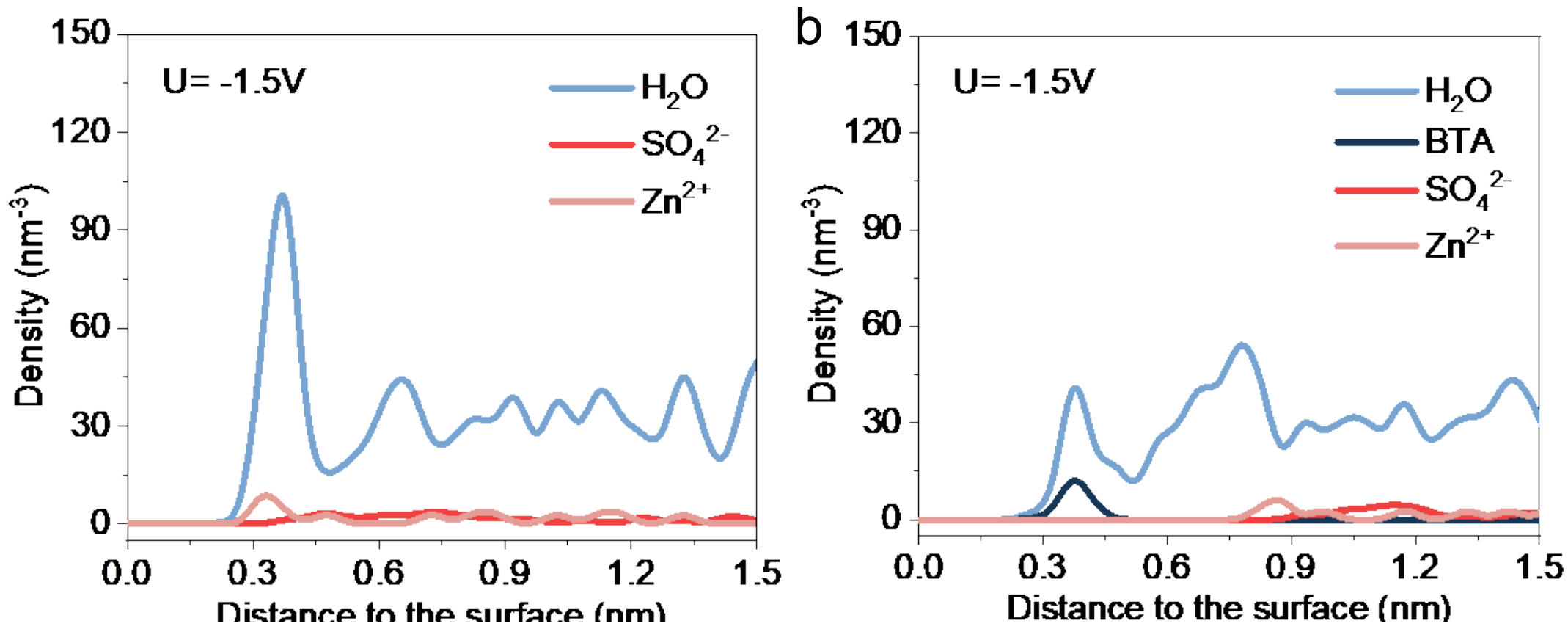

Figure S35 (a) Density profiles as a function of distance from the negative Cu anode (U= -1.5 V) for the SO_4^{2-}, H_2O, and Zn^{2+} in $ZnSO_4$ electrolyte. (b) Density profiles as a function of distance from the negative Cu anode (U=-1.5 V) for the SO_4^{2-}, H_2O, BTA, and Zn^{2+} in $ZnSO_4$+BTA electrolyte.

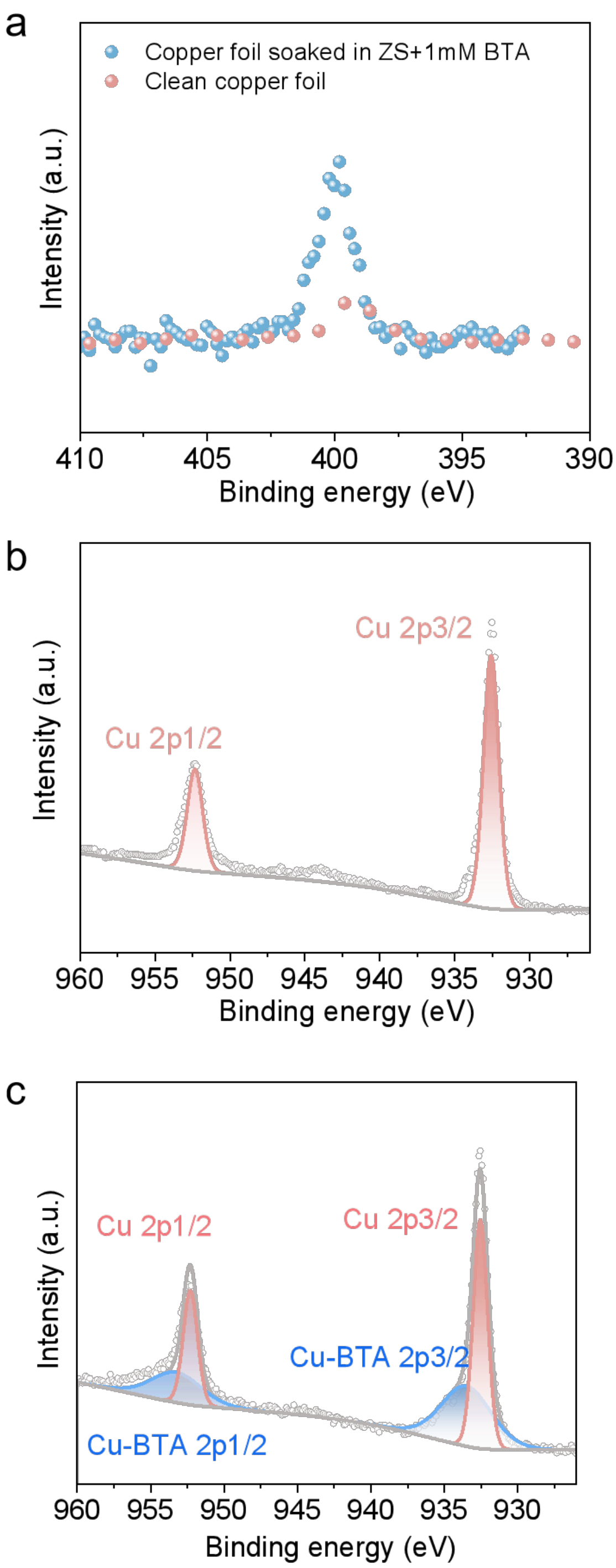

Figure S36 (a) N 1s XPS of the clean Cu foil and the Cu foil soaked in $ZnSO_4$+BTA electrolyte. (b) Cu 2p XPS of bare Cu and Cu soaked in $ZnSO_4$+1mM BTA electrolyte (c).

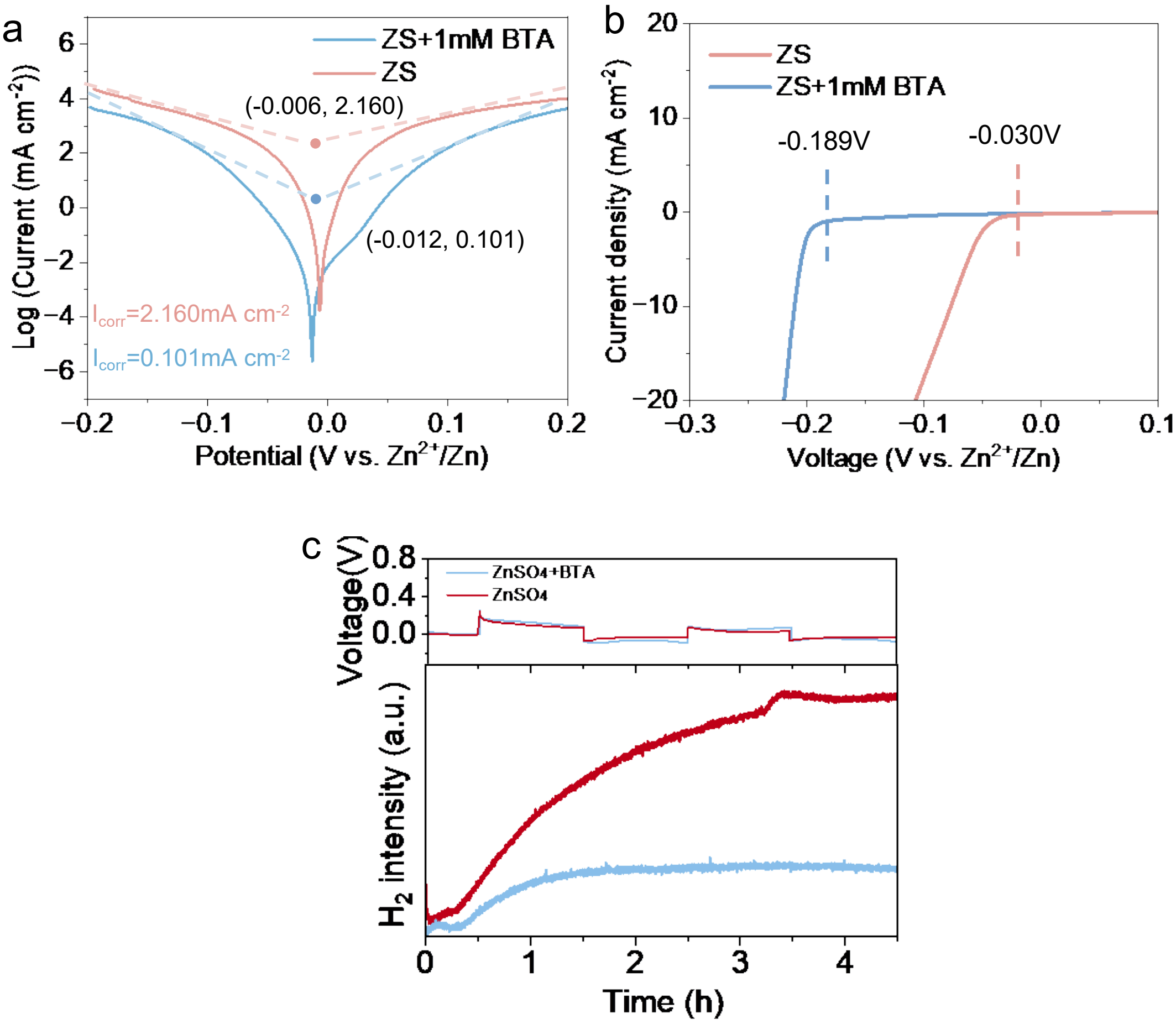

Figure S37 (a) Tafel curves in $ZnSO_4$ and $ZnSO_4$+1mM BTA electrolytes (b) HER tests in $ZnSO_4$ and $ZnSO_4$+1mM BTA electrolytes measured in ZnCu half cells by using LSV at a scan rate of 1 mV s^{-1}. (c) The hydrogen flux and changes in Zn@Cu||Zn@Cu cells with different electrolytes.

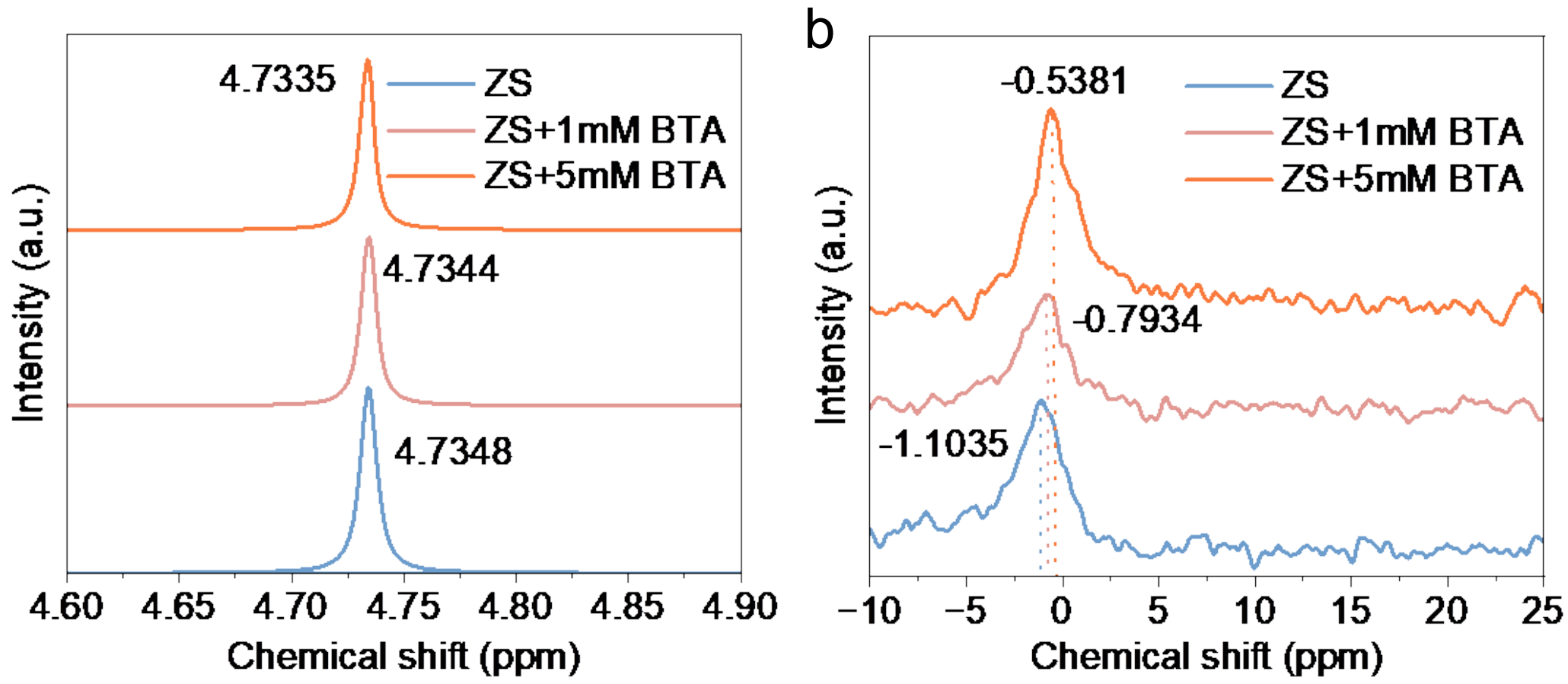

Figure S38 (a) ^{1}H NMR spectra of $ZnSO_4$ and $ZnSO_4$+BTA electrolytes, respectively. (b) ^{67}Zn NMR spectra of $ZnSO_4$ and $ZnSO_4$+BTA electrolytes.

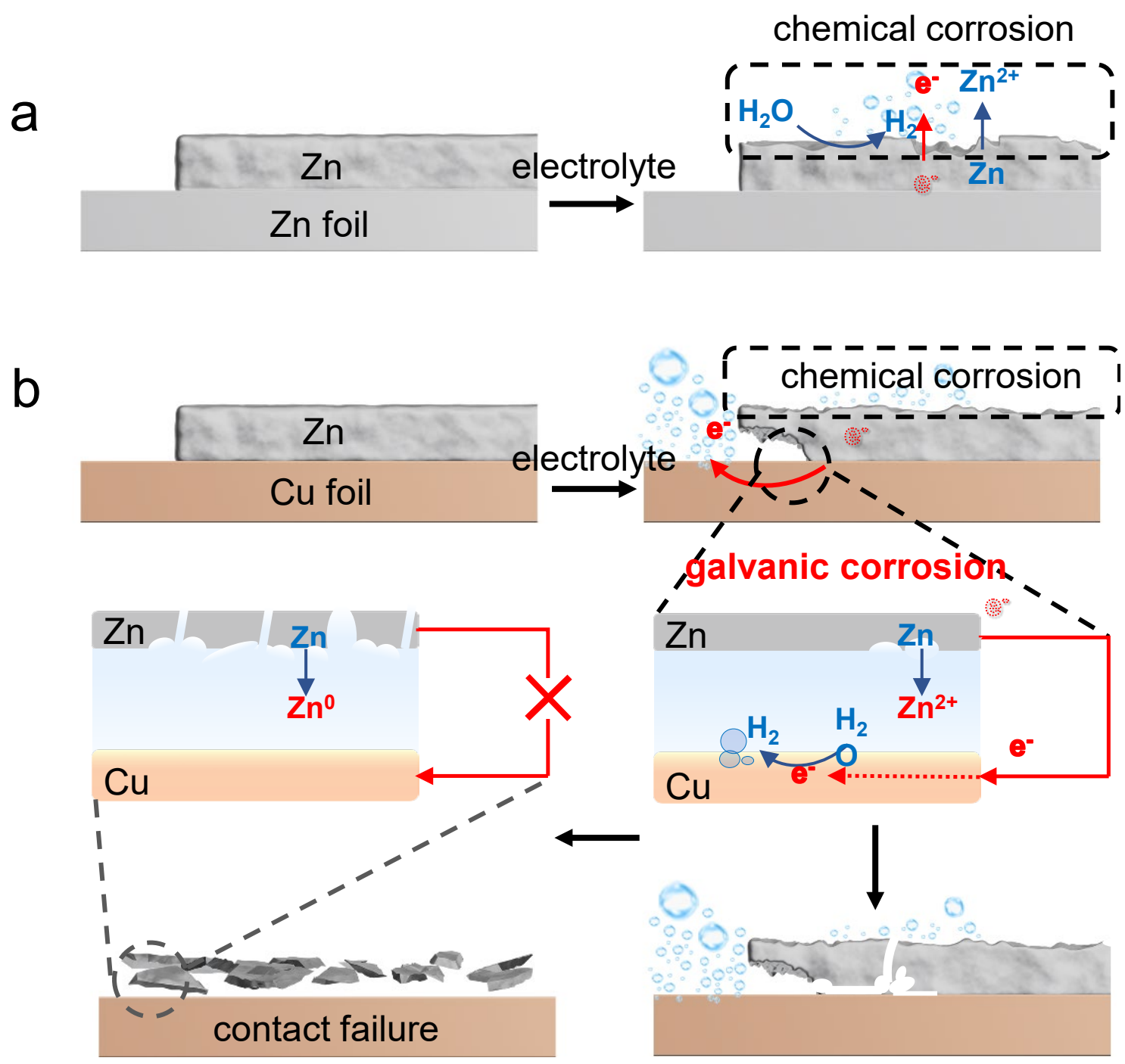

Figure S39 (a) Schematic of Zn@Zn electrode immersion process in $ZnSO_4$ electrolyte. (b) Schematic of lean Zn electrode immersion process in $ZnSO_4$ electrolyte.

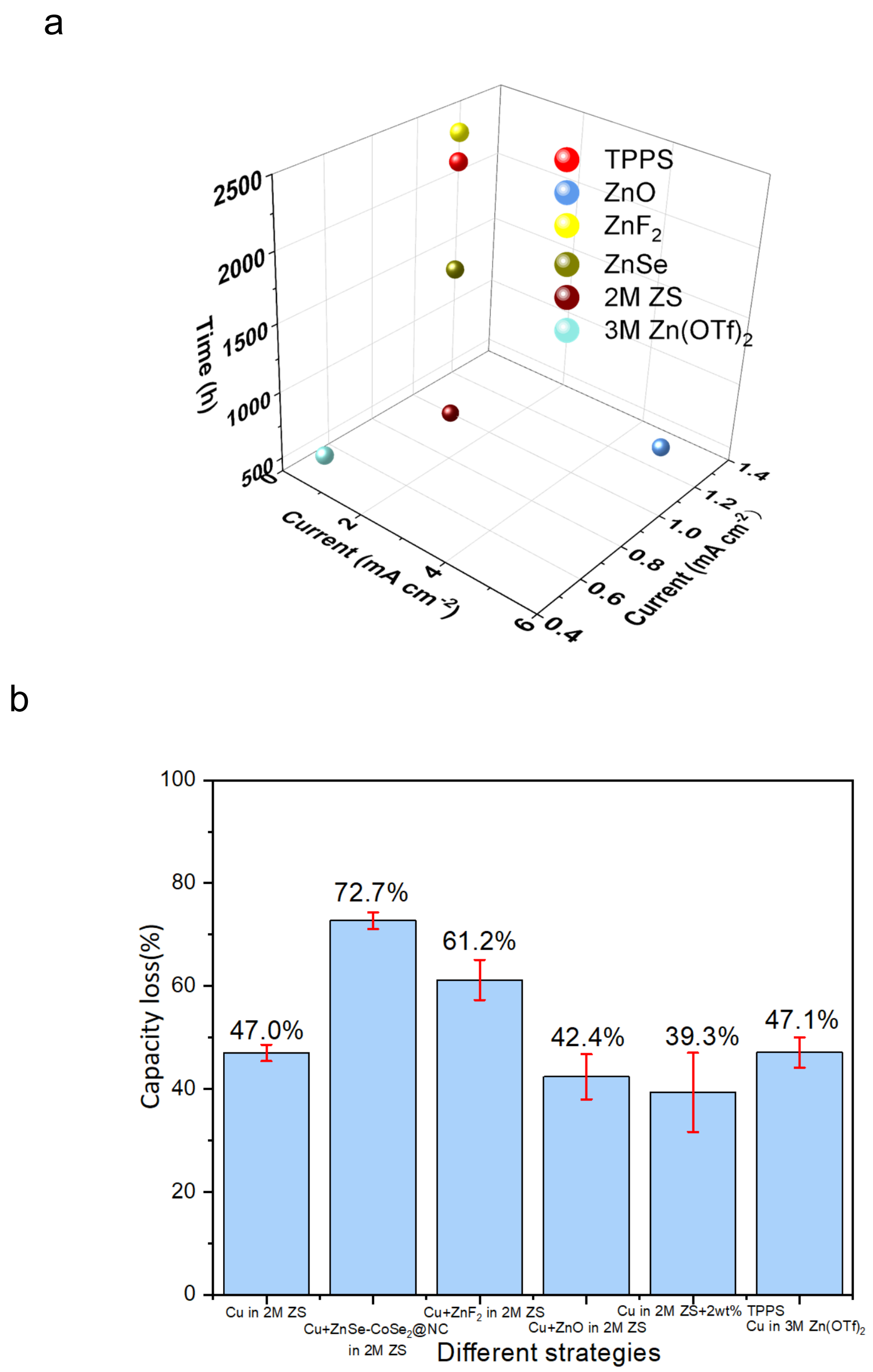

Figure S40 (a) Comparison of the cycling performance of Zn||Zn symmetric cells with previously reported strategies. (b) Comparison of the static capacity loss of lean Zn electrode with previously reported strategies (current density: 1 mA cm^{-2}, capacity: 1 mAh cm^{-2}, temperature: 25 °C, aging time: 24 h). Each bar represents three data points.

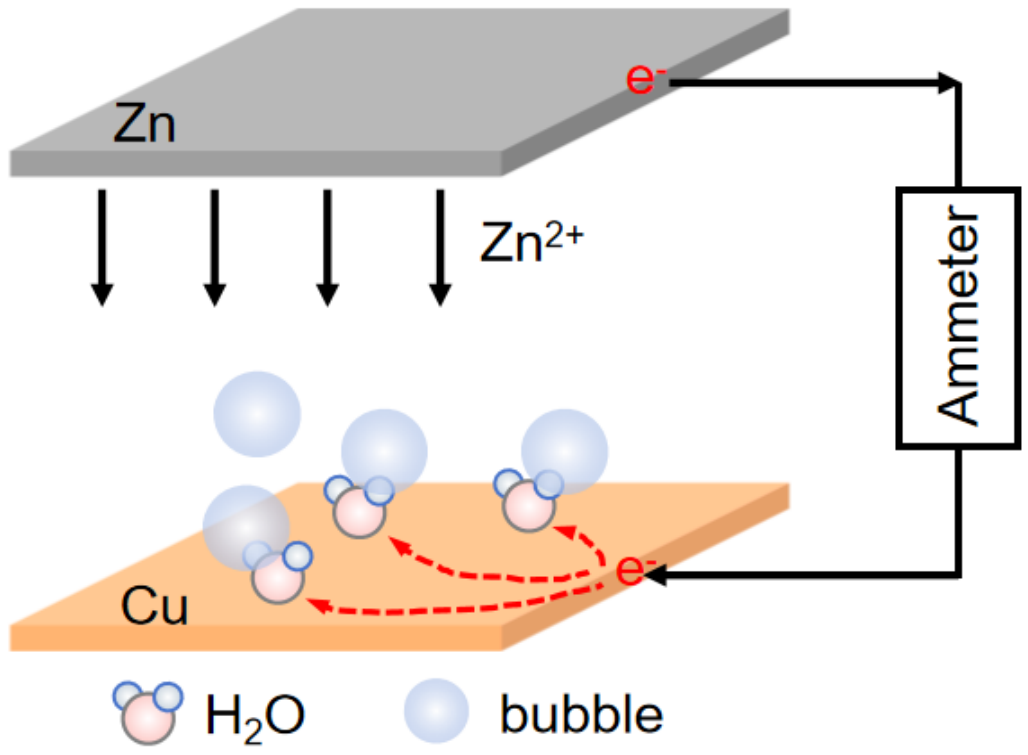

Figure S41 The schematic of the cell quantifying the galvanic corrosion rate.

Note S15: The schematic shows the galvanic cell for quantifying the corrosion rate of Zn during the galvanic processes. Cu and Zn foil are the separated cathode and anode, respectively. Cu was held at the same potential as the Zn foil while the passed current was recorded. The current represents the galvanic corrosion rate of Zn connected to Cu, and is recorded by using an electrochemical workstation.

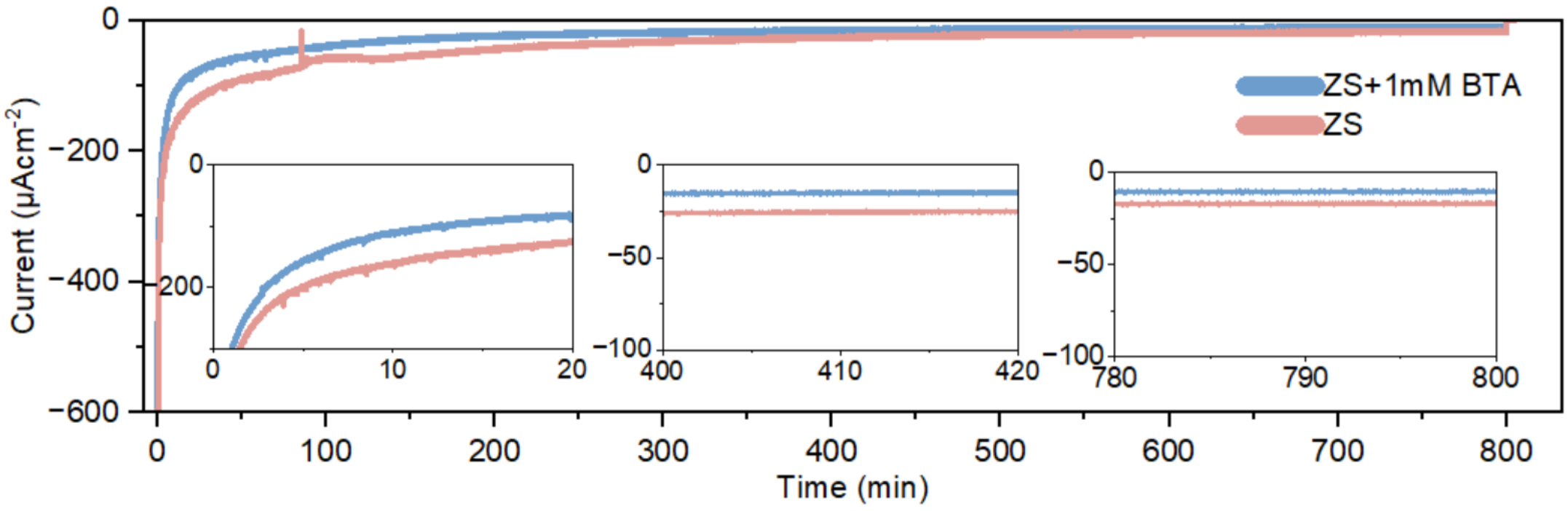

Figure S42 The current density versus time curve for galvanic corrosion current in ZnCu cell in $ZnSO_4$ and $ZnSO_4$+1mM BTA electrolytes.

Note S16: Figure S42 shows the currrent profiles of ZnCu cell after applying a potential step to 0V (vs. Zn/Zn^{2+}) in $ZnSO_4$ and $ZnSO_4$+1mM BTA electrolytes. The sudden decay of initial current and the steady-state current after a long period of time are respectively caused by double layer capacitance and galvanic corrosion[4]. The overall corrosion current for ZnCu cell in $ZnSO_4$+1mM BTA electrolyte is much less than that in bare $ZnSO_4$ electrolyte.

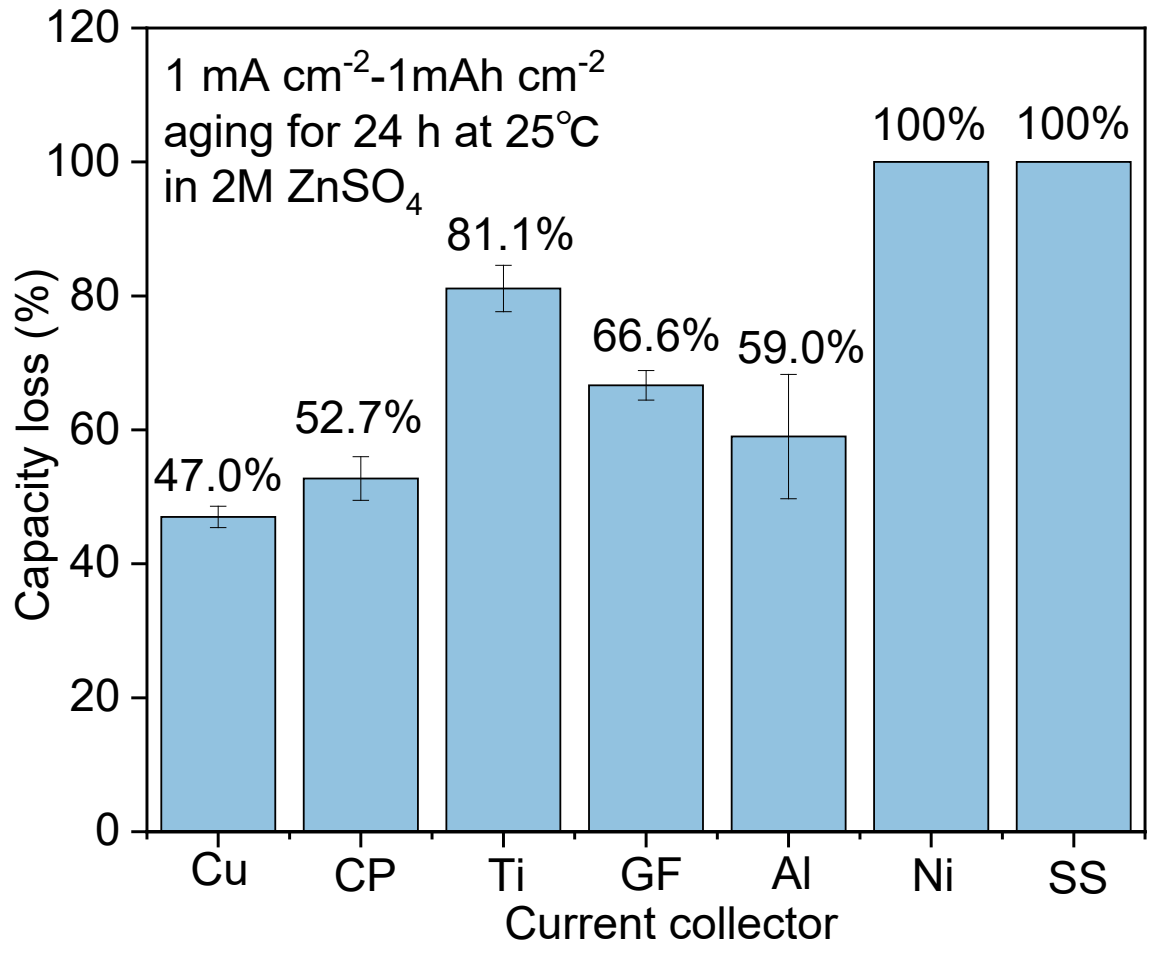

Figure S43 Capacity losses of lean Zn electrodes based on various substrates (current density: 1 mA cm^{-2}, capacity: 1 mAh cm^{-2}, temperature: 25 °C, aging time: 24 h). Each bar represents three data points.

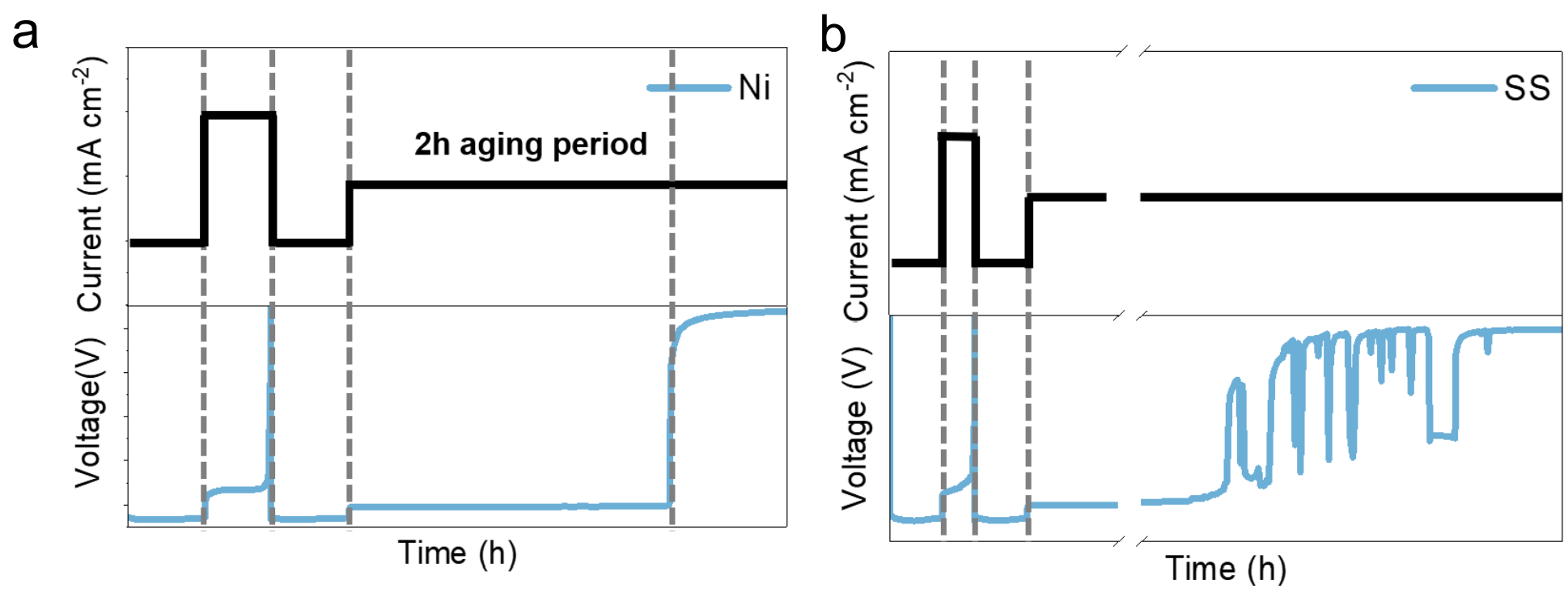

Figure S44 (a) Electrochemical test sequence for quantifying static capacity loss for ZnNi cell. (b) Electrochemical test sequence for quantifying static capacity loss for ZnSS cell.

Note S17: We deposit the same capacity on different substrates and place them under the same temperature conditions with the same electrolyte, which ensures that the electrochemical corrosion rate is consistent. Therefore, the difference in final capacity loss is due to the substrates leading to different electrochemical corrosion rates. When Zn is deposited on the substrate and left to rest, the voltage is maintained at 0V because the two electrodes have the same electrode potential under the electrolyte. However, after depositing Zn on Ni and SS substrates, a sharp increase in voltage was observed after only 2 hours and 10 hours of rest, respectively. This is because during the static process, the potential of the Zn foil electrode on the anode side remains constant, but the Zn@substrates (Zn@Ni, Zn@SS) composite electrode on the cathode will be severely affected by galvanic corrosion, resulting in a gradual loss of Zn capacity. When the zinc on the composite electrode is completely lost, the electrode potential (Zn@Ni, Zn@SS) on the cathode side increases to the original electrode potential of the substrate (Ni, SS), resulting in a sharp increase in the external voltage of the cell during the static process.

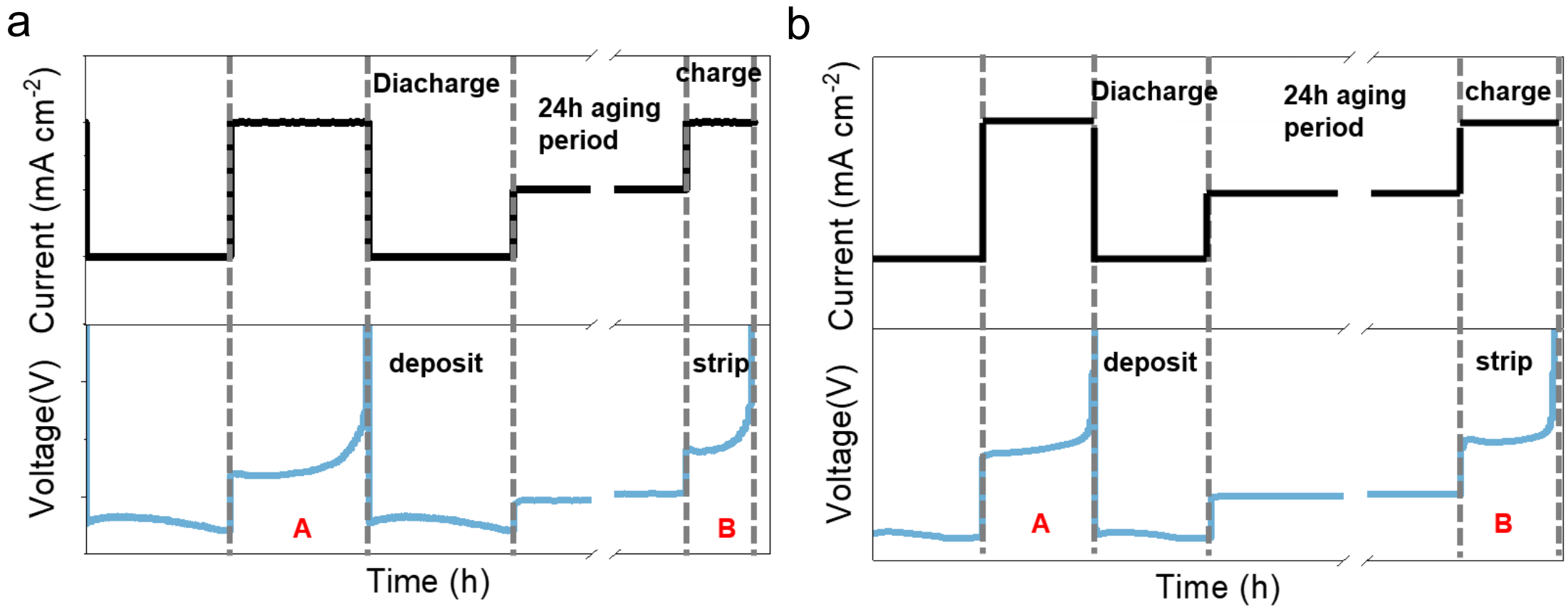

Figure S45 Electrochemical test sequence for quantifying galvanic corrosion loss for ZnCu cell in $ZnSO_4$ (**a**) and $ZnSO_4$+1mM BTA (**b**)

$$C_{loss} = \frac{C_{stripA} - C_{stripB}}{C_{stripA}}$$

Where C_{loss} represents the static capacity loss, C_{stripA} is the capacity that is completely stripped before rest, and C_{stripB} is the capacity that is completely stripped after rest.

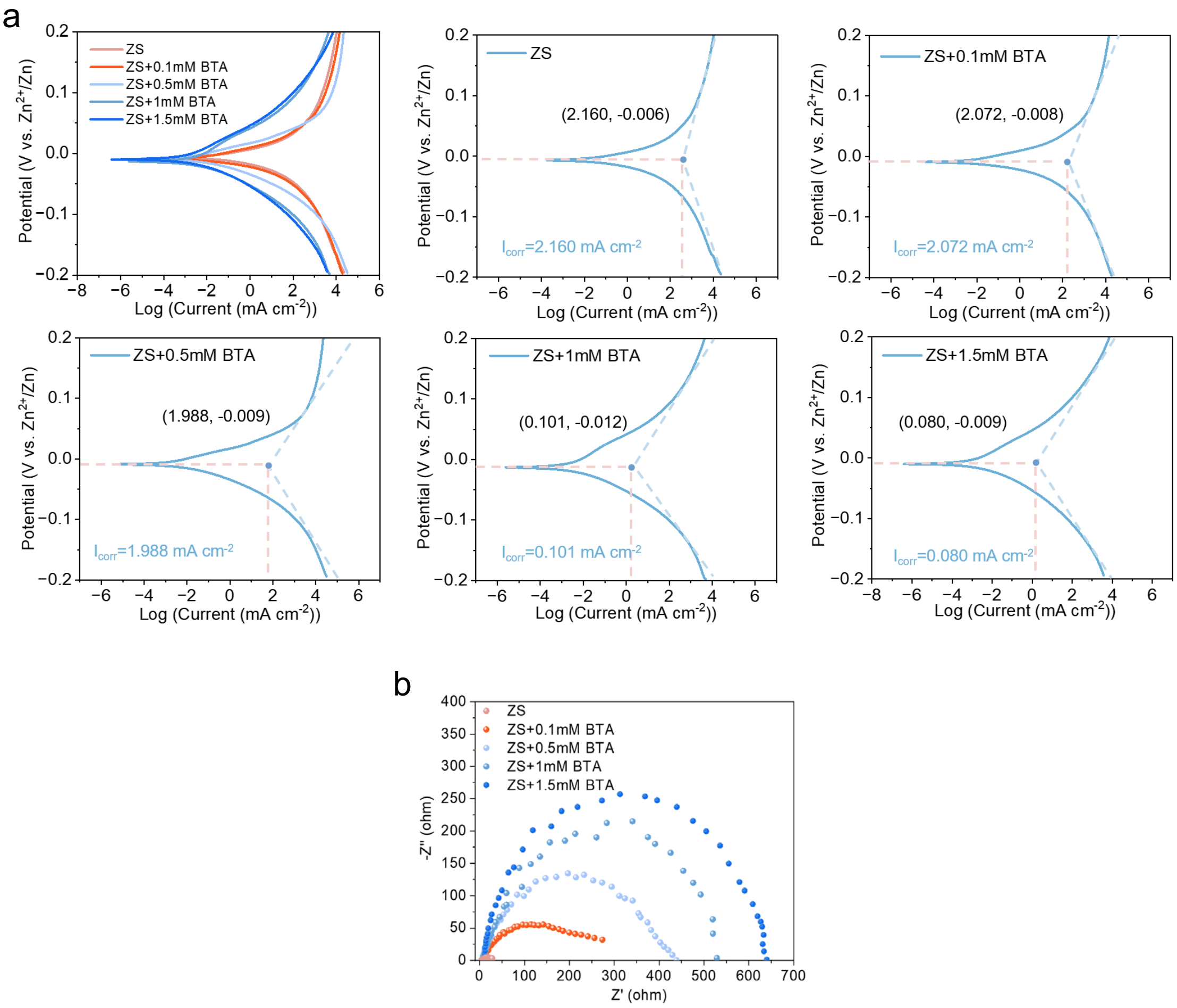

Figure S46 (a) Tafel curves of $ZnSO_4$ and $ZnSO_4$+BTA electrolytes (BTA=0.1mM, 0.5mM, 1mM, and 1.5mM). (b) Impedance spectra (presented as Nyquist plots) of lean Zn anodes in $ZnSO_4$ and $ZnSO_4$+BTA electrolyte (BTA=0.1mM, 0.5mM, 1mM, and 1.5mM).

Table S1 Fitted parameters of **Figure S46**

ρ (BTA)/mM L^{-1}	E_{coor} (vs. Zn^{2+}/Zn)/V	I_{corr}/(mA cm^{-2})	η/%
0	-0.006	2.160	/
0.1	-0.008	2.072	4.0%
0.5	-0.009	1.988	7.0%
1	-0.012	0.101	97.0%
1.5	-0.009	0.080	99.3%

$$\eta = \left(1 - I_{corr} / I'_{corr}\right) \times 100\%$$

where η is the corrosion inhibition efficiency, I'_{corr}, and I_{corr} are the corrosion current density before and after corrosion inhibition treatment, respectively.

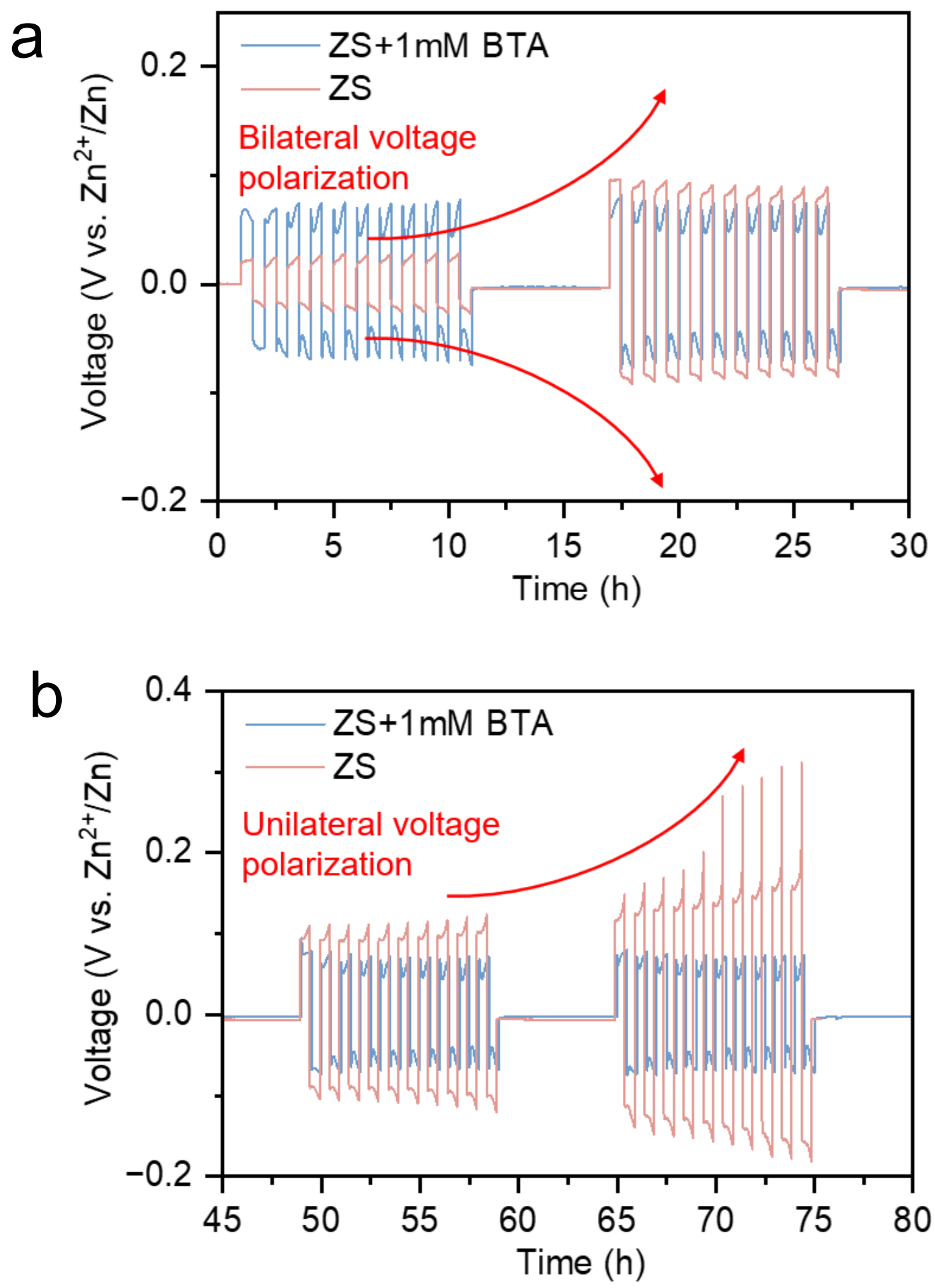

Figure S47 Partial enlarged image of electrochemical performance of the symmetrical cells when enduring alternate cycling (1 mA cm^{-2} and 0.5 mAh cm^{-2}) and resting process (6h). (a) Bilateral voltage polarization. (b) Unilateral voltage polarization

Note S18: As shown in **Figure S47a**, the voltage difference between charging and discharging significantly increases before and after the first static state in $ZnSO_4$ electrolyte. It should be noted that at this point, the voltage polarization is symmetrical, indicating that it is not caused by capacity loss, but by an increase in cell internal resistance. In contrast, after the fourth resting period, the voltage polarization becomes significantly asymmetric (**Figure S47b**), with a peak voltage appearing on the charging side. This is akin to the emergence of a half-cell voltage signal, suggesting that one side of the electrode has undergone complete zinc stripping, indicating a severe loss of capacity.

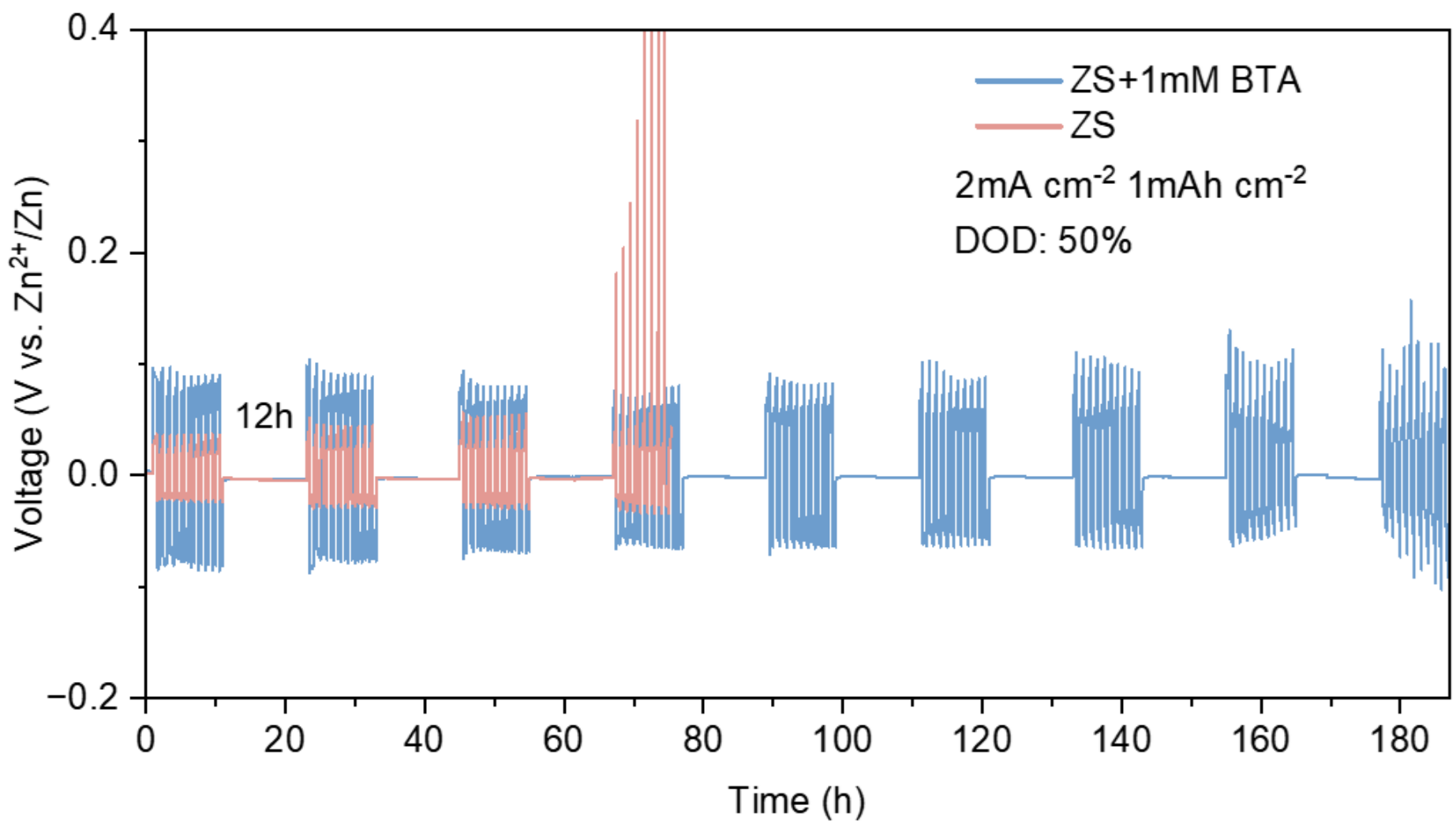

Figure S48 Electrochemical performance of the Zn@CuZn@Cu symmetrical cells when enduring alternate cycling (2 mA cm^{-2} and 1 mAh cm^{-2}) and resting process (12 h).

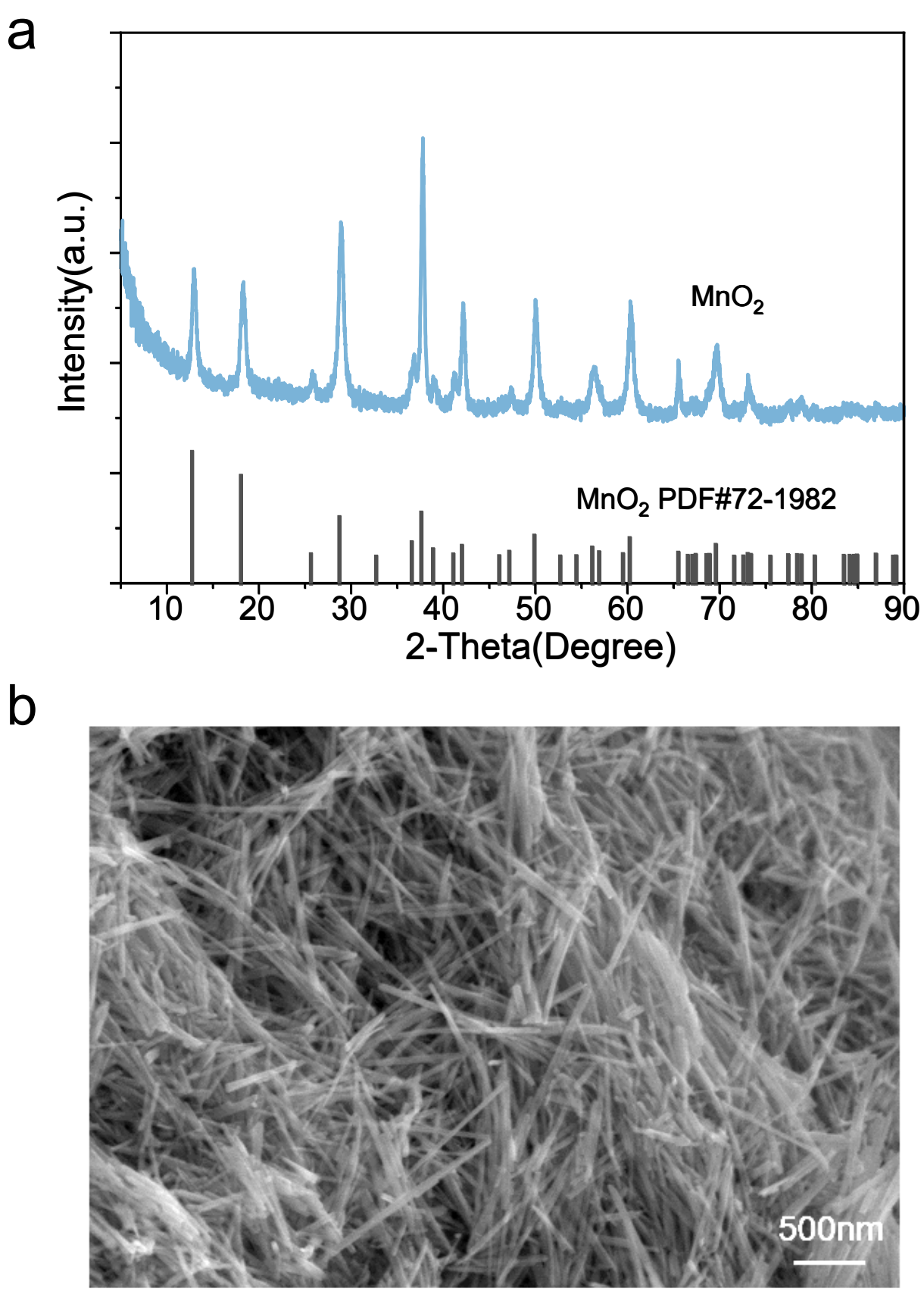

Figure S49 XRD pattern (a) and SEM images (b) of MnO_2 material.

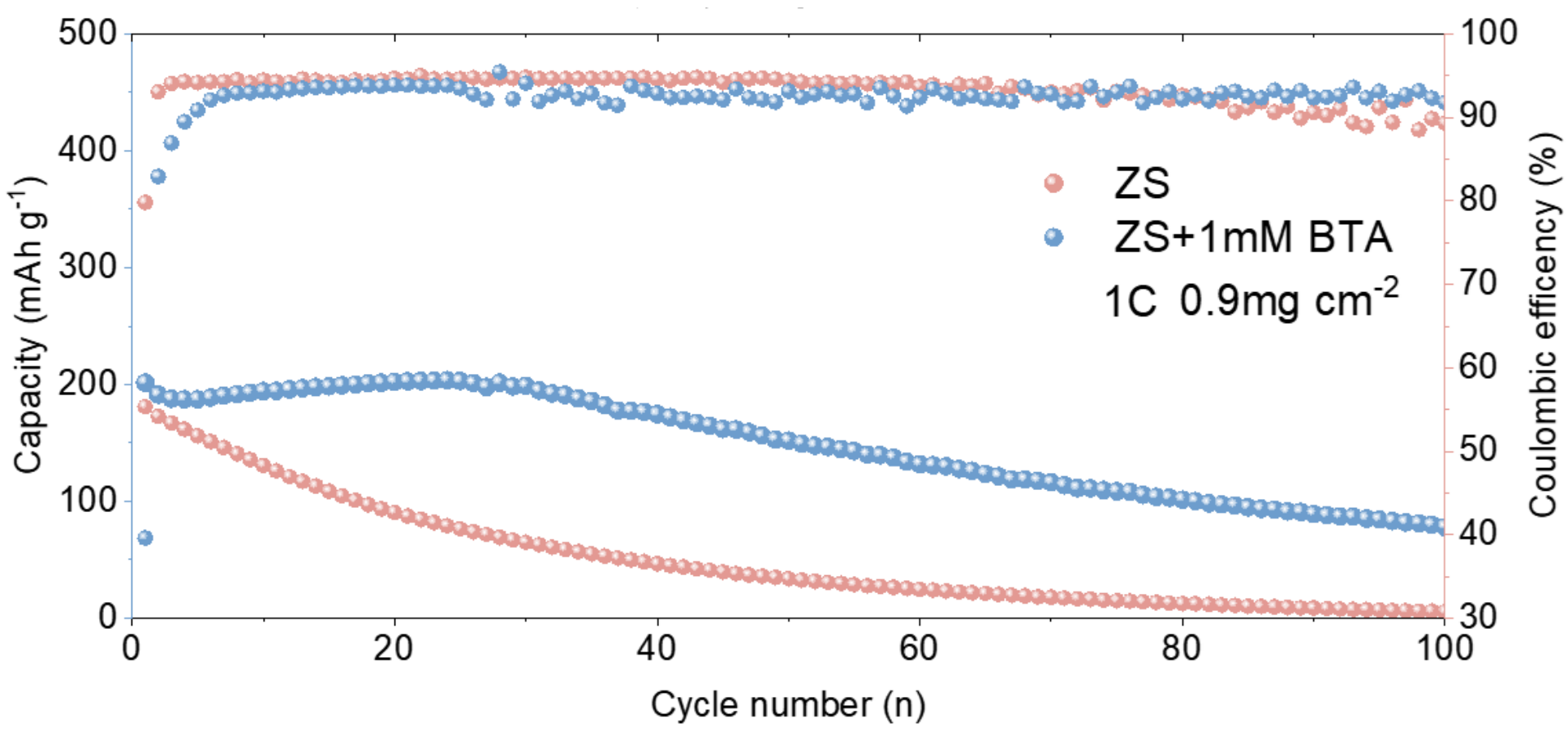

Figure S50 Cycle performance of the anode-free zinc batteries in $ZnSO_4$ and $ZnSO_4$+BTA electrolyte.

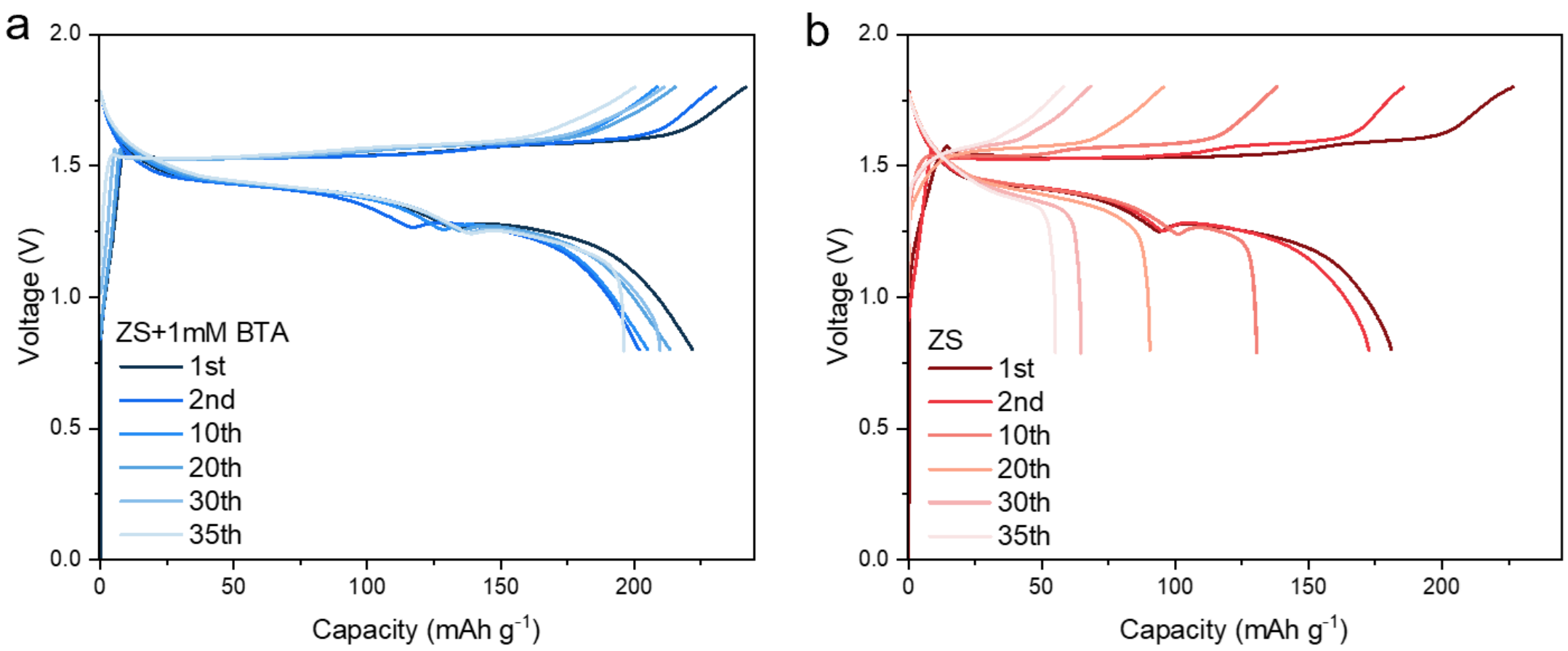

Figure S51 Charge/discharge curves for anode-free zinc batteries (1C 0.9mg cm^{-2}) with $ZnSO_4$+1mM BTA (a) and $ZnSO_4$ (b) electrolytes.

Note S19: Prior to the assembly of an anode-free battery, the MnO_2 electrode was pre-zincificated in a cell with Zn metal, glass fiber separator, and a mixed electrolyte of 2M $ZnSO_4$ and 0.1M $MnSO_4$. The pre-zincificated MnO_2 electrode was then washed, dried, and paired with the Cu substrate using a separator and 2M $ZnSO_4$+0.1M $MnSO_4$ electrolyte with/without BTA. Subsequently, it was assembled into the coin cell.

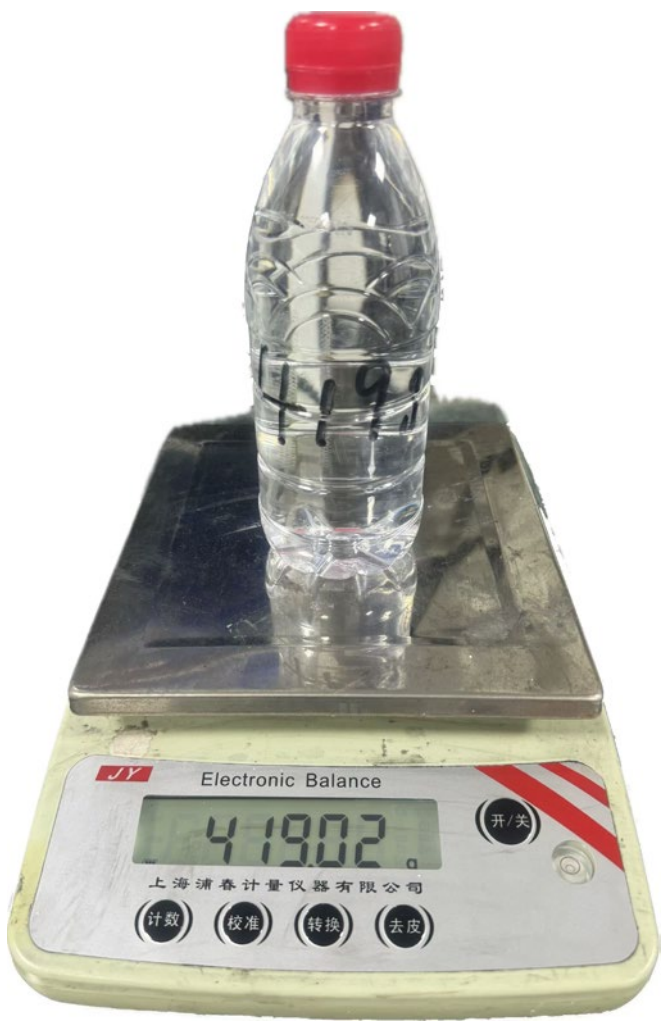

Figure S52 Weight test for the bottle of water loaded on the toy car

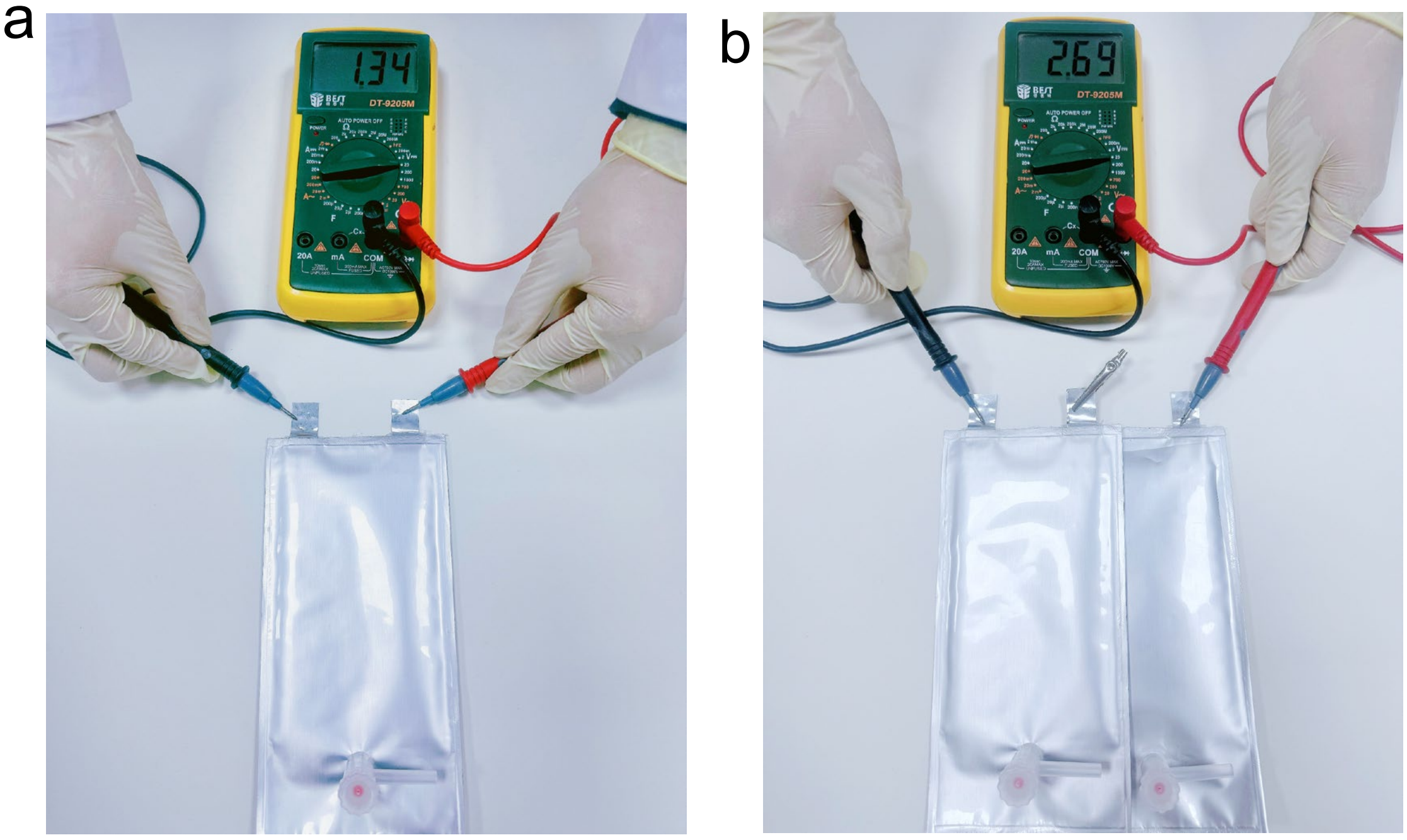

Figure S53 Voltage tests for the 1Ah pouch $Zn@CuMnO_2$ battery.

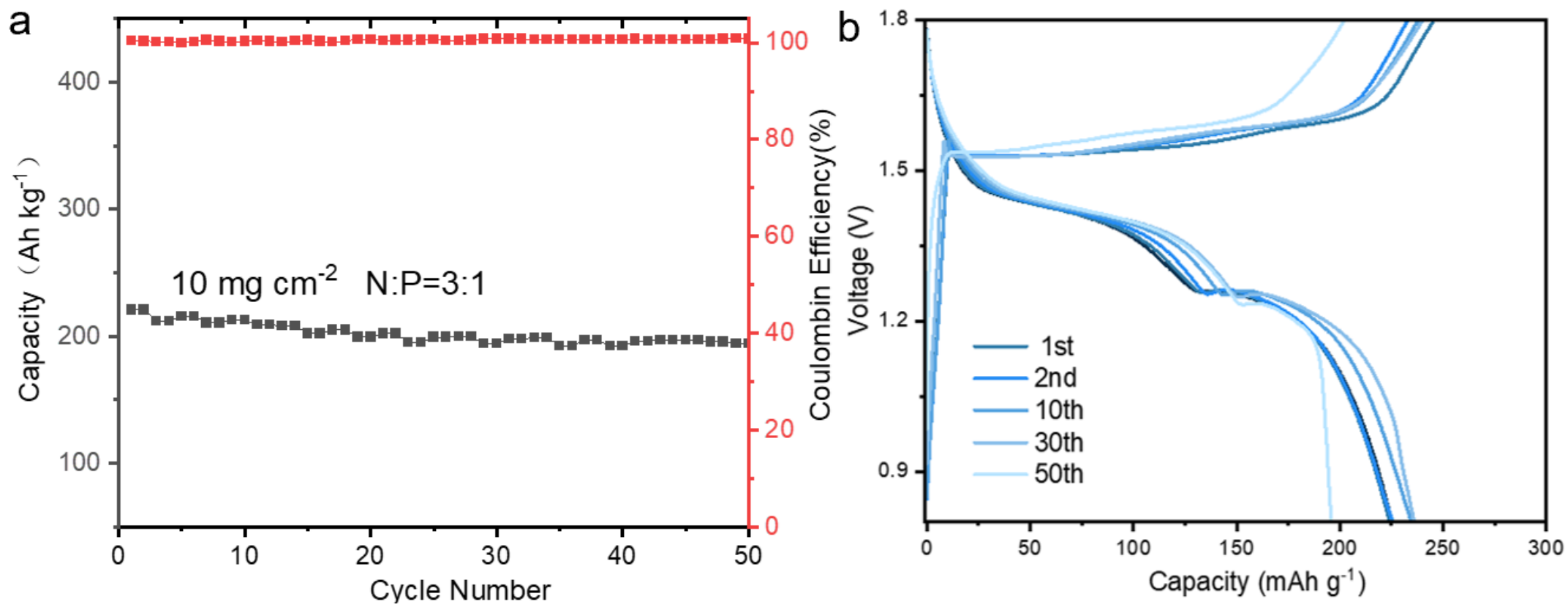

Figure S54 The cycling performance of 1Ah Zn@$CuMnO_2$ pouch cell based on the $ZnSO_4$+BTA electrolyte.

1. Deng C*, et al.* Nature-inspired interfacial engineering for highly stable Zn metal anodes. *Energy Storage Mater* **58**, 279-286 (2023).

2. Xu J*, et al.* Heterostructure ZnSe-$CoSe_2$ embedded with yolk-shell conductive dodecahedral as Two-in-one hosts for cathode and anode protection of Lithium–Sulfur full batteries. *Energy Storage Mater* **47**, 223-234 (2022).

3. Yang S*, et al.* Regulating the electrochemical reduction kinetics by the steric hindrance effect for a robust Zn metal anode. *Energy Environ Sci* **17**, 1095-1106 (2024).

4. Zhehan Yi, Jiaxin Liu, Shandong Tan, Zhiyuan Sang, Jing Mao, Lichang Yin, Xiaoguang Liu, Liqun Wang, Feng Hou, Shi Xue Dou, Hui-Ming Cheng, Ji Liang, Adv. Mater. 2022, 34, 2203835

5. Tengsheng Zhang, Yan Tang, Shan Guo, Xinxin Cao, Anqiang Pan, Guozhao Fang, Jiang Zhou, Shuquan Liang, *Energy Environ. Sci.,* 2020,13, 4625

6. Lin Ma, Marshall A. Schroeder, Oleg Borodin, Travis P. Pollard, Michael S. Ding, Chunsheng Wang, Kang Xu, *Nature Energy* 2020, 5, 743–749
